# Progressive liver, kidney, and heart degeneration in children and adults affected by *TULP3* mutations

**DOI:** 10.1016/j.ajhg.2022.03.015

**Published:** 2022-04-08

**Authors:** John Devane, Elisabeth Ott, Eric G. Olinger, Daniel Epting, Eva Decker, Anja Friedrich, Nadine Bachmann, Gina Renschler, Tobias Eisenberger, Andrea Briem-Richter, Enke Freya Grabhorn, Laura Powell, Ian J. Wilson, Sarah J. Rice, Colin G. Miles, Katrina Wood, Genomics England Research Consortium, Palak Trivedi, Gideon Hirschfield, Andrea Pietrobattista, Elizabeth Wohler, Anya Mezina, Nara Sobreira, Emanuele Agolini, Giuseppe Maggiore, Mareike Dahmer-Heath, Ali Yilmaz, Melanie Boerries, Patrick Metzger, Christoph Schell, Inga Grünewald, Martin Konrad, Jens König, Bernhard Schlevogt, John A. Sayer, Carsten Bergmann

**Affiliations:** 1 Department of Medicine IV, Faculty of Medicine, Medical Center-University of Freiburg, 79106 Freiburg, Germany; 2 Translational and Clinical Research Institute, Faculty of Medical Sciences, Newcastle University, Newcastle upon Tyne NE1 3BZ, UK; 3 Medizinische Genetik Mainz, Limbach Genetics, 55128 Mainz, Germany; 4 University Medical Center Hamburg-Eppendorf, Department of Pediatrics, 20251 Hamburg, Germany; 5 Biosciences Institute, Faculty of Medical Sciences, Newcastle University, Newcastle upon Tyne NE1 3BZ, UK; 6 Histopathology Department, The Newcastle upon Tyne Hospitals NHS Foundation Trust, Newcastle upon Tyne NE1 4LP, UK; 7 NIHR Birmingham BRC, Centre for Liver and Gastrointestinal Research, University of Birmingham, Birmingham B15 2TT, UK; 8 Liver Unit, University Hospitals Birmingham, Birmingham B15 2GW, UK; 9 Institute of Immunology and Immunotherapy, University of Birmingham, Birmingham B15 2TT, UK; 10 Institute of Applied Health Research, University of Birmingham, Birmingham B15 2TT, UK; 11 Toronto Centre for Liver Disease, University Health Network, Toronto, ON M6H 3M1, Canada; 12 Hepatogastroenterology and Liver Transplant Unit and Medical Genetics Laboratory, IRCCS Bambino Gesù Children’s Hospital, 00165 Rome, Italy; 13 Translational Cytogenomics Research Unit, Bambino Gesù Children’s Hospital, IRCCS, 00146 Rome, Italy; 14 McKusick-Nathans Department of Genetic Medicine, Johns Hopkins University School of Medicine, Baltimore, MD 21205, USA; 15 Department of Medicine, University of Pennsylvania Perelman School of Medicine, Philadelphia, PA 19104, USA; 16 Department of General Pediatrics, University Hospital Münster, 48149 Münster, Germany; 17 Department of Cardiology I, University Hospital Münster, 48149 Münster, Germany; 18 Institute of Medical Bioinformatics and Systems Medicine Medical Center – University of Freiburg, Medical Faculty, University of Freiburg, 79110 Freiburg, Germany; 19 The German Cancer Consortium, Partner Site Freiburg and Cancer Research Center, 69120 Heidelberg, Germany; 20 Institute for Pathology, Medical Center – University of Freiburg, Medical Faculty, University of Freiburg, 79002 Freiburg, Germany; 21 Institute for Pathology, University Hospital Münster, 48149 Münster, Germany; 22 Department of Internal Medicine B, Gastroenterology, University Hospital Münster, 48149 Münster, Germany; 23 Renal Services, The Newcastle upon Tyne Hospitals NHS Foundation Trust, Newcastle upon Tyne NE7 7DN, UK; 24 Newcastle Biomedical Research Centre, NIHR, Newcastle upon Tyne NE4 5PL, UK

**Keywords:** organ fibrosis, genetic disease burden, internal medicine genetics, ciliopathy, tubby-like proteins, kidney failure, liver fibrosis, hypertrophic cardiomyopathy

## Abstract

Organ fibrosis is a shared endpoint of many diseases, yet underlying mechanisms are not well understood. Several pathways governed by the primary cilium, a sensory antenna present on most vertebrate cells, have been linked with fibrosis. Ciliopathies usually start early in life and represent a considerable disease burden. We performed massively parallel sequencing by using cohorts of genetically unsolved individuals with unexplained liver and kidney failure and correlated this with clinical, imaging, and histopathological analyses. Mechanistic studies were conducted with a vertebrate model and primary cells. We detected bi-allelic deleterious variants in *TULP3*, encoding a critical adaptor protein for ciliary trafficking, in a total of 15 mostly adult individuals, originating from eight unrelated families, with progressive degenerative liver fibrosis, fibrocystic kidney disease, and hypertrophic cardiomyopathy with atypical fibrotic patterns on histopathology. We recapitulated the human phenotype in adult zebrafish and confirmed disruption of critical ciliary cargo composition in several primary cell lines derived from affected individuals. Further, we show interaction between TULP3 and the nuclear deacetylase SIRT1, with roles in DNA damage repair and fibrosis, and report increased DNA damage *ex vivo*. Transcriptomic studies demonstrated upregulation of profibrotic pathways with gene clusters for hypertrophic cardiomyopathy and WNT and TGF-β signaling. These findings identify variants in *TULP3* as a monogenic cause for progressive degenerative disease of major organs in which affected individuals benefit from early detection and improved clinical management. Elucidation of mechanisms crucial for DNA damage repair and tissue maintenance will guide novel therapeutic avenues for this and similar genetic and non-genomic diseases.

## Graphical abstract

**Figure:**
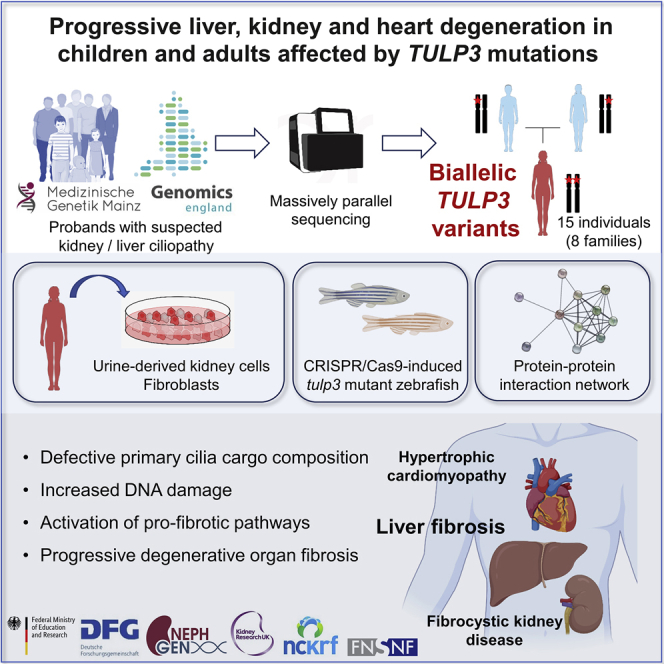

## Introduction

Fibrosis is the result of maladaptive processes leading to an excessive accumulation and deposition of extracellular matrix (ECM) and connective tissue and often culminates in large scale disruption of tissue architecture. Chronic fibrosis of organs can lead to progressive decline in function as ECM slowly replaces parenchymal tissue and may result in organ failure over many years.^1^^,^^2^

The mechanism of fibrosis is closely linked to normal wound healing (reviewed in Rockey et al.^3^) and usually involves both intrinsic susceptibility and predisposing factors such as exposure to genotoxins or aging.^4^ Fibrosis is estimated to be a contributing factor in 45% of deaths in the United States.^5^ Monogenic diseases linked to fibrosis offer a unique opportunity to untangle intrinsic pathways from external factors and prioritize potential therapeutic targets.^4^ High-throughput technologies have allowed the detection of pathogenic mechanisms associated with progressive organ fibrosis, including DNA damage,^6^ storage disorders,^7^ and defective protein synthesis.^8^ However, there is still comparatively little known about distinct underlying genetic mechanisms.

Dysfunction of a cell signaling organelle known as the primary cilium is at the origin of a group of human diseases referred to as ciliopathies that are also characterized by multisystem organ fibrosis via alterations in different molecular pathways.^9^ Interestingly, several ciliary disease proteins colocalize to sites of DNA damage, linking a subset of ciliopathies with aberrant DNA damage response.^10^ In addition to a wide spectrum of syndromic manifestations, ciliopathies can present with fibrocystic kidney diseases and periportal liver fibrosis due to ductal plate malformation.^11^ Typically, affected individuals with recessive ciliopathies present with major organ disease early in life. In autosomal recessive polycystic kidney disease (ARPKD [MIM: 263200]) for instance, one quarter of affected individuals need renal replacement therapy by the age of 15 years and more than half show signs of portal hypertension by then.^12^ Similarly, end-stage kidney disease due to nephronophthisis usually develops before adulthood.^9^ These early presentations, often with considerable disease burden already manifest prenatally during embryonic development, limit our ability to investigate the initial triggers of organ fibrosis, to study the natural course of disease, and to provide therapeutic windows for potential interventional studies.

*TULP3* (MIM: 604730) encodes a 442-amino acid protein (Tubby-like protein 3), containing an N-terminal intraflagellar transport A (IFT-A)-binding domain and a C-terminal tubby domain with ubiquitous expression.^13^ Acting as an adaptor protein for the ciliary IFT-A machinery, cellular and mouse studies have established a critical role for TULP3 in ciliary trafficking of integral membrane proteins.^14^, ^15^, ^16^ In addition, nuclear roles for TULP3 have been suggested.^17^

Here, we detected 15 individuals from eight unrelated families with bi-allelic variants in *TULP3*. Postnatal disease onset is variable, ranging from childhood to adulthood. The affected individuals we report here are mostly adults, in the 3^rd^ through 7^th^ decades of life, and present with progressive degenerative liver fibrosis with variable fibrocystic kidney disease and hypertrophic cardiomyopathy. Using an adult zebrafish model and cells derived from affected individuals, we propose a model of multisystem fibrosis originating from disrupted ciliary composition and DNA damage.

## Material and methods

Full details of all methods can be found in the supplemental information.

### Ethics statement

Human blood samples for DNA extraction were obtained with written informed consent. This study was approved by the Northeast - Newcastle & North Tyneside 1 Research Ethics Committee (18/NE/350), and the Genomics England 100,000 Genomes Project was approved by the Health Research Authority Research Ethics Committee East of England – Cambridge South (REC Ref 14/EE/1112). For the affected individuals recruited through the Johns Hopkins Baylor-Hopkins Center for Mendelian Genomics project (family 7), this study was approved by the Johns Hopkins and Baylor College of Medicine institutional review boards. All experiments involving zebrafish were approved by the ethical committee (Regierungspräsidium Freiburg, Baden-Württemberg, Germany).

### Study design

All affected individuals and family members involved in this study gave their written informed consent for genetic testing. Full details of study cohorts are provided in the supplemental information. All other human blood samples, human urine-derived renal epithelial cells (URECs), and fibroblast cells were obtained with written informed consent.

### Massively parallel sequencing

In this study, we utilized different approaches based on next-generation sequencing (NGS) technologies and comprehensive bioinformatic analyses described in detail elsewhere.^18^^,^^19^

### Isolation of cells derived from affected individuals

URECs were isolated from urine collected from affected individual II.1 from family 2 and healthy age- and sex-matched controls. Primary fibroblasts were isolated from skin biopsies of affected individual II.2 from family 3 and affected individual II.4 from family 6 and age- and sex-matched controls. Primary cells were maintained as previously described.^19^, ^20^, ^21^

### Zebrafish maintenance and strains

AB/TL wild-type and *Tg(wt1b:EGFP)*^22^ zebrafish (*Danio rerio*) strains were raised under standard conditions at 28°C and staged as previously described.^23^

### Generation of CRISPR-Cas9-induced *tulp3* mutant zebrafish

We used the CHOPCHOP online tool to design efficient guide RNAs (gRNAs) targeting genomic *tulp3* in zebrafish. We injected an exon 5 targeting *tulp3*-gRNA mRNA and *cas9* mRNA to generate a *tulp3* mutant allele. Sanger sequencing confirmed a 5 bp deletion in exon 5 of *tulp3* leading to a frameshift and subsequently to a premature stop codon. Homozygous *tulp3* mutant zebrafish (*tulp3 m/m*) were generated by crossing heterozygous *tulp3* (*tulp3 m/+*) zebrafish. Wild-type zebrafish (*tulp3* +/+) from this cross were raised as clutchmate controls. Maternal-zygotic *tulp3* mutants (MZ*tulp3*) were obtained from incrosses of homozygous zebrafish.

### Immunofluorescence

For cilia imaging, we seeded URECs and fibroblasts derived from affected individuals on coverslips, grew them to 90% confluence, and serum starved them to induce ciliation. Cells were then fixed and incubated in target primary and secondary antibodies solutions.

### Mass spectroscopy

To determine potential interaction partners of TULP3, we used tandem affinity purification combined with mass spectrometric protein identification. Interaction networks were generated by analysis of a list of identified interaction partners with the STRING Protein-Protein Interaction Networks Functional Enrichment Analysis online tool.^24^

### Co-immunoprecipitation of TULP3 and SIRT1

We co-transfected human TULP3 and SIRT1 in FLAG- and V5-tagged pcDNA6 vectors, respectively, into HEK293T cells by using calcium phosphate transfection. We then purified cell lysates by using agarose beads conjugated to FLAG- or V5-tag and performed immunoblot analyses by using antibodies against the reciprocal tag.

### DNA damage response assay

We assessed DNA damage response (DDR) in affected individual’s URECs (family 2 [II.1]) and fibroblasts (family 3 [II.2]) by using immunofluorescence imaging with the DDR marker γH2AX (Cell Signaling and Abcam). Nuclear staining intensity and punctae positive for γH2AX were then quantified and compared in age-, sex-, and passage-matched-control- and affected-individual-derived cells.

### RNA sequencing analysis

RNA sequencing was performed by FASTERIS SA, NGS services. With the QIAGEN RNeasy extraction kit, total RNA was isolated from affected individual (family 3 [II.2]) and age- and sex-matched control fibroblasts following 72 h incubation in DMEM (0.1% FBS) with three biological repeats for each condition.

### Statistical analysis

All data represent results from one of at least three independent experiments, which showed consistent results. Data were analyzed by Student’s t test (two-sided, unpaired) and error bars represent the standard error of the mean (SEM) unless otherwise stated.

## Results

### Identification of bi-allelic *TULP3* variants in individuals with progressive liver, kidney, and heart disease

**Figure 1:**
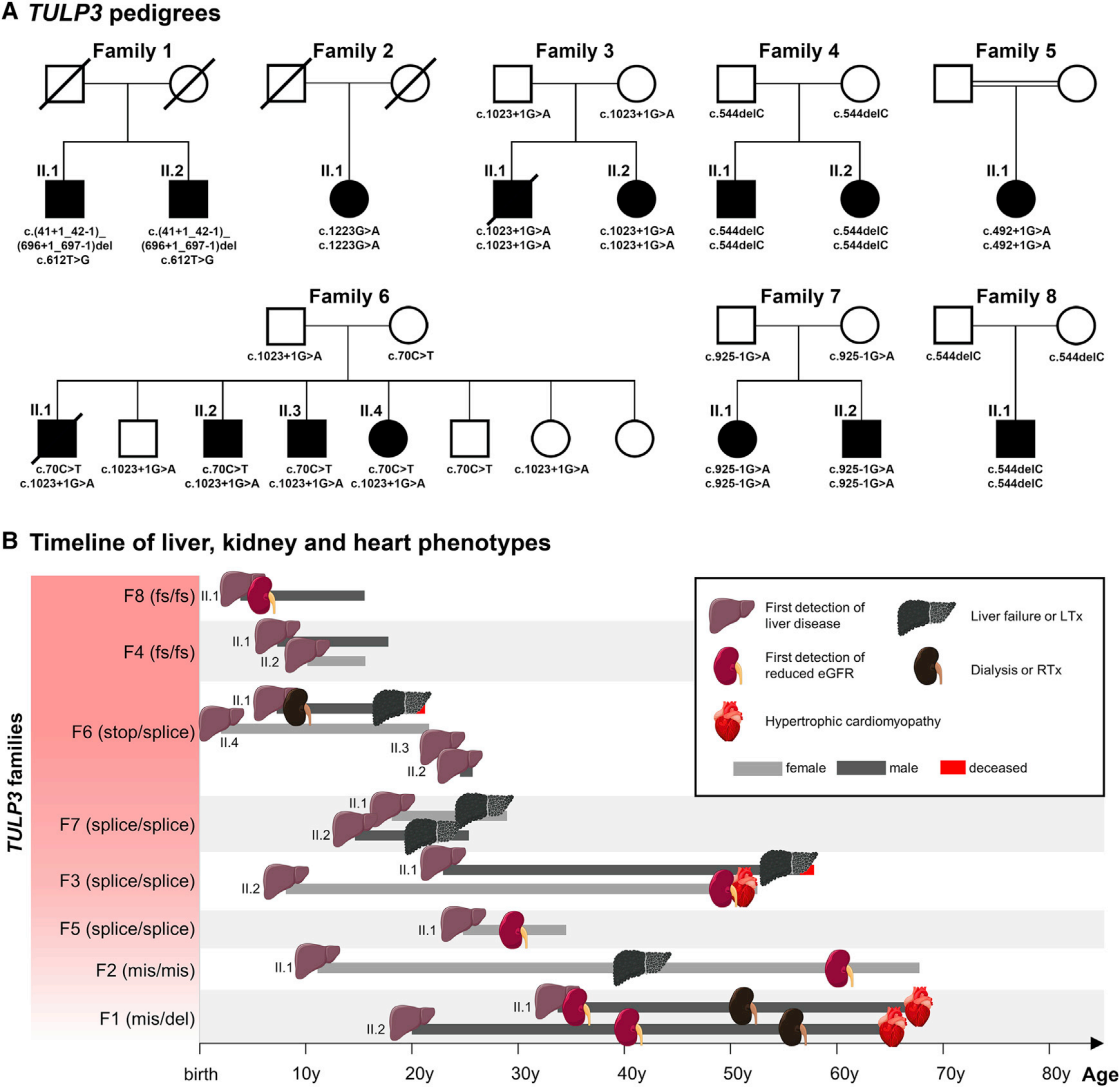
Identification of variants in *TULP3* as a cause of progressive organ fibrosis in 15 affected individuals from eight unrelated families (A) Pedigrees for each of the eight reported families. Different massively parallel sequencing (MPS) approaches and GeneMatcher were used for identification of *TULP3* variants. Affected individuals (black symbols) presented with progressive fibrotic liver disease and variable kidney and heart disease (full details in Table 1). *TULP3* genetic changes are shown below symbols of individuals. Notably, the clinical features segregate with bi-allelic mutations in *TULP3* (homozygous or compound heterozygous), implicating variants in *TULP3* in autosomal recessive progressive fibrotic disease. (B) Graphical timeline showing the age of identification of liver, kidney, and heart phenotypes for all affected individuals (full details in Table 1). All affected individuals presented with complications of liver disease, and initial disease manifestations ranged from 2 to 33 years of age. Signs of chronic kidney disease were predominantly observed starting at the 2^nd^ decade. End-stage kidney disease was observed in three affected individuals at the age of 7, 51, and 55 years (family 6 [II.1] and family 1 [II.1 and II.2], respectively). Three affected individuals were affected by hypertrophic non-obstructive cardiomyopathy (HNCM) in their 6^th^ and 7^th^ decades of life (family 3 [II.2] and family 1 [II.1 and II.2], respectively). eGFR, estimated glomerular filtration rate; LTx, liver transplantation; RTx, renal transplantation; y, years.

**Figure 2:**
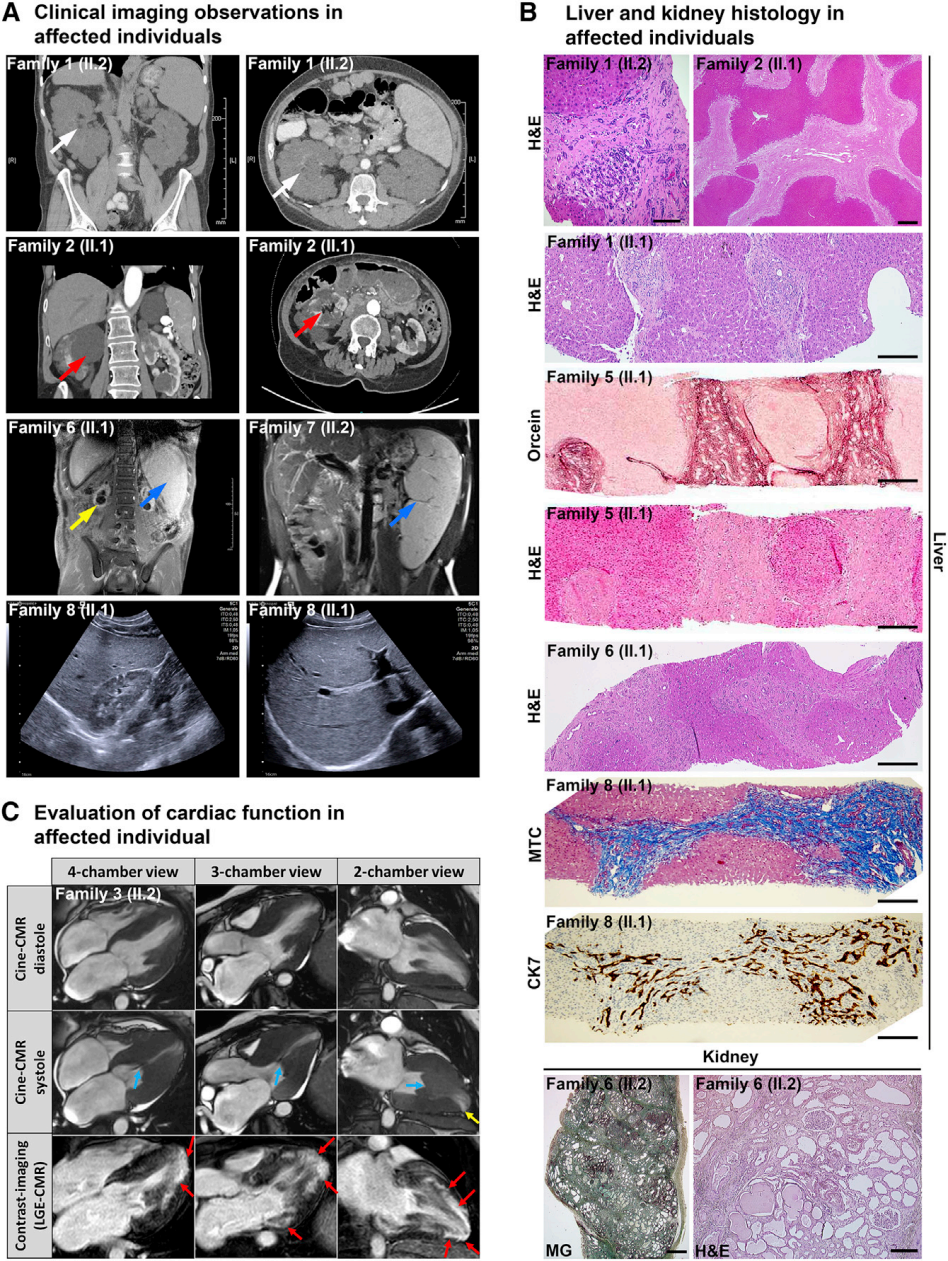
Clinical imaging and histopathological evaluation of affected individuals (A) Imaging data from affected individuals. A computed tomography (CT) of an individual from family 1 (II.2) showing small renal cysts (white arrow). CT image of family 2 (II.1) showing large renal cysts (red arrow); note: transplant liver. Magnetic resonance imaging (MRI) scan from an individual from family 6 (II.1) shows splenomegaly (blue arrow) and end-stage atrophic fibrocystic kidneys (yellow arrow). MRI of affected individuals from family 7 (II.2) showing splenomegaly (blue arrow). Abdominal ultrasound pictures from family 8 (II.1) show renal microcysts with increased cortical echogenicity and hepatomegaly with increased tissue echogenicity. (B) Histopathology phenotypes observed in selected affected individuals. Family 1 (II.2), histological analyses of liver (H&E staining) showing hepatic fibrosis-dependent expansion of portal spaces and only little inflammatory infiltrate (scale bar, 100 μm). Family 2 (II.1) and family 1 (II.1), histological analyses of liver (H&E staining) compatible with hepatic cirrhosis (2 [II.1]: scale bar, 500 μm; 1 [II.1]: scale bar, 200 μm). Family 5 (II.1), orcein and H&E stains highlighting broad fibrous septa crossing the biopsy (scale bar, 200 μm). Family 6 (II.1), fibrosis pattern compatible with either biliary type cirrhosis or congenital hepatic fibrosis-like pattern but without ductal plate malformation, with only minimal portal inflammatory infiltrate and a moderate unspecific ductular reaction (scale bar, 200 μm). Family 8 (II.1), architectural disturbance of the hepatic parenchyma due to portal bridging fibrosis as assessed by Masson`s trichome staining (MTC). CK7 (cytokeratin 7) staining shows proliferation of dysmorphic bile ductules (scale bar, 200 μm). Family 6 (II.2), kidney sections: MG (May-Gruenwald) staining (scale bar, 1,000 μm), and H&E staining (scale bar, 100 μm) showing renal fibrosis and kidney cysts. (C) Cardiovascular magnetic resonance (CMR) images of the affected individuals from family 3 (II.2) that were acquired at the age of 53 years. The upper panel shows cine-CMR images obtained in diastole. Middle panel illustrates corresponding systolic cine-images. CMR reveals a severe, concentric, septally pronounced pattern of LV hypertrophy with additional intraventricular obstruction due to a kissing-wall phenomenon (blue arrows) and subsequent apical wall thinning with regional akinesia (yellow arrow). Corresponding late-gadolinium-enhancement (LGE) images are illustrated in the lower panels. Hyperintense areas are indicative of myocardial fibrosis (red arrows). A progressive and extensive non-ischemic pattern of LGE was depicted not only at the right ventricular (RV) insertion points (basal anteroseptal and inferoseptal LV wall) but also in the mid- to apical LV free wall and whole LV apex. This extensive and peculiar pattern of myocardial fibrosis cannot be explained by classical hypertrophic cardiomyopathy (HCM) but in contrast indicates a systemic disease with cardiac involvement.

We used whole-exome sequencing (WES) or targeted exome sequencing (TES) including >600 genes with a known or hypothesized association to cystic kidney disease and other ciliopathies and kidney disorders (see supplemental methods)^19^ to detect pathogenic variants in a cohort of individuals affected with fibrocystic liver/kidney disease or other ciliopathy-associated diseases. We first investigated a German family from our fibrocystic liver/kidney disease cohort with two affected brothers aged 65 and 68 years, both presenting with slowly progressing liver disease, initiating at around 20 years of age with elevated liver enzymes together with clinical features of portal hypertension, fibrocystic kidney disease leading to end-stage kidney disease (at 55 and 51 years, respectively), and hypertrophic non-obstructive cardiomyopathy (Figures 1A and 1B, Figures 2A and 2B, and Table 1). Using TES including copy number variant (CNV) analysis, we identified bi-allelic genetic variants in *TULP3* (GenBank: NM_003324.5) in both individuals (family 1: c.(41+1_42−1)_(696+1_697−1)del/c.612T>G [p.Cys204Trp]) (Figures S1 and S2). No further variants of relevance were detected. Notably, *Tulp3* knockout (KO) in mice leads to embryonic lethality and typical signs of aberrant ciliary signaling.^13^^,^^25^^,^^26^ Interestingly, increased circulating bilirubin levels have been reported in *Tulp3* heterozygous null mice^27^ and recent *Tulp3* hypomorphic mutant or nephron-specific KO mice displayed cystic kidney disease.^14^^,^^16^ To find additional cases, we then (1) applied WES or TES in 5,124 genetically unsolved individuals from the same cohort of individuals with fibrocystic liver/kidney disease or other ciliopathy associated diseases,^19^ (2) screened whole-genomic sequencing (WGS) data from the Genomics England 100,000 Genomes Project^28^ (∼35,000 probands with rare diseases, including ∼1,500 probands with cystic kidney disease or unexplained kidney failure, ∼800 with hypertrophic cardiomyopathy, and ∼250 probands recruited under ductal plate malformation and/or cirrhosis), and (3) utilized GeneMatcher.^29^ Altogether, we identified thirteen additional affected individuals from seven unrelated families (three from the fibrocystic liver/kidney disease cohort, two from Genomics England, and two through GeneMatcher) carrying bi-allelic predicted deleterious variants in *TULP3* that segregated with the disease phenotype and without detecting other variants of interest (family 2, c.1223G>A [p.Arg408His]; family 3, c.1023+1G>A; family 4, c.544delC [p.Leu182TrpfsTer4]; family 5, c.492+1G>A; family 6, c.1023+1G>A/c.70C>T [p.Arg24Ter]; family 7, c.925−1G>A; family 8, c.544delC [p.Leu182TrpfsTer4]) (Figure 1A, Figures S1 and S2, Table 1, and Table S1). Overall, we identified eight different genetic variants, among them six predicted high impact (multiexon deletion, nonsense, frameshifting, and canonical splice-affecting) and two missense (Table S1). The missense variants affect highly conserved residues within the functionally important tubby domain of TULP3 (Figures S1 and S3). Two splicing variants (c.925−1G>A and c.1023+1G>A) are predicted to lead to in-frame exon 9 skipping (99 bp) and removal of the 8^th^ beta sheet of TULP3; this prediction was verified for family 3 (c.1023+1G>A) (Figures S3E–S3H).

**Table 1:** Clinical observations in affected individuals with bi-allelic *TULP3* variants

**Family**	**Genomic variation (GRCh38)**	***TULP3*****nucleotide/amino acid change (GenBank:****NM_003324.5**)	**Affected individual ID, origin, sex, age**	**Liver phenotype**	**Kidney phenotype**	**Cardiac phenotype**	**Malignancy**	**Other clinical features**
1	allele 1 12:(3000155_3018694)_(3040407_3042583)	c.(41+1_42−1)_(696+1_697−1)del	II.1, German, M, 68 years	HSM, inhomogeneous liver parenchyma, portal HTN, elevated liver enzymes (22 years), cirrhosis	cystic kidneys (detected 33 years), HD (51 years), RTx (52 years)	cardiac MRI: hypertrophic non-obstructive cardiomyopathy (68 years)	basal cell carcinoma forehead, squamous cell carcinoma right eyebrow (59 years)	–
	allele 2 12:2931156:T:G rs547315819	c.612T>G (p.Cys204Trp)	II.2, German, M, 65 years	elevated liver enzymes (20 years), HSM, inhomogeneous liver parenchyma, portal HTN, variceal banding, hepatic encephalopathy, cirrhosis, awaiting LTx (since age 57 years)	cystic kidneys (detected at 43 years), HD (55 years), on waiting list for RTx (since age 57 years)	cardiac MRI: hypertrophic non-obstructive cardiomyopathy (65 years)	NSCLC adenocarcinoma (62 years)	arterial HTN (54 years), chronic pancreatitis, multiple pancreatic cysts
2	allele 1/2 12:2939338:G:A rs761172007	c.1223G>A (p.Arg408His)	II.1, British, F, 68 years	cholestasis/jaundice, portal HTN, GI bleeding (11 years), portovenous shunt (12 years), biliary cirrhosis, LTx (41 years)	multiple cortical and small medullary renal cysts, enlarged kidneys, eGFR: 40	TTE normal (41 years)	no	primary infertility, arterial HTN splenic artery aneurysm
3	allele 1/2 12:2937730:G:A rs202037575	c.1023+1G>A	II.1, German, M, 57 years (deceased)	elevated liver enzymes (25 years), portal HTN (31 years), esophageal bleeding (33 years), portacaval shunt, hepatic encephalopathy, and death due to liver failure (57 years)	not known	not examined	no	–
			II.2, German, F, 53 years	elevated liver enzymes in childhood, HSM, thrombocytopenia, bridging fibrosis (38 years)	normal-sized kidneys with hyperechogenic parenchyma and reduced CMD (51 years), eGFR: 52 (53 years)	cardiac MRI: hypertrophic non-obstructive cardiomyopathy (53 years); biopsy—moderate chronic myocardial damage, diffuse interstitial fibrosis of myocardium, and degeneration of myocardial cells	no	–
4	allele 1/2 12:2931087:C: rs924744512	c.544delC (p.Leu182TrpfsTer4)	II.1, Macedonian, M, 18 years	elevated liver enzymes in childhood, cholestatic hepatopathy, HSM, portal HTN with hypersplenism, variceal banding, increased elastography values (14 years)	no	TTE and ECG normal (14 years)	no	–
			II.2, Macedonian, F, 16 years	abdominal pain in childhood, HSM, portal HTN with hypersplenism (+pancytopenia), variceal banding (12 years)	no	TTE and ECG normal (13 years)	no	–
5	allele 1/2 12:2930346:G:A rs145289428	c.492+1G>A	II.1, Pakistani, F, 34 years	cholestasis, gestational pruritus (26 years), portal HTN, bridging fibrosis	eGFR: 50, normal kidney USS	TTE normal (34 years)	no	arterial HTN, obesity, Bell’s palsy, labyrinthitis, chronic tonsillitis
6	allele 1 12:2937730:G:A rs202037575	c.1023+1G>A	II.1, German, M, 21 years (deceased)	elevated liver enzymes in childhood. HSM with inhomogeneous liver parenchyma, ascites, cirrhosis, LTx (21 years), deceased age 21 years (post Tx complications)	normal-sized kidneys with hyperechogenic parenchyma and reduced CMD, HD (7 years), 1^st^ RTx (8 years), 2^nd^ RTx (15 years)	TTE normal (21 years)	no	–
	allele 2 12:2909557:C:T rs201665307	c.70C>T (p.Arg24Ter)	II.2, German, M, 26 years	elevated liver enzymes (19 years), HSM, cirrhosis	enlarged hyperechogenic kidneys, eGFR > 90; biopsy—diffuse interstitial fibrosis, corticomedullary scarring, tubular dilatations	TTE normal (26 years)	no	–
			II.3, German, M, 24 years	elevated liver enzymes (20 years), HSM, bridging fibrosis	enlarged hyperechogenic kidneys, eGFR > 90	TTE normal (24 years)	no	–
			II.4, German, F, 22 years	elevated liver enzymes in infancy, HSM, esophageal variceal bleeding, TIPS (20 years), bridging fibrosis with architectural distortion, awaiting LTx	renal parenchymal hyperechogenicity, reduced CMD, right kidney small isolated 2 mm cyst (21years)	TTE normal (20 years)	no	–
7	allele 1/2 12:2937630:G:A rs761012512	c.925−1G>A	II.1, Northern European, F, 29 years	elevated liver enzymes in early adulthood, portal HTN with esophageal varices (25 years), cirrhosis with mild lobular and portal inflammation, LTx (27 years)	non-enlarged cystic kidneys, eGFR > 90	TTE normal (27 years)	no	acute pancreatitis (19 years)
			II.2, Northern European, M, 26 years	elevated liver enzymes in childhood, decompensated portal HTN (18 years), TIPS (20 years), cirrhosis, LTx (21 years)	enlarged kidneys, right kidney 2 cysts (15 mm), eGFR > 60	TTE normal (20 years)	hepatocellular carcinoma (20 years)	–
8	allele 1/2 12:2931087:C: rs924744512	c.544delC (p.Leu182TrpfsTer4)	II.1, Italian, M, 16 years	cholestasis and jaundice (onset 4 years), portal HTN, bridging fibrosis with architectural distortion	non-enlarged kidneys with cortical and medullary microcysts with increased cortical echogenicity, eGFR: 86	TTE normal (16 years)	no	–

Affected individuals present with fibrotic liver features (bridging fibrosis, cirrhosis), variable fibrocystic kidney disease, and hypertrophic non-obstructive cardiomyopathy in older affected individuals (6^th^ to 7^th^ decade). Abbreviations: CHF, congenital hepatic fibrosis; CMD, corticomedullary differentiation; ECG, electrocardiogram; eGFR, estimated GFR (CKD-EPI formula) mL/min/1.73 m^2^; F, female; GI, gastrointestinal; HSM, hepatosplenomegaly; HTN, hypertension; LTx, liver transplant; M, male; NSCLC, non-small cell lung cancer; RTx, renal transplant; TIPS, transjugular intrahepatic portosystemic shunt; TTE, transthoracic echocardiogram; USS, ultrasound scan.

### Clinical and histopathological features of affected individuals with *TULP3* variants

In all affected individuals, the disease initially manifested with complications of liver disease (Figure 1B). Abnormal liver enzyme tests were the earliest sign of disease; in particular, biochemical markers of cholestasis were increased (Table 1). Younger affected individuals presented with cholestatic jaundice or abdominal pain, and one affected individual presented with gastro-intestinal bleeding secondary to portal hypertension. Liver disease progressed during childhood, and the earliest instances of liver transplantation were in the 3^rd^ decade (families 6 and 7). The affected individuals in family 1 are the oldest surviving individuals without liver transplantation (65 and 68 years); one of the brothers is currently on the liver transplantation waiting list (Table 1). Computed tomography imaging demonstrates liver enlargement with inhomogeneous parenchyma and secondary signs of portal hypertension in affected individuals, but no liver cysts were seen, distinguishing this disease from other cystic kidney and liver disorders (Figure 2A).

Histopathological stains on liver biopsy or liver explants from nine different individuals from five unrelated families were reviewed (Figure 2B and Table S2). Histological evaluation of liver biopsy samples showed a paucicellular portal fibrosis, which was bridging with or without architectural distortion or established cirrhosis. Most cases revealed only a minimal portal inflammatory infiltrate and a moderate unspecific ductular reaction. The explant liver in family 2 (II.1) demonstrated biliary type fibrosis without evidence of an interrupted circular arrangement of ducts, which would be characteristic for ductal plate malformation in the setting of congenital hepatic fibrosis. The explant liver from family 6 (II.1) showed a non-specific cirrhotic pattern. Liver cysts or von-Meyenburg complexes were not detected in the samples available for histological evaluation. In conclusion, none of the samples from affected individuals showed the typical histological pattern of congenital hepatic fibrosis that has been described in association with ARPKD^30^ (Table S2).

Kidney involvement was detected in all but one family (family 4, which has the youngest affected individuals of our cohort at 16 and 18 years). Kidney disease was heterogeneous and chronic kidney disease was usually detected later in life (3^rd^–6^th^ decade) (Figure 1B and Table 1). The most common ultrasonographic presentation was hyperechogenic kidneys with reduced corticomedullary differentiation or multiple kidney cysts (Figure 2A). A kidney biopsy (family 6 [II.2]) showed widespread interstitial fibrosis with tubular dilatations (Figure 2B and Table 1).

Three adult individuals from two unrelated families presented with morphological signs of hypertrophic non-obstructive cardiomyopathy (HNCM) detected in their 6^th^ or 7^th^ decade of life. A cardiac biopsy of affected individual II.2 from family 3 showed evidence of diffuse interstitial fibrosis of the myocardium and degeneration of myocardial cells (Table 1). In the same individual, MRI of the heart revealed left ventricular hypertrophy and systemic myocardial fibrosis (Figure 2C). We screened the other, mostly younger affected individuals with transthoracic echocardiogram, none of whom showed features of HNCM (Table 1).

### Inactivation of zebrafish *tulp3* causes adult liver and kidney disease

**Figure 3:**
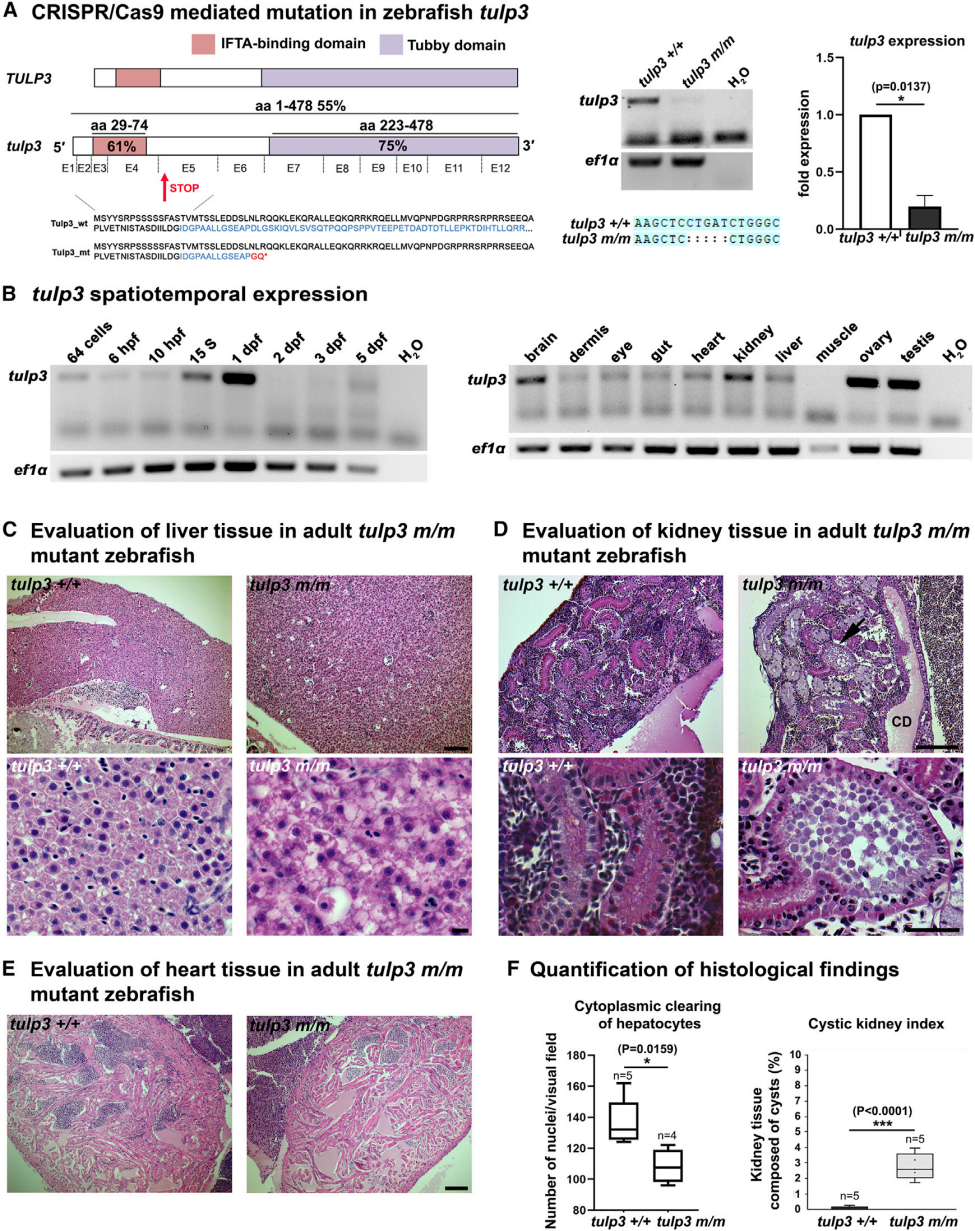
Inactivation of zebrafish *tulp3* causes adult liver and kidney disease (A) Left: schematic showing conservation between human TULP3 and zebrafish Tulp3. Amino acid sequences aligned with the Clustal Omega MView tool. Tulp3 shows 54.5% overall homology with TULP3 particularly within the IFT-A-binding (red) and Tubby (purple) domains (61% and 75%, respectively). The lower part of the figure shows mapping of the exons onto Tulp3 protein structure and shows the position of CRISPR-Cas9-mediated deletion in exon 5 leading to an early stop codon, p.Asp106Glyfs2Ter. Right: Semiquantitative RT-PCR and qPCR revealed a strong reduction in *tulp3* mRNA expression in MZ*tulp3* mutant embryos compared to the respective control indicating a functional *tulp3* null mutation. Sanger sequencing confirmed the 5 bp deletion in exon 5. Error bar represents SEM; ∗p < 0.05 (one-sample t test). (B) Semiquantitative RT-PCR analysis of *tulp3* expression during development (left) and in isolated adult zebrafish tissues (right). *tulp3* is expressed in various adult tissues, including liver, kidney, and heart; *ef1α* was used as housekeeping gene. Hpf, h post fertilization; dpf, days post fertilization; 15 S, 15-somites stage. (C) Histological analyses of liver samples (H&E stain) isolated from adult (18 months) *tulp3 +/+* wild-type and *tulp3 m/m* mutant zebrafish clutchmates. Liver sections of *tulp3 m/m* zebrafish show cytoplasmic clearing of hepatocytes indicating steatosis (scale bar upper panel, 100 μm; scale bar lower panel, 10 μm). (D) Histological analyses of kidney samples (H&E stain) isolated from adult (18 months) *tulp3 +/+* wild-type and *tulp3 m/m* mutant zebrafish clutchmates. Kidney sections of *tulp3 m/m* zebrafish show mild cysts (black arrow), observed in both proximal and distal kidney tubules (scale bar upper panel, 50 μm; scale bar lower panel, 10 μm). CD: collecting duct. (E) Histological analyses of heart samples (H&E stain) isolated from adult (18 months) *tulp3 +/+* wild-type and *tulp3 m/m* mutant zebrafish clutchmates (scale bar, 100 μm). Microtome sections of the adult zebrafish ventricle. We did not note any fibrotic events or cellular changes in the hearts of these animals at 18 months. (F) Boxplots for liver and kidney phenotypes observed in *tulp3 m/m* adult zebrafish. For indirect quantification of cytoplasmic clearing, the nuclei of hepatocytes in visual fields of *tulp3 m/m* compared to *tulp3 +/+* clutchmates were analyzed, showing a significant reduction in nuclei in *tulp3 m/m* zebrafish. ^∗^p = 0.0159 (two-tailed, unpaired Student’s t test). An increased cystic index score was observed in *tulp3 m/m* zebrafish kidney compared to *tulp3 +/+* clutchmates (n = 5). ^∗∗∗^p < 0.001 (two-tailed, unpaired Student’s t test).

We investigated the functions of TULP3 by using the zebrafish as a vertebrate model organism. Zebrafish Tulp3 is closely related to its human counterpart (Figure 3A). Semiquantitative RT-PCR analysis performed on zebrafish embryos indicated *tulp3* expression throughout embryogenesis with peak levels during the first 24 h post fertilization (hpf) and at 5 days post fertilization (dpf). These data are consistent with publicly available zebrafish developmental RNA sequencing data (e.g., EBI Expression Atlas^31^) showing *tulp3* expression at all studied time points (zygote to 5 dpf) with highest relative levels at late gastrulation/beginning somitogenesis (6–10 hpf) and later stages (4–5 dpf), suggesting continued expression of *tulp3* after embryonic development. Analysis on a series of adult zebrafish tissues indicated highest expression (relative to housekeeping gene expression) in the gonads, the brain, as well as the kidney, liver, and heart (Figure 3B).

We generated a KO model of zebrafish *tulp3* through a CRISPR-Cas9-mediated 5 bp deletion that induces a stop codon in exon 5 of *tulp3* (Figure 3A). Semiquantitative RT-PCR and qPCR on cDNA from maternal-zygotic (MZ)*tulp3* mutants and control clutchmates confirmed the 5 bp deletion and premature stop codon and demonstrated significantly reduced *tulp3* expression that most likely results from nonsense-mediated decay (Figure 3A). Given the late onset of clinical features in reported affected individuals, we evaluated the effect of Tulp3 loss of function in adult zebrafish, which survive to adulthood, in contrast to comparable murine models of TULP3 loss of function.^26^

Due to the prominent liver, kidney, and heart phenotypes observed in affected individuals harboring deleterious *TULP3* variants, we analyzed tissue sections from adult (18 months old) homozygous *tulp3* zebrafish mutant (*tulp3 m/m*) liver, kidney, and heart. We then compared these to control zebrafish (*tulp3 +/+*) derived from the same incross (clutchmates). In the livers of adult *tulp3 m/m,* we observed significant cytoplasmic clearing of the hepatocytes indicating steatosis (Figures 3C and 3F). Adult *tulp3 m/m* also develop a mild cystic kidney disease with cysts in both proximal and distal tubules (Figure 3D). Cystic index scoring reveals a mild but consistent cystic kidney phenotype in these animals compared to *tulp3 +/+* animals (Figure 3F). Evaluation of heart tissue from adult *tulp3 m/m* zebrafish mutants found no aberrant morphological features, and histological examination found no indication of fibrosis or underlying cellular disruptions (Figure 3E).

### Disrupted ciliary cargo composition in cells derived from affected individuals

To characterize the molecular and cellular consequences of *TULP3* mutations in affected individuals, we obtained primary, non-transformed human URECs (family 2 [II.1]) as well as skin fibroblasts (family 3 [II.2] and family 6 [II.4]) and age- and sex-matched controls from non-affected individuals.

**Figure 4:**
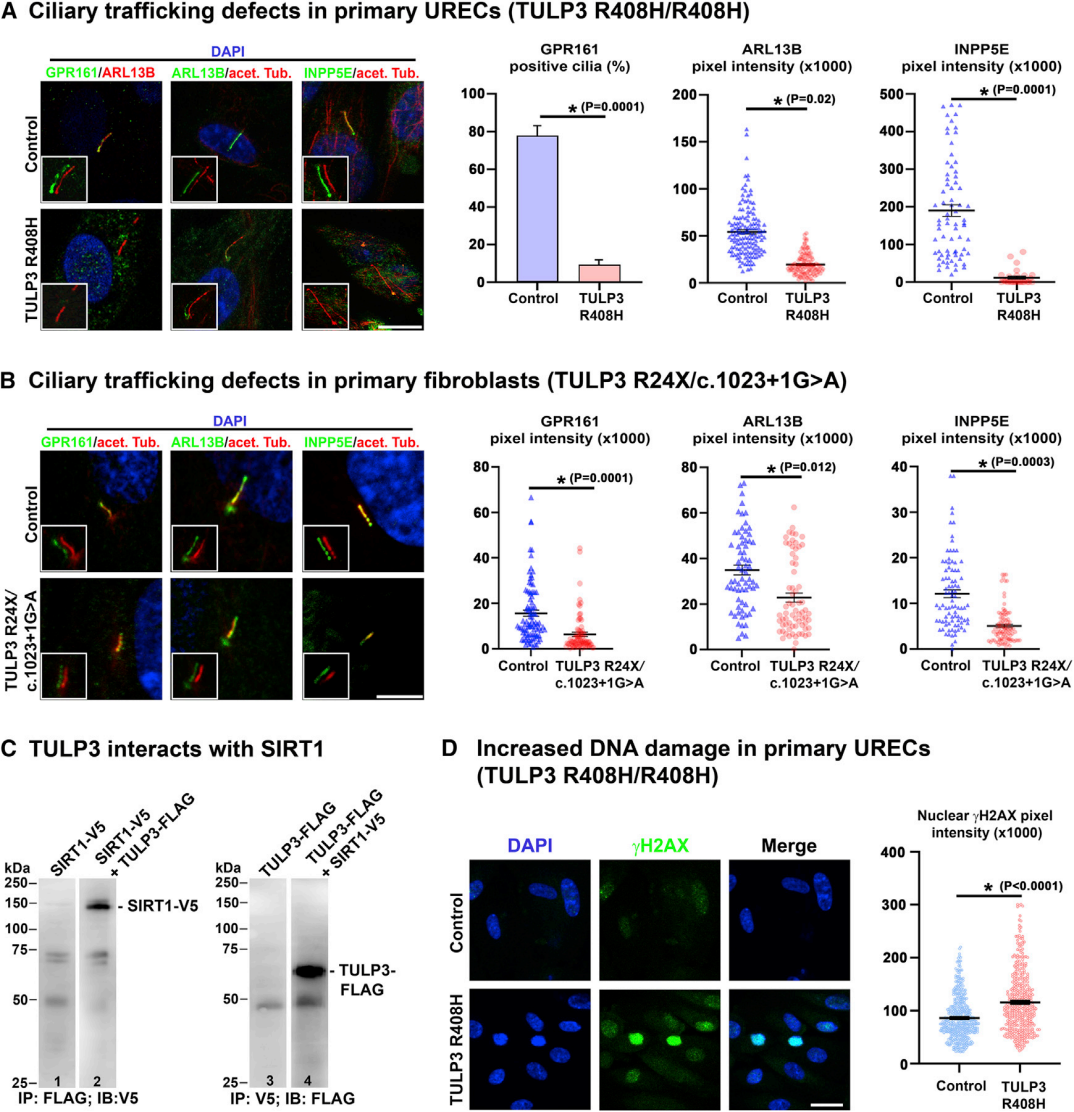
*TULP3*-affected individuals’ cells show defects in ciliary composition and increased DNA damage (A) Representative confocal micrographs assessing the effect of *TULP3* mutations on ciliary composition in urine-derived renal epithelial cells (URECs) (family 2 [II.1], c.1223G>A [p.Arg408His]) and compared to age- and sex-matched control cells. Serum-starved cells were stained with antibodies against acetylated tubulin, GPR161, ARL13B, and INPP5E. Cell nuclei were counterstained with DAPI. Affected-individual-derived URECs showed significantly reduced ciliary localization of GPR161, ARL13B, and INPP5E. Left panel: staining of control and affected individual (family 2 [II.1)]) URECs (scale bar, 5 μm). Right panel: corresponding quantification of GPR161-positive cilia and ciliary signal intensities of ARL13B and INPP5E. (B) Representative confocal micrographs assessing the effect of *TULP3* mutations on ciliary composition in fibroblasts derived from an affected individual (family 6 [II.4], c.1023+1G>A/c.70C>T [p.Arg24Ter]). Compared to age- and sex-matched control cells affected-individual-derived fibroblasts showed significantly reduced ciliary localization of GPR161, ARL13B, and INPP5E (scale bar, 5 μm). Right panel: corresponding quantification of ciliary signal intensity levels. For (A) and (B): ^∗^p < 0.05; error bars show SEM (two-tailed, unpaired Student’s t test performed on the means of three independent experiments). (C) Interaction of human TULP3 with SIRT1. FLAG-tagged full-length human TULP3 was co-transfected with V5-tagged full-length SIRT1 in HEK293T cells. SIRT1 was detected in TULP3 precipitates (FLAG-M2 beads for immunoprecipitation [IP], anti-V5 for immunoblotting [IB]), and correspondingly TULP3 was detected in SIRT1 precipitates (V5 beads for IP, anti-FLAG for IB); kDa, kilodalton. (D) Increased DNA damage response (DDR) in affected-individual-derived URECs (family 2 [II.1]). γH2AX was used as an immunocytochemical marker of DDR and intensity of nuclear signal was compared in the affected individual and sex- and age-matched control URECs at the same passage number (scale bar, 20 μm). A significant increase in γH2AX signal was detected in affected-individual-derived URECs compared to control cells. ^∗^p < 0.05; error bars represent SEM (two-tailed, unpaired Student’s t test on three independent experiments).

Most of the detected genetic variants were predicted to have a high (disruptive) impact on protein function (Table S1). Furthermore, immunofluorescence analysis revealed near-complete loss of ciliary TULP3 localization in URECs from the family 2 affected individual with a homozygous missense variant (Figure S4A). We investigated the consequences of *TULP3* mutations on ciliary composition by assessing the levels of TULP3 cargo proteins GPR161, ARL13B, and INPP5E in URECs and fibroblast cells derived from affected individuals. In primary cells from family 2 (II.1) and family 6 (II.4), we observed a strong reduction in ciliary GPR161, a negative regulator of sonic hedgehog (SHH) signaling.^32^ ARL13B intensities were reduced in these affected individuals’ cells and INPP5E was nearly undetectable, an observation possibly secondary to reduced ARL13B levels (Figures 4A and 4B). Notably, localization assays in fibroblasts from family 3 (II.2) (c.1023+1G>A) showed less severe localization defects; only GPR161 displayed defective trafficking, consistent with the in-frame splicing defect caused by this mutation (Figures S3E–S3H and Figures S4B–S4E). These results are in line with previous experiments mostly obtained in *Tulp3*-deficient models^14^, ^15^, ^16^ and suggest TULP3 loss of function as a shared disease mechanism in our affected individuals. In conclusion, using URECs and fibroblast cells derived from affected individuals, we demonstrate a functional impact of the identified genetic variants in *TULP3* resulting in disruption of ciliary composition, including proteins previously associated with human ciliopathies (MIM: 213300, 612291).^33^

### TULP3 interacts with DNA damage repair protein and key fibrosis modulator SIRT1

To identify potential TULP3 interaction partners, we performed mass spectroscopy on tandem affinity purified HEK293T whole-cell lysate. The TULP3 interaction network suggests an association with several core DDR elements, including DDB1 and TP53 (Figure S5). Among the identified protein interactions was the recently reported TULP3 interaction partner SIRT1,^34^ a class III histone deacetylase that has broad reaching roles as a regulator of transcription and DDR by mediating deacetylation of TP53 and several histones.^35^, ^36^, ^37^ We confirmed the interaction between TULP3 and SIRT1 through co-immunoprecipitation in HEK293T cells (Figure 4C). TULP3 has been shown to locate both to the plasma membrane/primary cilium as well as to the nucleus,^13^^,^^17^ where SIRT1 is predominantly expressed.^37^ Given the link between TP53 and DDR, we next assessed levels of γH2AX, a marker of DNA damage, in URECs (family 2 [II.1]) and fibroblasts (family 3 [II.2]) derived from affected individuals and found significant increase in γH2AX nuclear staining intensity and punctae, suggesting increased DNA damage in these cells (Figure 4D and Figure S4F). Notably, SIRT1 has also been identified as a modulator of organ fibrosis most likely linked to its role as modulator of TGF-β signaling.^38^^,^^39^

### Profibrotic WNT-signaling-, TGF-β-signaling-, and cardiomyopathy-related gene expression is significantly increased in *TULP3* cells derived from affected individuals

**Figure 5:**
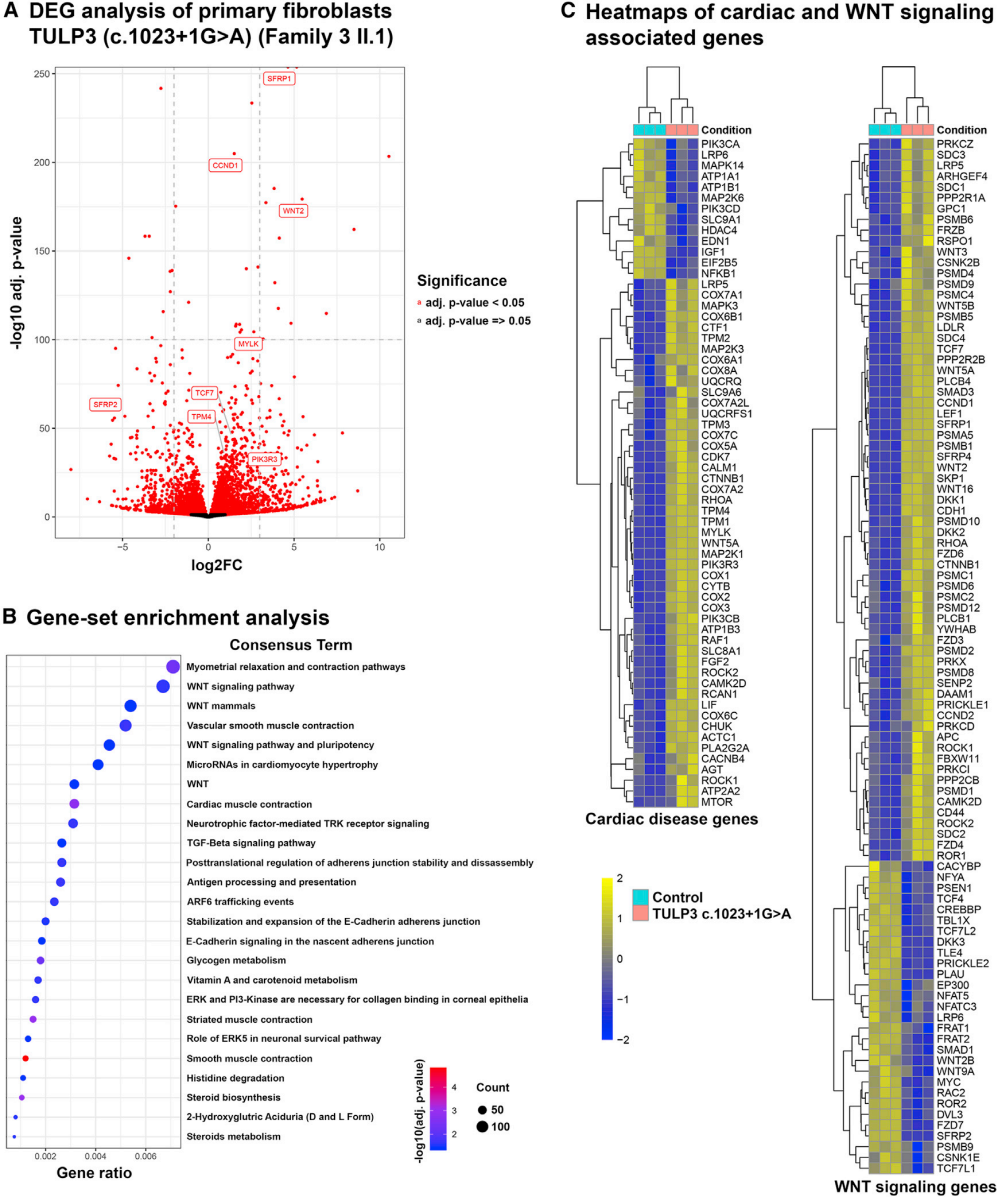
Increased levels of WNT-signaling-, TGF-β-signaling-, and cardiomyopathy-associated genes in *TULP3*-affected individual’s cells (A) RNA sequencing results performed in healthy control and fibroblasts derived from affected individual (family 3 [II.2]). Differentially regulated genes (DEGs) were identified by the gene set analysis method GAGE (generally applicable gene-set enrichment). The most dysregulated genes associated with WNT signaling or cardiac disease are labeled. (B) Enrichment analysis of signaling pathways. Pathways were considered significant with adjusted p values (Benjamini-Hochberg) p < 0.05. Among these pathways, significantly dysregulated genes associated with WNT signaling, TGF-β signaling, and cardiac muscle contraction/microRNAs in cardiomyocyte hypertrophy were identified. (C) Gene expression heatmaps for differentially regulated genes from the indicated GSEA terms “cardiac muscle contraction/microRNAs in cardiomyocyte hypertrophy” (left) and WNT signaling (right). Each column represents an individual sample from control or affected-individual-derived cells.

To gain further insight into the pathophysiology underlying *TULP3*-related disease phenotypes, we performed RNA sequencing in fibroblast cells from one of our affected individuals harboring the homozygous canonical *TULP3* splicing variant c.1023+1G>A (family 3 [II.2]). In accordance with the clinical phenotype, gene clusters for hypertrophic cardiomyopathy and WNT and TGF-β signaling were significantly dysregulated (Figure 5 and Figure S6A). Upregulation of TGF-β effectors *SMAD3* (MIM: 603109) and direct targets of canonical TGF-β signaling *SERPIN1* (MIM: 173360), as well as downregulation of the TGF-β pathway inhibitor *SMAD7* (MIM: 602932), suggest activation of the TGF-β pathway^40^ (Figure S6A). Similarly, upregulation of *LEF1* (MIM: 152245) and *TCF7* (MIM: 189908), principle WNT pathway effectors, indicates activation of the WNT signaling pathway^41^ (Figure 5C). Notably, the WNT-signaling-associated genes include members of both the canonical β-catenin as well as the non-canonical planar cell polarity (PCP) pathways (Figure 5C). Upregulation of TGF-β signaling is noteworthy and could be secondary to disrupted interaction between TULP3 and SIRT1 in affected-individuals-derived cells (Figure S6A). The SHH pathway is notably absent from our transcriptional analysis. Targeted evaluation of SHH pathway components in family 3 (II.2) fibroblasts by qPCR confirmed no deregulation of key SHH pathway components in this affected individual’s cells (Figure S6B). We observed only few downregulated signaling processes; significant gene reduction was mainly associated with functions in cell cycle, ribosome, and circadian rhythm (Figure S6C).

## Discussion

We describe bi-allelic variants in *TULP3* in 15 individuals from eight unrelated families and establish by further *in vitro* and *in vivo* experiments variants in *TULP3* as a monogenic cause for a clinically distinct disorder of progressive degenerative liver fibrosis with variable fibrocystic kidney disease and hypertrophic cardiomyopathy. Postnatal disease onset was variable, ranging from childhood to adulthood. Therefore, our study highlights the increasing importance of broad genetic sequencing for the discovery of autosomal recessively inherited disorders, even among adults affected with a primarily fibrotic disease affecting the liver, kidney, and heart. We hypothesize that early detection of individuals at high risk for progression to end-stage liver and kidney disease could lead to improved clinical management, focusing on preservation of renal function and screening for complications of cirrhosis, such as esophageal varices and hepatocellular carcinoma, which occurred in several young adults in our study. The progressing course of disease caused by *TULP3* variants demonstrates the importance of periodic liver, kidney, and heart screening tests to delay organ dysfunction through early therapeutic actions.

Organ fibrosis is clinically and genetically heterogeneous and not well understood. So far, only a few genes representing monogenic causes for liver fibrosis are known.^42^ Defects in genes resulting in alterations of the primary cilium are linked to hepatorenal fibrocystic disease with ductal plate malformation and periportal fibrosis.^30^ Notably, liver biopsies obtained from several affected individuals in this study are different from typical ductal plate malformation, suggesting a distinct clinical and histopathological phenotype.

We observe considerable variability in disease onset and expression in the affected individuals reported here. We did not identify additional, predicted functional variants in genes potentially interacting with *TULP3* or the identified signaling pathways. Notably, we do observe an apparent association between the age of disease onset and the predicted effect of the *TULP3* variants, i.e., the single individual carrying two missense variants had a milder disease course compared to individuals with bi-allelic frameshifting variants (Figure 1B). Similarly, family 3 that is bi-allelic for the likely hypomorph frame-preserving splice variant c.1023+1G>A seemed to present a milder clinical course than family 6 where this hypomorph variant was associated with an early nonsense variant. Given the small numbers, further studies are required to better delineate a possible genotype-phenotype correlation.

Among the six families with homozygous variants in *TULP3*, only one was knowingly consanguineous. The identified *TULP3* variants are all rare, and none of them are detected at the homozygote state in gnomAD. Although none of the remaining five families presented obvious intrafamilial relationships, we noticed the recurrence of two variants: c.544delC in two Mediterranean families (Italian and Macedonian) and c.1023+1G>A in two German pedigrees. This observation may suggest a shared ancestor in these families. In line with a potential common distant parental ancestor, we also detected a stretch of homozygosity around *TULP3* in the proband from family 2, albeit shorter compared with the proband from the consanguineous family 5 (∼0.3 Mb versus 6 Mb, data not shown).

We modeled TULP3 loss of function in *tulp3* KO zebrafish. In adult Tulp3-deficient zebrafish, we observed fibrocystic disease including liver fibrosis and cystic kidney disease, which mirrors the clinical presentation of affected individuals with deleterious *TULP3* mutations. *TULP3*-affected individuals develop hypertrophic cardiomyopathy that appears to be age related. Evaluation of heart tissue from adult *tulp3 m/m* zebrafish mutants did not reveal any underlying pathology. Given the 3–5 year lifespan of zebrafish,^43^ at 18 months of age it is likely that these animals may not have been old enough to develop the respective age-related cardiac phenotypes.

RNA sequencing demonstrates significant dysregulation of profibrotic pathways in line with the clinical course of our affected individuals with *TULP3* variants. In contrast to the described roles of TULP3 as a negative regulator of SHH,^32^ transcription-level expression data from cells derived from affected individuals indicate significant upregulation of key effectors of both WNT and canonical TGF-β signaling pathways, suggesting that both WNT and canonical TGF-β signaling are activated in these affected individuals. Notably, the primary cilium appears to be a signaling hub for canonical TGF-β.^44^ Furthermore, murine models of cardiac fibrosis require primary cilia to propagate TGF-β-mediated fibrosis.^45^

SHH, WNT, and TGF-β signaling are strongly interrelated mediators of fibrosis with well-established functions at the primary cilium. The SHH pathway has been shown to direct differentiation of myofibroblasts through interaction with both TGF-β and WNT signaling pathways.^46^^,^^47^ TULP3 functions as an adaptor for a subset of membrane-bound proteins destined for the cilium and therefore is in a prime position to provide fine control of integrated signaling pathways. Notably, while TULP3 is a well-established regulator of the SHH pathway, we do not observe any deregulation of the SHH pathway in our transcriptional RNA-sequencing analysis or through qPCR analysis of SHH components. The absence of SHH signaling is in line with the clinical presentation where typical manifestations such as laterality defects or polydactyly were not observed.

**Figure 6:**
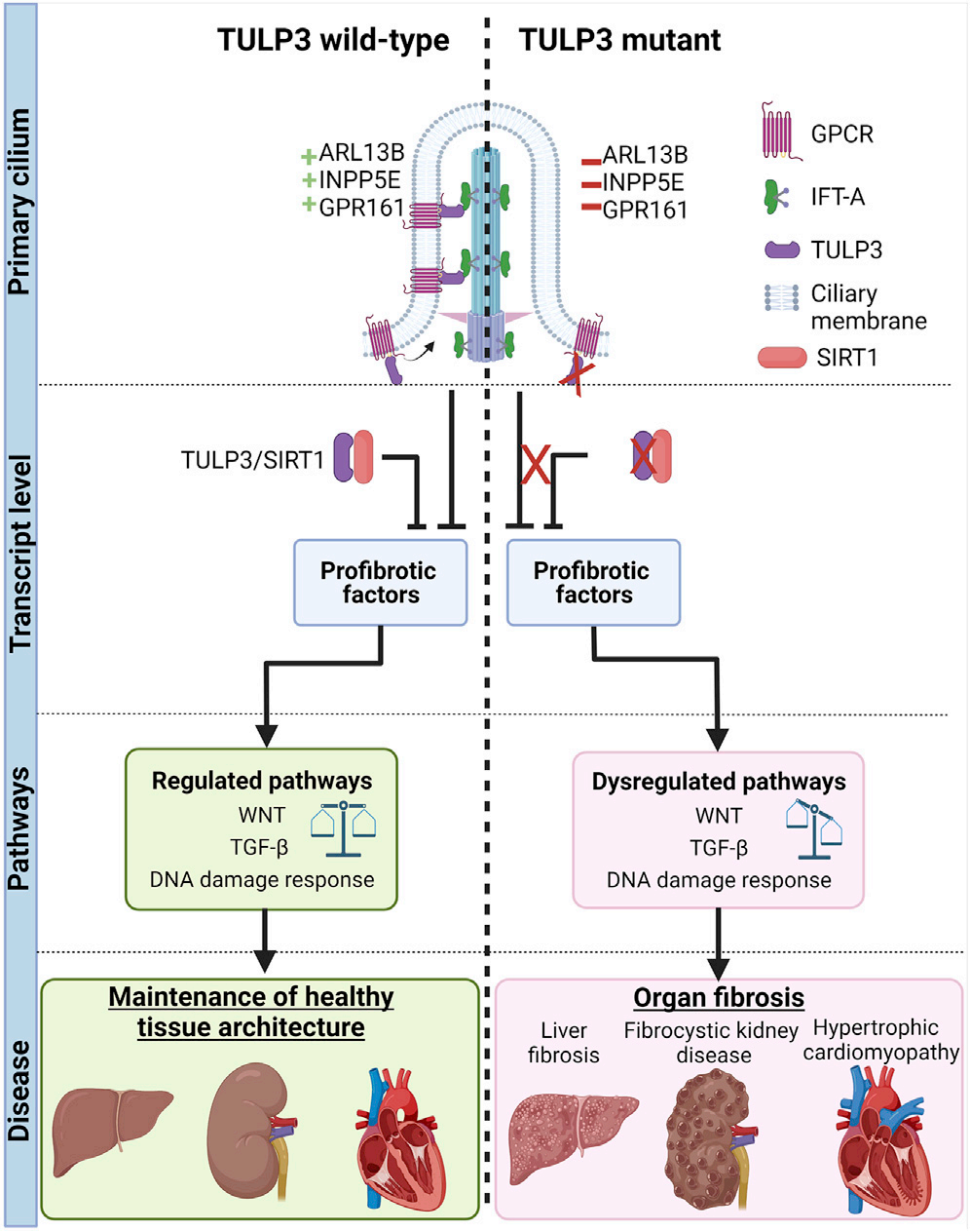
Converging pathomechanisms of fibrosis in *TULP3*-affected individuals A schematic of the hypothesized disease mechanism of fibrosis in *TULP3*-affected individuals. Shown are cartoons of the primary cilium with axoneme, intraflagellar transport machinery and membrane bound receptors and ciliary proteins, transcriptional changes, affected pathways, and clinical outcomes. On the left is the *TULP3* wild-type healthy state versus *TULP3* mutant disease state (right), separated by the vertical dotted line. At the cilium, *TULP3* mutations results in defective trafficking of ARL13B, INPP5E, and GPR161 (and likely other GPCRs). Disrupted ciliary composition leads to a loss of regulatory signals from the cilium, causing dysregulated profibrotic WNT and TGF-β signaling pathways, either directly or indirectly. Green crosses represent correctly localized receptors and proteins. Red crosses represent loss of receptors or proteins from the cilium. We demonstrate that TULP3 interacts directly with SIRT1, a key regulator of fibrosis. We hypothesize that disruptive mutations in TULP3 lead to reduced SIRT1 modulation of profibrotic signaling pathways through a yet unknown mechanism. We propose that TULP3 is a key regulator of fibrosis that functions at multiple levels. Disruption of these regulatory mechanisms converge and results in chronic activation of profibrotic signaling cascades leading to progressive fibrosis in *TULP3*-affected individuals. Black arrows represent activation, bar headed lines represent inhibition, and red Xs represent loss of elements from the network. Created with https://biorender.com/.

TULP3 also displays nuclear localization and translocates from the cilium to the nucleus upon GPCR activation.^17^ While the functions of TULP3 at the nucleus are unknown, we identified potential interaction with DDR pathway proteins and observe increased DDR in our cells derived from affected individuals. Notably, aberrant DDR has been linked to progressive fibrosis in affected individuals with hypomorphic *CEP164* (MIM: 614848) mutations.^10^ Additionally, we confirm a direct interaction between TULP3 and SIRT1, a well-established regulator of TGF-β-mediated organ fibrosis.^38^^,^^39^ The role that TULP3 plays in either of these pathways is incompletely elucidated and will require further investigation (Figure 6).

Altogether, we describe compelling clinical and experimental data to substantiate variants in *TULP3* as a monogenic cause of progressive degenerative liver fibrosis with variable fibrocystic kidney disease and hypertrophic cardiomyopathy. This disease is a pathophysiologically distinct entity that shares commonalities with other ciliopathies. Expanding our understanding of fibrogenesis and the contribution of ciliopathy-associated genes to diverse phenotypes may open new therapeutic approaches for the treatment of progressive organ fibrosis.

## Data and code availability

Primary data from the 100,000 Genomes Project, which are held in a secure research environment, are available to registered users. See https://www.genomicsengland.co.uk/research/academic for further information.

## Web resources

CHOPCHOP target sites for CRISPR-Cas9 mutagenesis, https://chopchop.cbu.uib.no/
ClinVar, https://www.ncbi.nlm.nih.gov/clinvar
Clustal omega, https://www.ebi.ac.uk/Tools/msa/clustalo/
EBI-Expression Atlas, https://www.ebi.ac.uk/gxa/experiments/E-ERAD-475
Ensembl, https://www.ensembl.org/index.html
Ensembl VEP, https://www.ensembl.org/info/docs/tools/vep/index.html
Genomics England 100,000 Genomes Project, https://www.genomicsengland.co.uk/
GnomAD v2.1.1, https://gnomad.broadinstitute.org/
HGMD, http://www.hgmd.cf.ac.uk/ac/index.php
ImageJ, https://imagej.nih.gov/ij/
OMIM, https://omim.org/
ProteinPaint, https://pecan.stjude.cloud/proteinpaint
STRING, https://string-db.org/
The International Mouse Phenotyping Consortium webpage, https://www.mousephenotype.org/
Varsome, https://varsome.com/

## Supplemental information

Document S1. Figures S1–S6 and supplemental methods

Table S1. Molecular genetic findings in *TULP3*, allele frequency and *in silico* predictions

Table S2. Centrally reviewed liver histopathology assessment in affected individuals with *TULP3* variants

Document S2. Article plus supplemental information
