# Supplementary material for: Progressive liver, kidney, and heart degeneration in children and adults affected by TULP3 mutations
Source: Am J Hum Genet. 2022 Apr 8;109(5):928–43. doi: 10.1016/j.ajhg.2022.03.015 (PMC9118107; doi:10.1016/j.ajhg.2022.03.015)
Supplement: Document S1. Figures S1–S6 and supplemental methods [file mmc1.pdf]

The American Journal of Human Genetics, Volume *109*

Supplemental information

Progressive liver, kidney, and heart degeneration in children and adults affected by *TULP3* mutations

John Devane, Elisabeth Ott, Eric G. Olinger, Daniel Epting, Eva Decker, Anja Friedrich, Nadine Bachmann, Gina Renschler, Tobias Eisenberger, Andrea Briem-Richter, Enke Freya Grabhorn, Laura Powell, Ian J. Wilson, Sarah J. Rice, Colin G. Miles, Katrina Wood, Genomics England Research Consortium, Palak Trivedi, Gideon Hirschfield, Andrea Pietrobattista, Elizabeth Wohler, Anya Mezina, Nara Sobreira, Emanuele Agolini, Giuseppe Maggiore, Mareike Dahmer-Heath, Ali Yilmaz, Melanie Boerries, Patrick Metzger, Christoph Schell, Inga Grünewald, Martin Konrad, Jens König, Bernhard Schlevogt, John A. Sayer, and Carsten Bergmann

Supplemental Figures

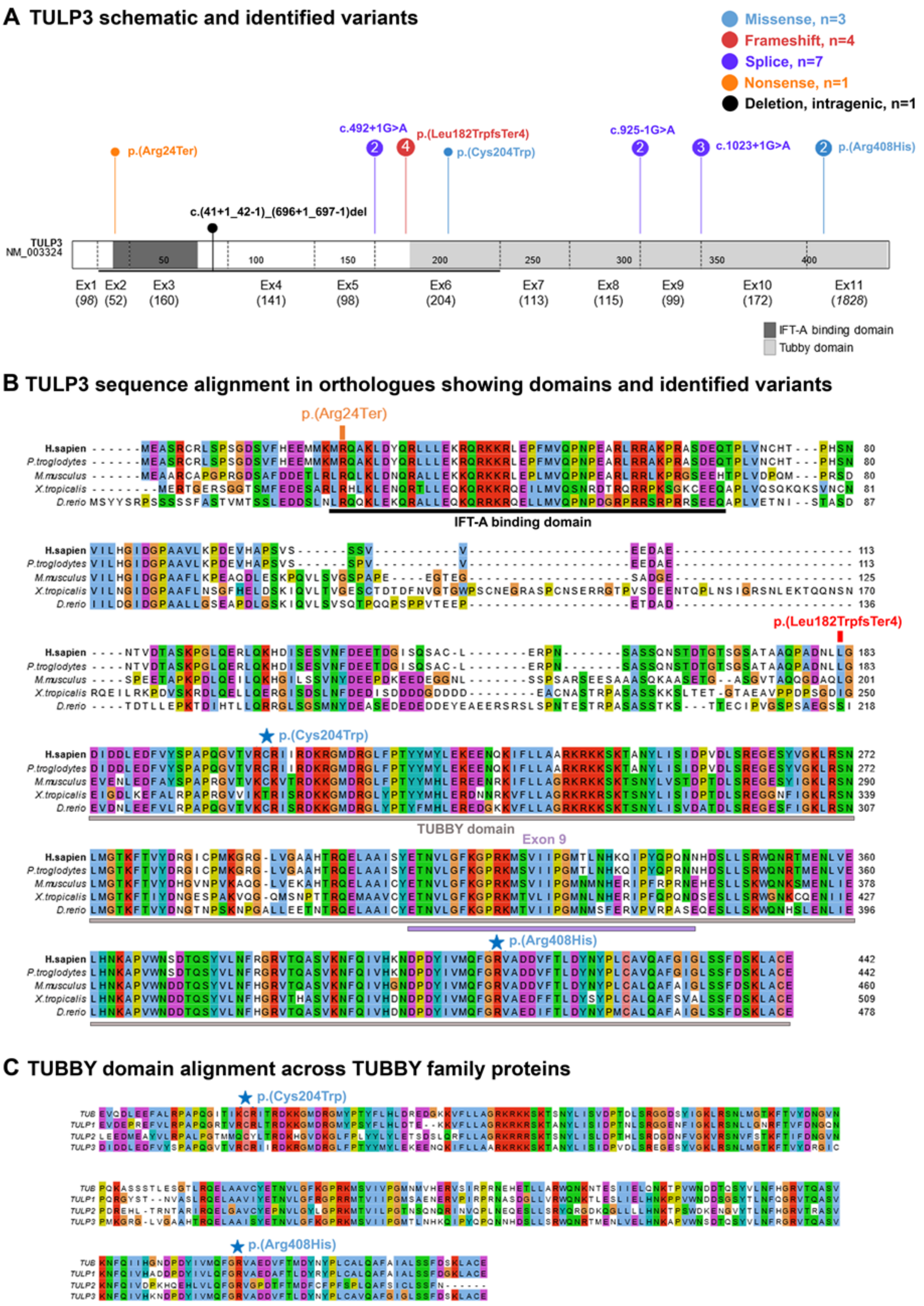

Figure S1. Overview of identified *TULP3* variants, conservation across species and functional domains.

Panel A is a schematic of TULP3, exons are shown bounded by dotted lines and nucleotide length (including untranslated regions) is indicated. The IFT-A binding domain and the tubby domain are shown in black or grey shades, as indicated. Each of the eight variants we identified is mapped into its respective amino acid position or exon boundary for intronic variants. The variant type is annotated, and color coded. Disc number reflects the mutant allele count in family probands. **Panel B** represents amino acid sequence alignments for canonical *TULP3* transcripts for the indicated species. Functional domains as well as exon 9 (targeted by 2 distinct predicted splice-affecting variants) are depicted with colored lines. The nonsense, missense and frameshifting variants are indicated above the alignments. **Panel C** shows tubby domain amino acid alignments for different members of the Tubby protein family and highlights the identified missense variants. Sequences correspond to human canonical transcripts. Sequences were retrieved via Ensembl and alignments performed with Clustal omega.

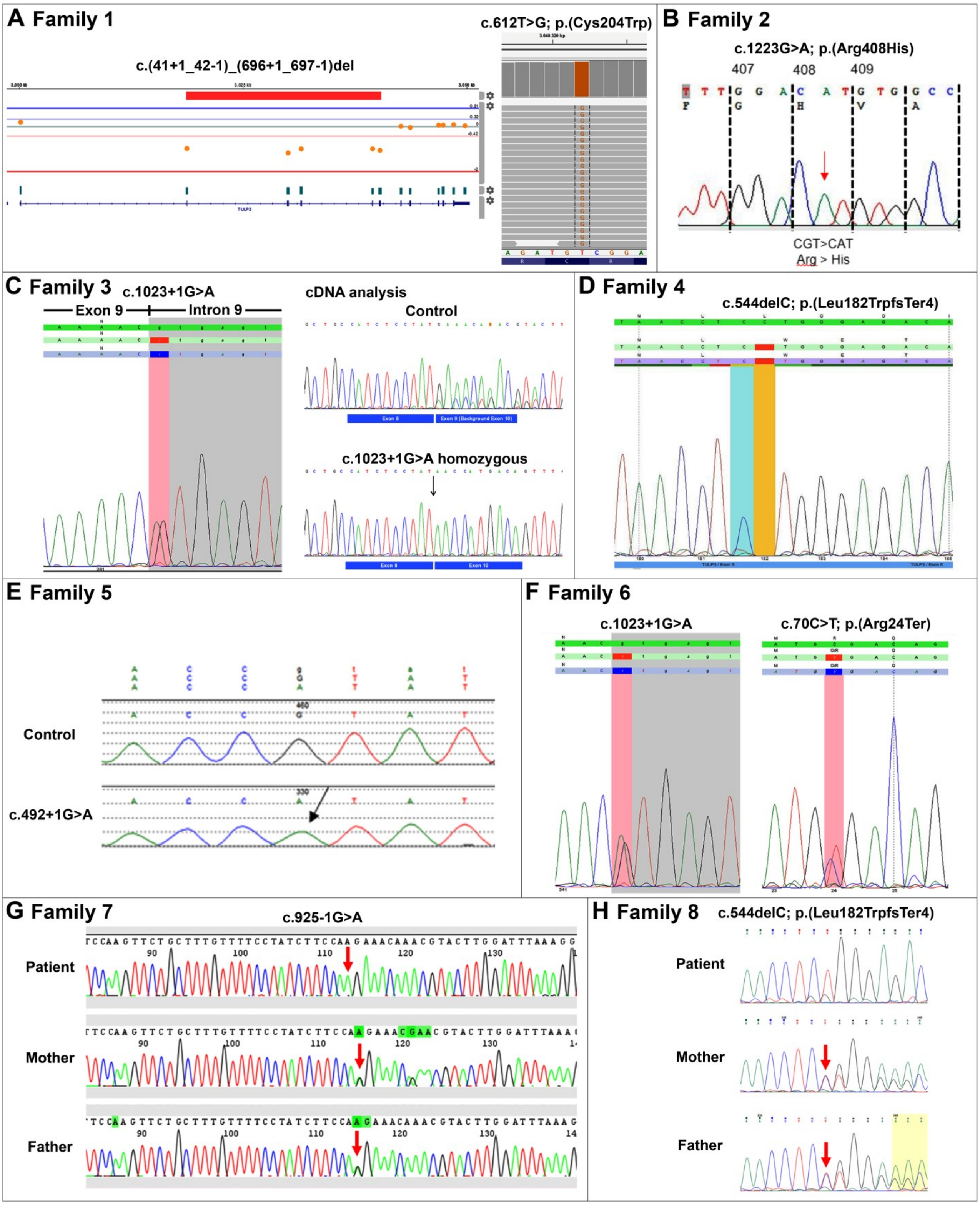

Figure S2. NGS analysis and/or chromatograms for the probands of each family.

Panel A shows the read count obtained from NGS sequencing showing heterozygous c.(41+1_42-1)_(696+1_697-1) deletion in *TULP3* in which exons 2-6 are deleted, and an Integrative Genomics View (IGV) of BAM file read alignments for mutation c.612T>G;

p.(Cys204Trp). **Panel B** shows a Sanger chromatogram of the homozygous c.1223G>A; p.(Arg408His) change observed in Family 2. **Panel C** shows electropherograms of healthy parental and affected proband samples of Family 3. Affected individuals harbor a homozygous c.1023+1G>A change that results in an in-frame skipping of exon 9 and parental samples are heterozygous for this allele. **Panel D** shows an electropherogram of the homozygous mutation c.544delC*;* p.(Leu182TrpfsTer4) detected in Family 4. **Panel E** shows a Sanger chromatogram of the homozygous mutation c.492+1G>A observed in family 5. The black arrow denotes the position of the change. **Panel F** shows two electropherograms from an affected individual from Family 6 displaying the compound heterozygous mutations c.1023+1G>A and c.70C>T; p.(Arg24Ter). **Panel G** shows a Sanger chromatogram of the homozygous mutation c.925-1G>A observed in Family 7. **Panel H** shows a Sanger chromatogram from an affected individual from Family 8 with the homozygous mutation c.544delC; p.(Leu182TrpfsTer4) and his heterozygous parents.

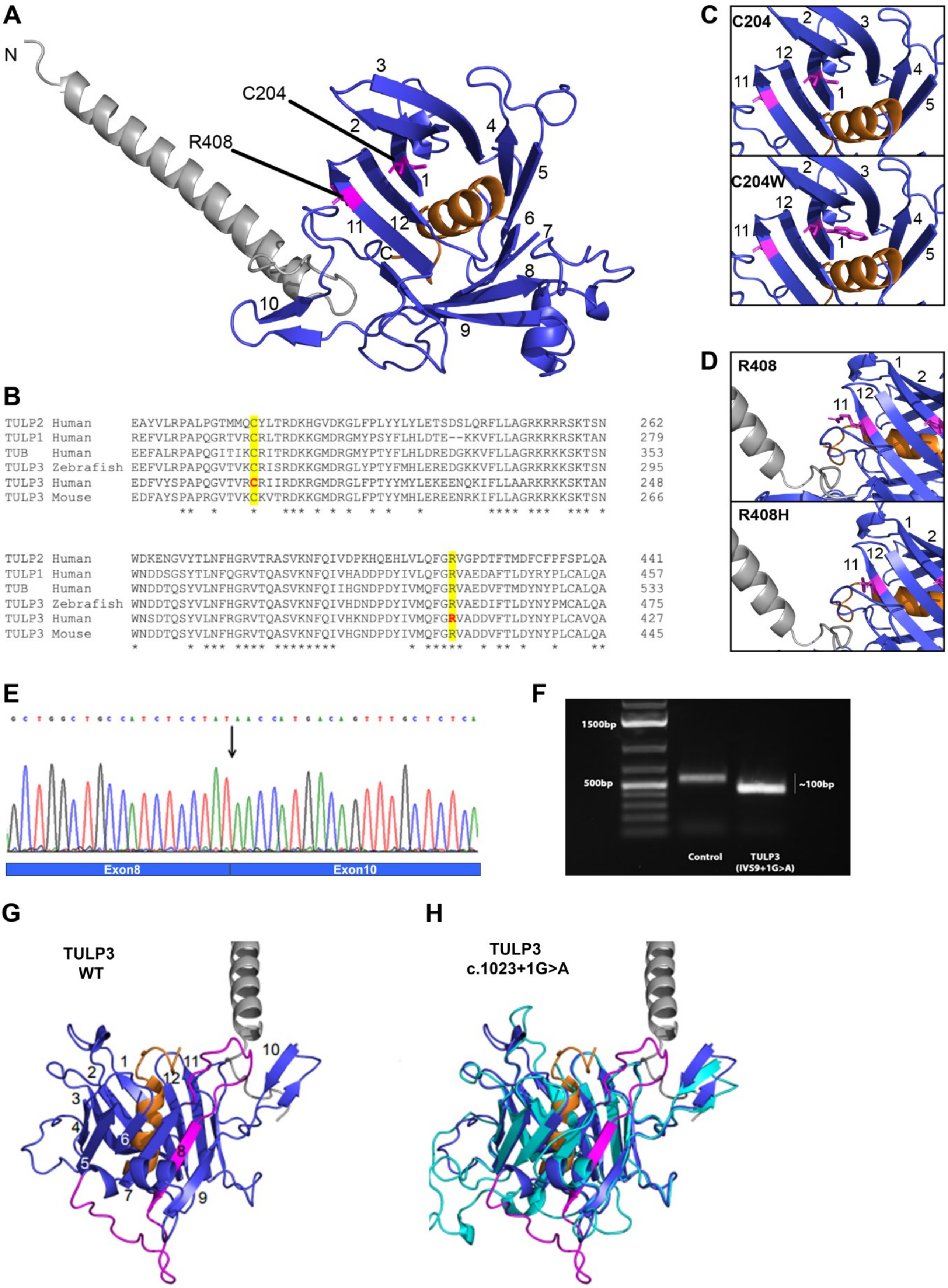

A
N
C204
R408
3
2
4
1
5
11
12
6
7
C
8
10
9
C
C204
C204W
2
3
12
4
11
1
5
D
R408
R408H
1
2
11
12
B
TULP2 Human EAYVLRPALPGTMMQCYLTRDKHGVDKGLFPLYYLYLETSDSLQRFLLAGRKRRRSKTSN 262
TULP1 Human REFVLRPAPQGRTVRCRLTRDKKGMDRGMYPSYFLHLDTE--KKVFLLAGRKRKRSKTAN 279
TUB Human EEFALRPAPQGITIKCRITRDKKGMDRGMYPTYFLHLDREDGKKVFLLAGRKRKKSKTSN 353
TULP3 Zebrafish EEFVLRPAPQGVTVKCRISRDKKGMDRGLYPTYFMHLEREDGKKVFLLAGRKRKKSKTSN 295
TULP3 Human EDFVYSPAPQGVTVRCRIIRDKRGMDRGLFPTYYMYLEKEENQKIFLLAARKRKKSKTAN 248
TULP3 Mouse EDFAYSPAPRGVTVKCKVTRDKKGMDRGLFPTYYMHLEREENRKIFLLAGRKRKKSKTSN 266
** * * *** * * * * * * **** *** *** *
TULP2 Human WDKENGVYTLNFHGRVTRASVKNFQIVDPKHQEHLVLQFGRVGPDTFTMDFCFPFSPLQA 441
TULP1 Human WNDDSGSYTLNFQGRVTQASVKNFQIVHADDPDYIVLQFGRVAEDAFTLDYRYPLCALQA 457
TUB Human WNDDTQSYVLNFHGRVTQASVKNFQIIHGNDPDYIVMQFGRVAEDVFTMDYNYPLCALQA 533
TULP3 Zebrafish WNDDTQSYVLNFHGRVTQASVKNFQIVHDNDPDYIVMQFGRVAEDIFTLDYNYPMCALQA 475
TULP3 Human WNSDTQSYVLNFRGRVTQASVKNFQIVHKNDPDYIVMQFGRVADDVFTLDYNYPLCAVQA 427
TULP3 Mouse WNDDTQSYVLNFHGRVTQASVKNFQIVHGNDPDYIVMQFGRVADDVFTLDYNYPLCALQA 445
* * *** **** ******** * ***** * ** * * **
E
Exon8
Exon10
F
1500bp
500bp
~100bp
Control
TULP3
(IVS9+1G>A)
G
TULP3
WT
1
2
3
4
5
6
7
8
9
10
11
12
H
TULP3
c.1023+1G>A

Figure S3. ***In silico*** **modeling of** ***TULP3*** **variants.**

Panel A Predicted model of human TULP3 displaying the regions modelled by AlphaFold with very high confidence (per-residue confidence score (pLDDT) > 90). The N-terminal alpha helix is coloured in grey. The twelve β strands of the tubby domain β barrel are numbered and coloured in blue. The α helix, central to the hydrophobic core of the tubby domain is coloured in orange. **Panel B** Multiple sequence alignment of human TULP proteins (TUB and TULP1-3) and mouse and zebrafish TULP3. The positions of missense mutations C204W and R408H are highlighted in yellow. *, completely conserved residue in all aligned sequences. **Panel C** Upper panel: A rotated and zoomed in view of the C204 residue (magenta) within β sheet 1 of the tubby domain. Lower panel, *in silico* mutagenesis of TULP3 C204 to W204, resulting in the presence of a large hydrophobic tryptophan residue. **Panel D** Upper panel: A rotated and zoomed in view of the R408 residue (magenta) within β sheet 11 of the tubby domain. Lower panel, *in silico* mutagenesis of TULP3 R408 to H408. **Panel E** Results of RT-PCR Sanger sequencing of *TULP3* c.1023+1G>A fibroblasts derived from affected individual aligned against the exons of *TULP3*, showing in frame skipping of *TULP3* exon 9. **Panel F** shows gel electrophoresis comparing the size of cDNA fragments of *TULP3* generated from RNA isolated from control fibroblasts and *TULP3* c.1023+1G>A fibroblasts (RT-PCR). Fragments generated using primers targeted against the 3' half of *TULP3* (including exon 9) resulting in an expected fragment size of 600 bp. We observe about 100 bp reduction in the size of fragments generated from *TULP3* c.1023+1G>A samples. This is consistent with the predicted in-frame skipping of exon 9 in these affected individuals (99 bp). Left is a DNA ladder with 500 bp and 1500 bp annotated (GeneRuler 1kb plus). **Panel G** Predicted model of human TULP3 displaying the regions modelled by AlphaFold with very high confidence (per-residue confidence score (pLDDT) >90). The N-terminal alpha helix is coloured in grey. The twelve β strands of the tubby domain β barrel are numbered and coloured in dark blue. The α helix, central to the hydrophobic core of the tubby domain is coloured in orange. **Panel H** Predicted model of human TULP3 c.1023+1G>A tubby domain barrel created using HHPred based on structural homology to human TUB1 (PDB, 3C5N), coloured in cyan and superposed on the

wild-type structure as shown in panel G (in blue, orange and pink). Strand 8 of the tubby domain β barrel (coloured in pink on the wild-type structure) is missing from the mutant structure.

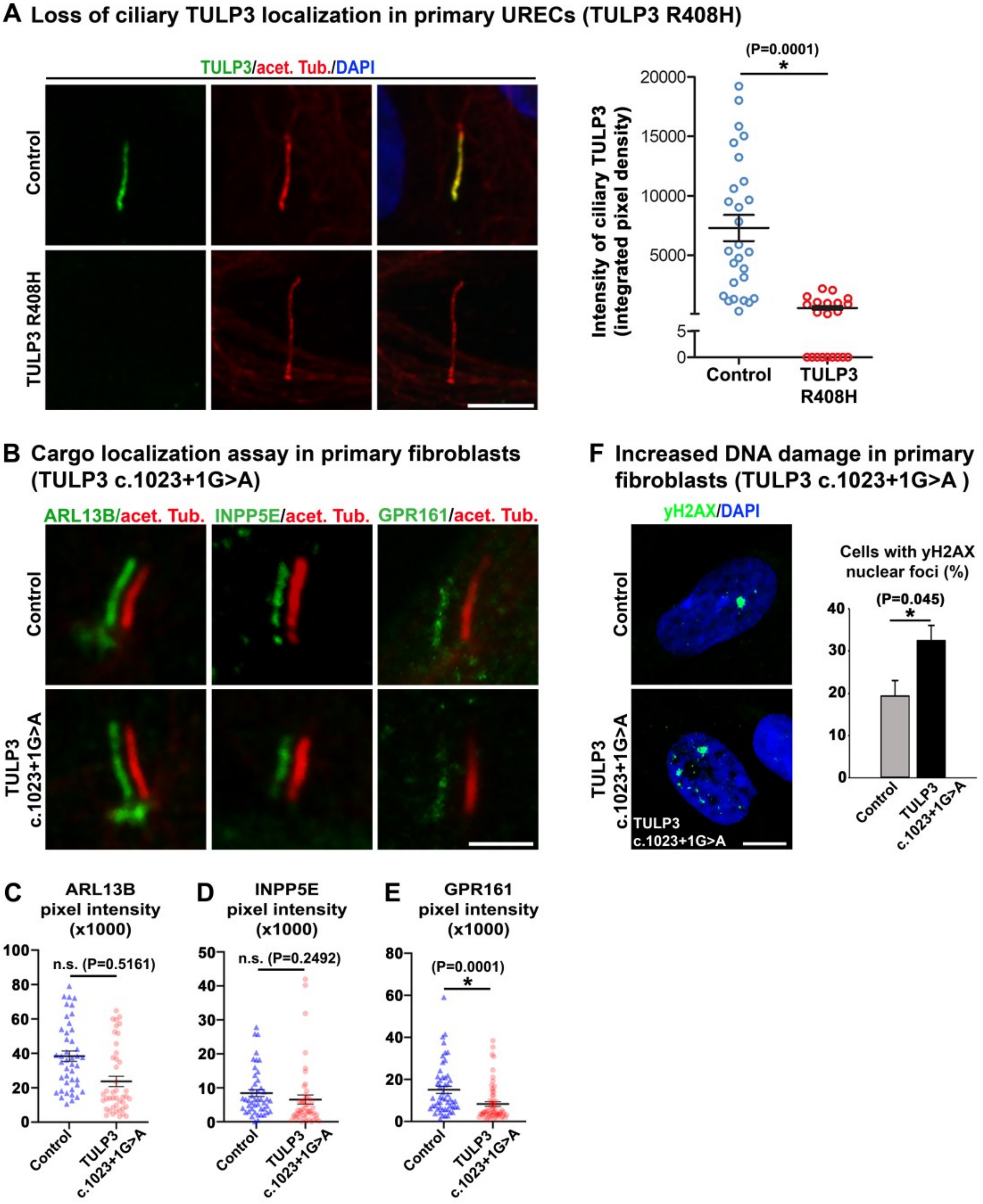
A Loss of ciliary TULP3 localization in primary URECs (TULP3 R408H)
TULP3/acet. Tub./DAPI
Control
TULP3 R408H
(P=0.0001)
*
Intensity of ciliary TULP3 (integrated pixel density)
20000
15000
10000
5000
5
0
Control
TULP3 R408H
B Cargo localization assay in primary fibroblasts (TULP3 c.1023+1G>A)
ARL13B/acet. Tub.
INPP5E/acet. Tub.
GPR161/acet. Tub.
Control
TULP3 c.1023+1G>A
F Increased DNA damage in primary fibroblasts (TULP3 c.1023+1G>A)
yH2AX/DAPI
Control
TULP3 c.1023+1G>A
TULP3 c.1023+1G>A
Cells with yH2AX nuclear foci (%)
(P=0.045)
*
40
30
20
10
0
Control
TULP3 c.1023+1G>A
C ARL13B pixel intensity (x1000)
100
80
60
40
20
0
n.s. (P=0.5161)
Control
TULP3 c.1023+1G>A
D INPP5E pixel intensity (x1000)
50
40
30
20
10
0
n.s. (P=0.2492)
Control
TULP3 c.1023+1G>A
E GPR161 pixel intensity (x1000)
80
60
40
20
0
(P=0.0001)
*
Control
TULP3 c.1023+1G>A

Figure S4. Ciliary trafficking in additional cells derived from affected individuals.

Panel A Left: Confocal microscope images showing ciliary localisation of TULP3 in control URECs. In contrast, ciliary expression of TULP3 is lost in URECs of affected individual Family 2 (II.1) (p.(Arg408His)). Right: Relative intensity of ciliary TULP3 signal in control URECs and URECs derived from the affected individual represented as integrated pixel intensity. **Panel B** shows localisation of TULP3 cargos in cilia from control fibroblasts and fibroblasts from Family 3 proband (II.2) (c.1023+1G>A). Color channels are offset to better visualize the signals. **Panel C-E** shows quantification of TULP3 cargo (ARL13B, INPP5E and GPR161) intensity in control fibroblasts and fibroblasts from the affected individual represented as pixel intenisities. **Panels F** shows increased γH2AX nuclear foci in fibroblasts from Family 3 proband (II.2) compared to age-matched controls. For panels A-F: * $P<0.05$ (two-tailed, unpaired student's t-test); error bars represent SEM; scale bars, 5 μm.

A TULP3 physical interaction network (TAP)

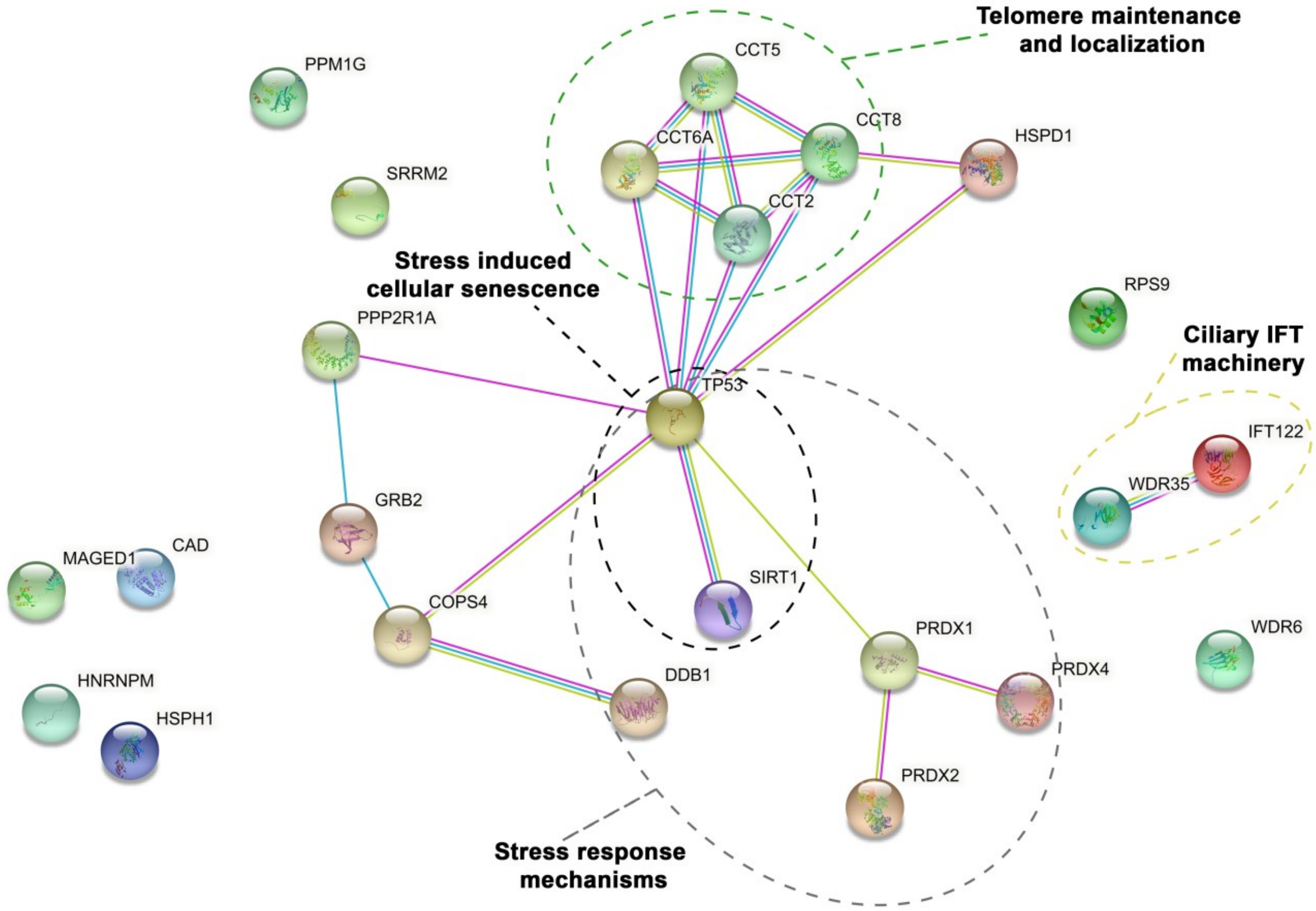

B Functional enrichment (GO: Biological processes)

#term ID	Term description	Observed gene count	Background gene count	Strength	False discovery rate
GO:0051097	negative regulation of helicase activity	2	5	2.51	0.0135
GO:1904851	positive regulation of establishment of protein localization to telomere	4	10	2.51	3.01E-05
GO:1904871	positive regulation of protein localization to Cajal body	4	11	2.47	3.01E-05
GO:1904874	positive regulation of telomerase RNA localization to Cajal body	4	15	2.34	3.01E-05
GO:0090400	stress-induced premature senescence	2	8	2.31	0.023
GO:0035721	intraciliary retrograde transport	2	10	2.21	0.0305
GO:0019430	removal of superoxide radicals	2	13	2.1	0.0385
GO:0045722	positive regulation of gluconeogenesis	2	14	2.07	0.0404
GO:0070914	UV-damage excision repair	2	14	2.07	0.0404
GO:0090343	positive regulation of cell aging	2	15	2.04	0.0424

Figure S5. TULP3 physical interaction network: DNA damage response and ciliary transport.

Panel A shows a STRING database interaction network generated with the TULP3 interaction partners identified through affinity purification mass spectroscopy using N-terminally FLAG-tagged TULP3 as bait in HEK293T cells. Physical interaction network visualised using the

STRING online tool with default settings (V11.5). Medium confidence (0.400). Dotted circles denote proteins with shared functions (summarized from functional enrichment in Panel B). **Panel B** shows functional enrichment analysis of the identified TULP3 interaction partners. The table shows GO term - biological processes that are overrepresented in the list of TULP3 interaction partners. Strength refers to background gene count/observed gene count. Shown are the top 10 hits ordered by strength with corrected P-value <0.05. False discovery rate (FDR) determined using Benjamini-Hochberg procedure.

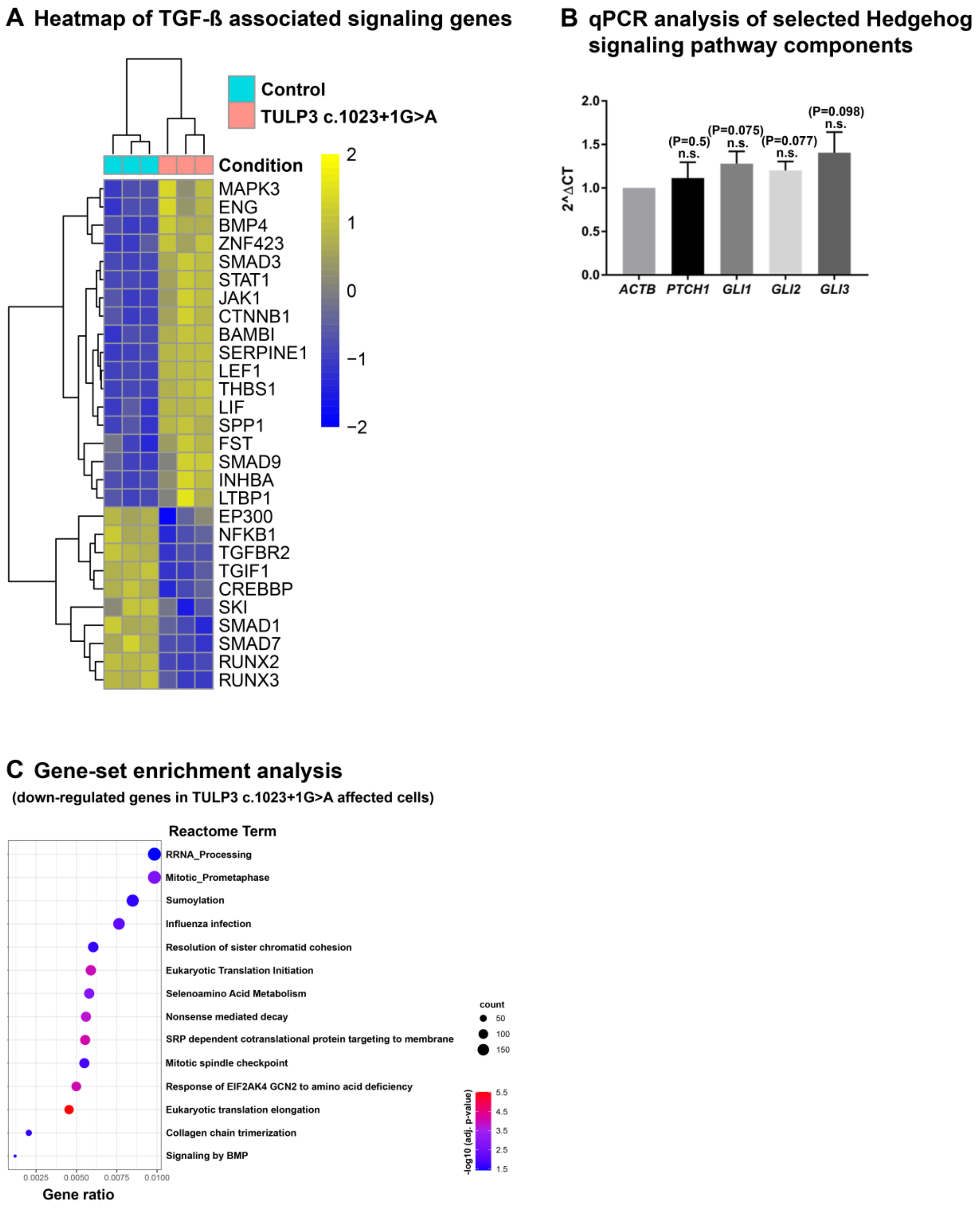

Figure S6. TGF-β pathway associated genes are significantly up-regulated in cells derived from *TULP3* proband.

Panel A shows a gene expression heatmap for differentially regulated genes from the GSEA term “TGF-β signaling pathway”. Dysregulated genes identified through RNA sequencing of fibroblast cells derived from Family 3 proband (II.2) are shown. Each column represents an individual sample from unaffected control or affected proband-derived cells. Yellow to blue

color-codes indicating up- or down-regulation are presented on the right; differentially expressed genes with P-values $P<0.05$ are shown. **Panel B** shows qPCR analysis of selected SHH pathway components in fibroblasts derived from Family 3 proband (II.2). *ACTB* used as housekeeping gene. n.s.= $P>0.05$; error bars represent SEM (two-tailed, unpaired student's t-test). **Panel C** shows reactome terms of significantly down-regulated genes in fibroblast cells derived from Family 3 proband (II.2). Differentially regulated genes were identified by the Gene set analysis method. Pathways were considered significant with adjusted p-values (Benjamini-Hochberg) $P<0.05$. Significantly down-regulated genes associated predominantly with cell cycle, ribosome and circadian rhythm related signaling processes.

Supplemental Methods

Massively parallel sequencing

We utilized a customized sequence capture library (e.g. by Twist Bioscience©) with curated target regions - currently comprising more than 600 genes described and associated with kidney disease or allied disorders - as well as corresponding flanking intronic sequences according to the manufacturer´s recommendations for probands of Families 1, 3, 4 and 6. The panel design is constantly updated by surveillance of current literature as well as enriched by targets in non-coding regions for described variants listed in well-accepted databases like HGMD or ClinVar. Moreover, the design is optimized in low-performance regions as well as in critical regions like in *PKD1* as described [1,2]. DNA samples were pooled and sequenced in a multiplexing procedure. DNAs were enriched using a sequence capture approach and sequenced using Illumina sequencing-by-synthesis technology with an average coverage of more than 300X for a targeted panel setup. Raw data were processed according to bioinformatics best practice procedures. Mapping and coverage statistics were generated from the mapping output files using standard bioinformatics tools (e.g. Picard). High and reproducible coverage achieved by our sequencing approach enabled copy number variation (CNV) analysis. Performance of the wet-lab and bioinformatic processes are validated and controlled according to national and international guidelines [3,4] reaching high sensitivity for single nucleotide variants (SNVs), indels and CNVs using well-established reference samples as well as a large cohort of positive controls, especially for CNVs. For interpretation of identified variants, we have developed own published bioinformatic algorithms using a stepwise filtering process conducted by an experienced team of scientists and supported by various bioinformatics decision tools. Sequence variants of interest were verified by Sanger sequencing if next generation sequencing (NGS) results failed internal validation guidelines. If other family members were available, segregation of sequence variants with the disease was further assessed.

Families 2 and 5 were identified by whole genome sequencing (WGS) performed by Genomics England (GE). The 100,000 Genomes Project provides a rich source of WGS data on individuals with rare disease phenotypes. As of September 2019, WGS was performed in 35,042 probands affected by rare diseases, including 3,934 probands with various renal and urinary tract disorders. WGS was performed by GE via the 100,000 Genomes Project using the Illumina TruSeq DNA PCR-Free sample preparation kit (Illumina, Inc.) and an Illumina HiSeq 2500 sequencer, generating a mean depth of 45x (range from 34x to 72x) and greater than 15x for at least 95% of the reference human genome. WGS reads were aligned to the Genome Reference Consortium human genome build 37 (GRCh37/hg19) using Isaac Genome Alignment Software (version 01.14; Illumina, Inc.). Sequence data was analysed using bcftools scripts designed to search vcf.gz files.

GeneMatcher was used to identify additional cases in Families 7 and 8. GeneMatcher is a database developed as part of the MatchMaker Exchange and has been shown to facilitate rare disease gene discovery [5]. Genomic analysis for Family 7 was performed at Johns Hopkins as part of the Baylor-Hopkins Center for Mendelian Genomics project. For these affected individuals, libraries from genomic DNA were constructed using the Agilent SureSelect HumanAllExonV5Clinical_S06588914 kit to capture the total ~52 Mb CCDS exonic regions as well as flanking intronic regions. Whole exome sequencing (WES) was performed using the Illumina HiSeq 2500 sequencer. WES reads were aligned to the 1000 Genomes phase 2 (GRCh37) human genome reference build with the Burrows-Wheeler Alignment tool version 0.7.8 [6] and Genome Analysis Toolkit (GATK) version v3.1-1-g07a4bf8 or v3.3-0-g37228af [7]. Variants were subsequently filtered using the Variant Quality Score Recalibration method [8,9]. Annotation of SNVs was performed using the MQRankSum, HaplotypeScore, QD, FS, MQ, ReadPosRankSum adaptive error model.

Imaging data and histology of liver, kidney and heart tissues of affected individuals

Tissue samples were taken for histopathological diagnosis and processed in the institutes of pathology according to the standard protocols for routine diagnostics.

Immunofluorescence analysis of cells derived from affected individuals

For cilia imaging, human urine-derived renal epithelial cells (URECs) were seeded on coverslips and grown to 90% confluence then starved for 48 h (in FBS-free media). Cells were fixed in ice-cold methanol for 10 min. After 30 min saturation with 5% BSA in PBS, cells were incubated for 1 h at room temperature (RT) with primary antibodies. Fibroblast cells were seeded on ibidi slides, grown to 90% confluence and starved for 72 h (in media with 0.1% FBS). These cells were fixed in 4% PFA for 5 min. After 1 h saturation with 5% BSA in PBS, fibroblasts were incubated over night at 4°C with primary antibodies. The following primary antibodies were used: mouse anti-acetylated α-Tubulin, rabbit anti-ARL13B, mouse anti-ARL13B, rabbit anti-INPP5E, rabbit anti-GPR161, rabbit anti-TULP3, rabbit anti-γH2AX (listed below) followed by washing steps in PBS. Cells were incubated at RT for 1 h with the following secondary antibodies: donkey anti-rabbit Alexa Fluor 488; donkey anti-mouse Alexa Fluor 594. Nuclei were counterstained with 4′,6-diamidino-2-phenylindole (DAPI) using NucBlueTM (Thermo Fischer Scientific, R37606). Images and z-stacks of URECs were captured using a Nikon (A1) confocal inverted microscope. Images and z-stacks of fibroblasts were captured using a Zeiss LSM 880 Observer confocal inverted microscope. Identical laser intensity as well as camera settings were used for control and cells derived from affected individuals (unless specified otherwise). Following capture, images were analysed using FIJI (ImageJ) software. Fluorescence intensity of individual cilia or nuclei were measured on a sum of slices projection of a z-stack. A region of interest (ROI) was constructed around the cilia (identified with ARL13B staining or acetylated α-Tubulin staining) or the nucleus (identified with DAPI) to measure the integrated density. To correct for local background intensity, the ROI was duplicated and dragged to a nearby region and background integrated density was measured and subtracted. To detect the fraction of GPR161-positive cilia, ROI around cilia were constructed using ARL13B staining. ROI were then transposed on the GPR161 z-stack channel that was visually screened for GPR161 signal in control cells and cells derived from affected individuals under identical laser intensity and camera settings.

Target protein	Use	Dilution	Company	Comment	Species
acetylated α-Tubulin	IF	1:2000, 1:10000	Sigma, T6793		Mouse
ARL13B	IF	1:300	Proteintech,17711-1-AP		Rabbit
ARL13B	IF	1:300	Proteintech, 66739-1-IG		Mouse
INPP5E	IF	1:100-1:200	Proteintech,17797-1-AP		Rabbit
TULP3	IF	1:300-1:500	Abcam, ab155317	detects aa129-403	Rabbit
GPR161	IF	1:100	obtained from S. Mukhopadhyay		Rabbit
γH2AX	IF	1:100	Cell Signaling, mAb, #9718		Rabbit
Alexa Fluor 488	IF	1:300	Invitrogen, A21206		Rabbit
Alexa Fluor 488	IF	1:1000	Invitrogen, A11034		Rabbit
Alexa Fluor 594	IF	1:300	Invitrogen, A21203		Mouse
CY-3	IF	1:1000	Jackson Laboratory, 715-165-150		Mouse

Reverse-transcription polymerase chain reaction (RT-PCR) analysis for zebrafish

Semi-quantitative RT-PCR was performed to determine expression of zebrafish *tulp3* during embryonic development and in adult organs. Total RNA from entire zebrafish embryos or adult zebrafish organs was extracted with the RNeasy Kit (Qiagen), followed by complementary DNA (cDNA) synthesis with the ProtoScript First Strand cDNA Synthesis Kit (Promega). Analysis of zebrafish *ef1α* was used as a loading control. The following primers were used for temporal and spatial PCR analysis: tulp3 (forward: 5'-AGAACCTCATCGAGCTGCAT-3'; reverse: 5'-ATGTGTGTGTGTCTCAGGGT-3'), ef1α (forward: 5'-ATCTACAAATGCGGTGGAAT-3'; reverse: 5'-ATACCAGCCTCAAACTCACC-3'). The following primers were used to determine *tulp3* expression in MZ*tulp3* and respective control: tulp3 (forward: 5'-TCTGCTGGAGCAGAAGCAG-3'; reverse: 5'-GTGGATTTAGTGCTGGATGCAGAC-3').

Generation of CRISPR/Cas9-induced *tulp3* mutant zebrafish

As described previously [9,10], double-stranded tulp3 gRNAs (*tulp3*_gRNA-f 5`-TAGGGGGTTCAGAAGCTCCTGATC-3`, tulp3_gRNA-r 5`-

AAACGATCAGGAGCTTCTGAACCC-3`) were cloned into BsmBI-linearized pT7-gRNA vector (Addgene). BamHI-linearized pT7-*tulp3*-gRNA was used to transcribe *tulp3-gRNA* mRNA using the MEGAshortscript T7 kit (Thermo Fisher Scientific). XbaI-linearized pT3TS-nCas9n vector (Addgene) was used to transcribe *Cas9* mRNA using the T3 mMessage mMachine™ kit (Thermo Fisher Scientific). 1 nL containing a mixture of 50 pg *tulp3-gRNA* mRNA and 300 pg *Cas9* mRNA was injected into *Tg(wt1b:GFP)* zebrafish embryos at the 1-cell stage. For validation of gRNA efficiency genomic DNA was amplified using the following primers: tulp3e5-f 5`-TTACTGCTGACTGGCTGCAT-3` and tulp3e5-r 5`-CTGCCAGACAGACCTGAGAA-3` followed by analysis of Sanger-sequenced products. Analysis of genomic DNA of adult zebrafish identified potential founders which were outcrossed several times to *Tg(wt1b:EGFP)* zebrafish.

Microtome sectioning and histological analysis of adult zebrafish tissue

Adult zebrafish were sacrificed by immersion in tricaine (MS-222) containing Danieau's, fixed in 4% PFA for seven days, changing the fixative solution at least once. Specimens were stored in 70% EtOH until use. Whole organs were removed by dissection under a dissection microscope. Prior to embedding, samples were dehydrated in graded EtOH baths (25%, 50%, 75%, 100%), followed by graded clearance in Histoclear (Thermo Fisher Scientific) (25%, 50%, 75%, 100%). Samples were then incubated at 60°C and gradually transferred from Histoclear into paraffin wax (25%, 50%, 75%, 100%). Samples were then embedded in paraffin blocks using HistoCore Arcadia embedding module (Leica) and sectioned using a Leica RM2255 rotary microtome at 3 or 5 µm thickness and mounted on frosted microscope slides. For tissue sections of zebrafish liver, kidney and heart, H&E and PAS stains were performed under standardized conditions at the Institute of Surgical Pathology, University Medical Center Freiburg.

Cystic kidney index

Adult zebrafish kidneys were isolated, sectioned and stained with H&E as above, in the coronal plane. Light-Field microscope images of the sections were then analysed to determine

total area of kidney tissue and total luminal area. Cystic index score was obtained by calculating the amount of luminal area as a percentage of the total area of the kidney. Area measurements were performed blinded using ImageJ.

Mass spectroscopy

SILAC labelled human embryonic kidney 293T (HEK293T) cells were transiently transfected with pcDNA6-FLAG-TULP3 or pcDNA6-FLAG-GFP and lysed in IP buffer supplemented with PMSF and Na_3VO_4 (Sigma). Cell lysates were incubated with anti-FLAG M2 agarose beads for 2 h at 4°C. Beads were washed in IP buffer and incubated with Laemmli buffer for 5 min at 95°C. Samples were then treated with iodoacetamide (IAA) and excess IAA was quenched by addition of DTT. Mass spectroscopy was performed and analysed at the core facility proteomics at the Center of Biological Systems Analysis in Freiburg. Identified proteins were subjected to the STRING (https://string-db.org/ V11.5) protein association network database and a physical interaction network was generated using default settings [11].

Co-immunoprecipitation and western blotting

Calcium phosphate transfection was used to transiently express 2.5 μg of pcDNA6-FLAG-TULP3 and 2.5 μg of pcDNA6-SIRT1-V5 plasmid DNA in HEK293T cells. Cells were washed with ice-cold PBS and lysed in lysis buffer (1% Triton X-100; 20 mM Tris, pH 7.5; 50 mM NaF; 15 mM $Na_4P_2O_7$; 0.1 mM EDTA; supplemented with Na_3VO_4 and protease inhibitor mix (Roche)). Lysates were incubated with anti-FLAG M2 agarose beads or anti-V5 agarose beads for 2 h at 4°C. Western blots of lysates and immunoprecipitations were blocked in 5% BSA, incubated with anti-FLAG (Sigma) and anti-V5 (Sigma) and respective HRP conjugated antibodies. For protein detection the WesternBright ECL HRP substrate (Advansta) was used and immunoblot signals were quantified using Gel-Pro Analyzer 6.0, INTAS.

RNA sequencing analysis

RNA was isolated from affected proband-derived (Family 3 (II.2)) and control fibroblasts following 72 h incubation in DMEM (0.1% FBS) with three biological repeats for each condition.

Generation of the template library using the Illumina TruSeq stranded protocol, sequencing reactions on a HiSeq system (Illumina), initial bioinformatics processing and quality control were carried out by FASTERIS SA NGS services, Switzerland. Relative expression of genes was determined by comparing fragments per kilobase of transcript per million mapped reads (FPKM) between affected proband-derived (Family 3 (II.2)) and control samples. The raw RNA sequencing files were pre-processed with trimmomatic to ensure sufficient read quality by removing adapters and reads in low-quality segment regions with a base quality below 20. Subsequently, the reads were 2-pass aligned using the STAR aligner [14] and the GRCh37 reference genome from Ensembl. Alignment was followed by normalization and differential expression analysis with the R/Bioconductor [12,13] package DESeq2 [15]. Genes were considered significant with an adjusted P-value<0.05 (FDR corrected, according to Benjamini-Hochberg).

Gene set enrichment analysis

Enrichment analysis of signaling pathways was performed as implemented in the signaling pathways from Consensus Path DB [16]. Pathways were considered significant with an adjusted P-value<0.05 (Benjamini-Hochberg).

Quantitative PCR analysis of fibroblast cells and zebrafish embryos

For analysis of HH signaling in fibroblasts of affected individual II.2 of Family 3, total RNA from age and sex-matched healthy control and affected fibroblast cells was isolated with the RNeasy Kit (Qiagen), followed by cDNA synthesis with the Superscript II First strand cDNA Synthesis Kit (Thermo Scientific). The following primers were used for quantitative PCR (qPCR) analysis: ACTB (forward: 5'-CCAACCGCGAGAAGATGA-3'; reverse: 5'-CCAGAGGCGTACAGGGATAG-3'), GLI1 (forward: 5'-GGGATGATCCCACATCCTCAGTC -3'; reverse: 5'-CTGGAGCAGCCCCCCCAG-3'), GLI2 (forward: 5'-TGGCCGCCTCAGATGACAGATGTTG-3'; reverse: 5'-CGTTAGCGGAATGTCAGCCGTGAAG-3'), GLI3 (forward: 5'-GGCCATCCACATGGAATATC-3'; reverse: 5'-TGAAGAGCTACGGGAAT-3'), PTCH1

(forward: 5'-TGGAAGAAAACAAACAGCTTCC-3'; reverse: 5'-TCCCAGTCACTGTCAAATGC-3'). For analysis of *tulp3* expression in zebrafish, total RNA was isolated from 30 MZ*tulp3* mutant zebrafish embryos or respective control embryos (*tulp3* +/+) at 1 day post fertilization (dpf) using the RNeasy Kit, followed by cDNA synthesis with the Reliance Select cDNA Synthesis Kit (Biorad). The following primers were used for qPCR analysis: tulp3 (forward: 5'-AGAACCTCATCGAGCTGCAT-3'; reverse: 5'-AGAGTGAAGATGTCCTCCGC-3'), ef1α (forward: 5'-TGCCAACTTCAACGCTCAGGT-3'; reverse: 5'-TCAGCAAACTTGCAGGCGATG-3'). The DyNAmo ColorFlash SYBR Green Kit (Thermo Fisher Scientific) was used, and qPCR was performed on a Light Cycler 480 (Roche) at the Lighthouse Core Facility at the University Clinics in Freiburg.

Acknowledgements

We thank the families for participating in this study. We are grateful to Prof. Djengel and Veronica Dumit for discussion and performance of the mass spectroscopy analysis at the Proteomics Core Facility in Freiburg. We thank Alena Sammarco at the Institute of Pathology, University Clinics Freiburg, for substantial help with histological sections and stainings of adult zebrafish tissues. We thank Dr. Elisa Molinari for critical help with patient-derived urinary cell studies. We are grateful to Saikat Mukhopadhyay (UT Southwestern Medical Center) for anti-GPR161 antibodies.

Genomics England Research Consortium (27th May 2021)

John C. Ambrose[1]; Prabhu Arumugam[1]; Roel Bevers[1]; Marta Bleda[1] ; Freya Boardman-Pretty[1,2]; Christopher R. Boustred[1]; Helen Brittain[1]; Mark J. Caulfield[1,2]; Georgia C. Chan[1]; Greg Elgar[1,2]; Tom Fowler[1]; Adam Giess[1]; Angela Hamblin[1]; Shirley Henderson[1,2]; Tim J. P. Hubbard[1]; Rob Jackson[1]; Louise J. Jones[1,2]; Dalia Kasperaviciute[1,2]; Melis Kayikci[1]; Athanasios Kousathanas[1]; Lea Lahnstein[1]; Sarah E. A. Leigh[1]; Ivonne U. S. Leong[1]; Javier F. Lopez[1]; Fiona Maleady-Crowe[1]; Meriel McEntagart[1]; Federico Minneci[1]; Loukas Moutsianas[1,2]; Michael Mueller[1,2]; Nirupa Murugaesu[1]; Anna C. Need[1,2]; Peter O'Donovan[1]; Chris A. Odhams[1]; Christine Patch[1,2]; Mariana Buongermino Pereira[1]; Daniel Perez-Gil[1]; John Pullinger[1]; Tahrima Rahim[1]; Augusto Rendon[1]; Tim Rogers[1]; Kevin Savage[1]; Kushmita Sawant[1]; Richard H. Scott[1]; Afshan Siddiq[1]; Alexander Sieghart[1]; Samuel C. Smith[1]; Alona Sosinsky[1,2]; Alexander Stuckey[1]; Mélanie Tanguy[1]; Ana Lisa Taylor Tavares[1]; Ellen R. A. Thomas[1,2]; Simon R. Thompson[1]; Arianna Tucci[1,2]; Matthew J. Welland1; Eleanor Williams[1]; Katarzyna Witkowska[1,2]; Suzanne M. Wood[1,2].

[1] Genomics England, London, UK.

[2] William Harvey Research Institute, Queen Mary University of London, London, EC1M 6BQ, UK.

Supplementary Web Resources

ConsensusPathDB interaction database: http://consensuspathdb.org/

Clustal omega: https://www.ebi.ac.uk/Tools/msa/clustalo/

STRING: https://string-db.org/

Supplementary References

1. Lu, H., Galeano, M.C.R., Ott, E., Kaeslin, G., Kausalya, P.J., Kramer, C., Ortiz-Brüchle, N., Hilger, N., Metzis, V., Hiersche, M., et al. (2017). Mutations in DZIP1L, which encodes a ciliary-transition-zone protein, cause autosomal recessive polycystic kidney disease. Nat. Genet. *49*, 1025–1034.

2. Schrezenmeier, E., Kremerskothen, E., Halleck, F., Staeck, O., Liefeldt, L., Choi, M., Schüler, M., Weber, U., Bachmann, N., Grohmann, M., et al. (2021). The underestimated burden of monogenic kidney disease in adults waitlisted for kidney transplantation. Genet. Med. Off. J. Am. Coll. Med. Genet. *23*, 1219–1224.

3. Rehm, H.L., Bale, S.J., Bayrak-Toydemir, P., Berg, J.S., Brown, K.K., Deignan, J.L., Friez, M.J., Funke, B.H., Hegde, M.R., Lyon, E., et al. (2013). ACMG clinical laboratory standards for next-generation sequencing. Genet. Med. *15*, 733–747.

4. Matthijs, G., Souche, E., Alders, M., Corveleyn, A., Eck, S., Feenstra, I., Race, V., Sistermans, E., Sturm, M., Weiss, M., et al. (2016). Guidelines for diagnostic next-generation sequencing. Eur. J. Hum. Genet. EJHG *24*, 2–5.

5. Sobreira, N., Schiettecatte, F., Valle, D., and Hamosh, A. (2015). GeneMatcher: a matching tool for connecting investigators with an interest in the same gene. Hum. Mutat. *36*, 928–930.

6. Li, H. (2013). Aligning sequence reads, clone sequences and assembly contigs with BWA-MEM. ArXiv Genomics.

7. McKenna, A., Hanna, M., Banks, E., Sivachenko, A., Cibulskis, K., Kernytsky, A., Garimella, K., Altshuler, D., Gabriel, S., Daly, M., et al. (2010). The Genome Analysis Toolkit: a MapReduce framework for analyzing next-generation DNA sequencing data. Genome Res. *20*, 1297–1303.

8. DePristo, M.A., Banks, E., Poplin, R., Garimella, K.V., Maguire, J.R., Hartl, C., Philippakis, A.A., del Angel, G., Rivas, M.A., Hanna, M., et al. (2011). A framework for variation discovery and genotyping using next-generation DNA sequencing data. Nat. Genet. *43*, 491–498.

9. Van der Auwera, G.A., Carneiro, M.O., Hartl, C., Poplin, R., Del Angel, G., Levy-Moonshine, A., Jordan, T., Shakir, K., Roazen, D., Thibault, J., et al. (2013). From FastQ data to high confidence variant calls: the Genome Analysis Toolkit best practices pipeline. Curr. Protoc. Bioinforma. *43*, 11.10.1-11.10.33.

10. Epting, D., Senaratne, L.D.S., Ott, E., Holmgren, A., Sumathipala, D., Larsen, S.M., Wallmeier, J., Bracht, D., Frikstad, K.-A.M., Crowley, S., et al. (2020). Loss of CBY1 results in a ciliopathy characterized by features of Joubert syndrome. Hum. Mutat. *41*, 2179–2194.

11. Szklarczyk, D., Gable, A.L., Nastou, K.C., Lyon, D., Kirsch, R., Pyysalo, S., Doncheva, N.T., Legeay, M., Fang, T., Bork, P., et al. (2021). The STRING database in 2021: customizable protein–protein networks, and functional characterization of user-uploaded gene/measurement sets. Nucleic Acids Res. *49*, D605–D612.

12. R Development Core Team (2008). R: A language and environment for statistical computing (Vienna, Austria: R Foundation for Statistical Computing).

13. Gentleman, R.C., Carey, V.J., Bates, D.M., Bolstad, B., Dettling, M., Dudoit, S., Ellis, B., Gautier, L., Ge, Y., Gentry, J., et al. (2004). Bioconductor: open software development for computational biology and bioinformatics. Genome Biol. *5*, R80.

14. Dobin, A., Davis, C.A., Schlesinger, F., Drenkow, J., Zaleski, C., Jha, S., Batut, P., Chaisson, M., and Gingeras, T.R. (2013). STAR: ultrafast universal RNA-seq aligner. Bioinforma. Oxf. Engl. *29*, 15–21.

15. Love, M., Anders, S., and Huber, W. (2014). Differential analysis of count data - the DESeq2 package. p.

16. Kamburov, A., Stelzl, U., Lehrach, H., and Herwig, R. (2013). The ConsensusPathDB interaction database: 2013 update. Nucleic Acids Res. *41*, D793–D800.
