# Supplementary material for: Progressive liver, kidney, and heart degeneration in children and adults affected by TULP3 mutations
Source: Am J Hum Genet. 2022 Apr 8;109(5):928–43. doi: 10.1016/j.ajhg.2022.03.015 (PMC9118107; doi:10.1016/j.ajhg.2022.03.015)
Supplement: Document S2. Article plus supplemental information [file mmc4.pdf]

Progressive liver, kidney, and heart degeneration in children and adults affected by *TULP3* mutations

Graphical abstract

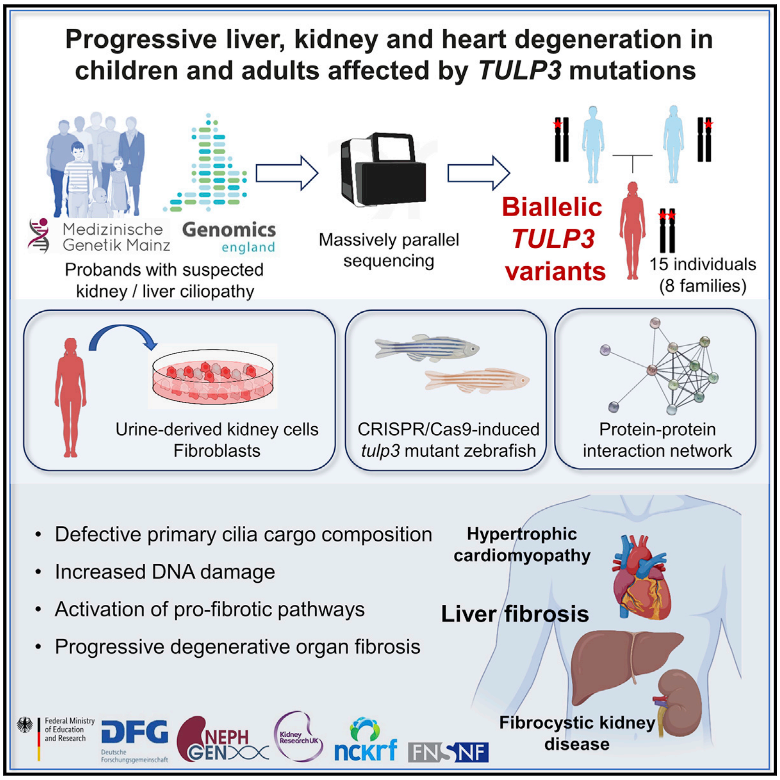

Authors

John Devane, Elisabeth Ott, Eric G. Olinger, ..., Bernhard Schlevogt, John A. Sayer, Carsten Bergmann

Correspondence

john.sayer@newcastle.ac.uk (J.A.S.),
carsten.bergmann@medgen-mainz.de (C.B.)

Devane et al., 2022, The American Journal of Human Genetics *109*, 928–943
May 5, 2022 © 2022 The Author(s).
https://doi.org/10.1016/j.ajhg.2022.03.015

ARTICLE

Progressive liver, kidney, and heart degeneration in children and adults affected by *TULP3* mutations

John Devane,[1,25] Elisabeth Ott,[1,25] Eric G. Olinger,[2,25] Daniel Epting,[1,25] Eva Decker,[3] Anja Friedrich,[3] Nadine Bachmann,[3] Gina Renschler,[3] Tobias Eisenberger,[3] Andrea Briem-Richter,[4] Enke Freya Grabhorn,[4] Laura Powell,[2] Ian J. Wilson,[5] Sarah J. Rice,[5] Colin G. Miles,[2] Katrina Wood,[6] Genomics England Research Consortium, Palak Trivedi,[7,8,9,10] Gideon Hirschfield,[11] Andrea Pietrobattista,[12] Elizabeth Wohler,[14] Anya Mezina,[15] Nara Sobreira,[14] Emanuele Agolini,[13] Giuseppe Maggiore,[12] Mareike Dahmer-Heath,[16] Ali Yilmaz,[17] Melanie Boerries,[18,19] Patrick Metzger,[18] Christoph Schell,[20] Inga Grünewald,[21] Martin Konrad,[16] Jens König,[16] Bernhard Schlevogt,[22] John A. Sayer,[2,23,24,*] and Carsten Bergmann[1,3,*]

Summary

Organ fibrosis is a shared endpoint of many diseases, yet underlying mechanisms are not well understood. Several pathways governed by the primary cilium, a sensory antenna present on most vertebrate cells, have been linked with fibrosis. Ciliopathies usually start early in life and represent a considerable disease burden. We performed massively parallel sequencing by using cohorts of genetically unsolved individuals with unexplained liver and kidney failure and correlated this with clinical, imaging, and histopathological analyses. Mechanistic studies were conducted with a vertebrate model and primary cells. We detected bi-allelic deleterious variants in *TULP3*, encoding a critical adaptor protein for ciliary trafficking, in a total of 15 mostly adult individuals, originating from eight unrelated families, with progressive degenerative liver fibrosis, fibrocystic kidney disease, and hypertrophic cardiomyopathy with atypical fibrotic patterns on histopathology. We recapitulated the human phenotype in adult zebrafish and confirmed disruption of critical ciliary cargo composition in several primary cell lines derived from affected individuals. Further, we show interaction between TULP3 and the nuclear deacetylase SIRT1, with roles in DNA damage repair and fibrosis, and report increased DNA damage *ex vivo*. Transcriptomic studies demonstrated upregulation of profibrotic pathways with gene clusters for hypertrophic cardiomyopathy and WNT and TGF-β signaling. These findings identify variants in *TULP3* as a monogenic cause for progressive degenerative disease of major organs in which affected individuals benefit from early detection and improved clinical management. Elucidation of mechanisms crucial for DNA damage repair and tissue maintenance will guide novel therapeutic avenues for this and similar genetic and non-genomic diseases.

Introduction

Fibrosis is the result of maladaptive processes leading to an excessive accumulation and deposition of extracellular matrix (ECM) and connective tissue and often culminates in large scale disruption of tissue architecture. Chronic fibrosis of organs can lead to progressive decline in function as ECM slowly replaces parenchymal tissue and may result in organ failure over many years.[1,2]

The mechanism of fibrosis is closely linked to normal wound healing (reviewed in Rockey et al.[3]) and usually involves both intrinsic susceptibility and predisposing factors such as exposure to genotoxins or aging.[4] Fibrosis is estimated to be a contributing factor in 45% of deaths in the United States.[5] Monogenic diseases linked to fibrosis offer a unique opportunity to untangle intrinsic pathways from external factors and prioritize potential therapeutic targets.[4] High-throughput technologies have allowed the

[1]Department of Medicine IV, Faculty of Medicine, Medical Center-University of Freiburg, 79106 Freiburg, Germany; [2]Translational and Clinical Research Institute, Faculty of Medical Sciences, Newcastle University, Newcastle upon Tyne NE1 3BZ, UK; [3]Medizinische Genetik Mainz, Limbach Genetics, 55128 Mainz, Germany; [4]University Medical Center Hamburg-Eppendorf, Department of Pediatrics, 20251 Hamburg, Germany; [5]Biosciences Institute, Faculty of Medical Sciences, Newcastle University, Newcastle upon Tyne NE1 3BZ, UK; [6]Histopathology Department, The Newcastle upon Tyne Hospitals NHS Foundation Trust, Newcastle upon Tyne NE1 4LP, UK; [7]NIHR Birmingham BRC, Centre for Liver and Gastrointestinal Research, University of Birmingham, Birmingham B15 2TT, UK; [8]Liver Unit, University Hospitals Birmingham, Birmingham B15 2GW, UK; [9]Institute of Immunology and Immunotherapy, University of Birmingham, Birmingham B15 2TT, UK; [10]Institute of Applied Health Research, University of Birmingham, Birmingham B15 2TT, UK; [11]Toronto Centre for Liver Disease, University Health Network, Toronto, ON M6H 3M1, Canada; [12]Hepatogastroenterology and Liver Transplant Unit and Medical Genetics Laboratory, IRCCS Bambino Gesù Children's Hospital, 00165 Rome, Italy; [13]Translational Cytogenomics Research Unit, Bambino Gesù Children's Hospital, IRCCS, 00146 Rome, Italy; [14]McKusick-Nathans Department of Genetic Medicine, Johns Hopkins University School of Medicine, Baltimore, MD 21205, USA; [15]Department of Medicine, University of Pennsylvania Perelman School of Medicine, Philadelphia, PA 19104, USA; [16]Department of General Pediatrics, University Hospital Münster, 48149 Münster, Germany; [17]Department of Cardiology I, University Hospital Münster, 48149 Münster, Germany; [18]Institute of Medical Bioinformatics and Systems Medicine Medical Center – University of Freiburg, Medical Faculty, University of Freiburg, 79110 Freiburg, Germany; [19]The German Cancer Consortium, Partner Site Freiburg and Cancer Research Center, 69120 Heidelberg, Germany; [20]Institute for Pathology, Medical Center – University of Freiburg, Medical Faculty, University of Freiburg, 79002 Freiburg, Germany; [21]Institute for Pathology, University Hospital Münster, 48149 Münster, Germany; [22]Department of Internal Medicine B, Gastroenterology, University Hospital Münster, 48149 Münster, Germany; [23]Renal Services, The Newcastle upon Tyne Hospitals NHS Foundation Trust, Newcastle upon Tyne NE7 7DN, UK; [24]Newcastle Biomedical Research Centre, NIHR, Newcastle upon Tyne NE4 5PL, UK

[25]These authors contributed equally

*Correspondence: john.sayer@newcastle.ac.uk (J.A.S.), carsten.bergmann@medgen-mainz.de (C.B.)

https://doi.org/10.1016/j.ajhg.2022.03.015.

© 2022 The Author(s). This is an open access article under the CC BY license (http://creativecommons.org/licenses/by/4.0/).

detection of pathogenic mechanisms associated with progressive organ fibrosis, including DNA damage,[6] storage disorders,[7] and defective protein synthesis.[8] However, there is still comparatively little known about distinct underlying genetic mechanisms.

Dysfunction of a cell signaling organelle known as the primary cilium is at the origin of a group of human diseases referred to as ciliopathies that are also characterized by multisystem organ fibrosis via alterations in different molecular pathways.[9] Interestingly, several ciliary disease proteins colocalize to sites of DNA damage, linking a subset of ciliopathies with aberrant DNA damage response.[10] In addition to a wide spectrum of syndromic manifestations, ciliopathies can present with fibrocystic kidney diseases and periportal liver fibrosis due to ductal plate malformation.[11] Typically, affected individuals with recessive ciliopathies present with major organ disease early in life. In autosomal recessive polycystic kidney disease (ARPKD [MIM: 263200]) for instance, one quarter of affected individuals need renal replacement therapy by the age of 15 years and more than half show signs of portal hypertension by then.[12] Similarly, end-stage kidney disease due to nephronophthisis usually develops before adulthood.[9] These early presentations, often with considerable disease burden already manifest prenatally during embryonic development, limit our ability to investigate the initial triggers of organ fibrosis, to study the natural course of disease, and to provide therapeutic windows for potential interventional studies.

TULP3 (MIM: 604730) encodes a 442-amino acid protein (Tubby-like protein 3), containing an N-terminal intraflagellar transport A (IFT-A)-binding domain and a C-terminal tubby domain with ubiquitous expression.[13] Acting as an adaptor protein for the ciliary IFT-A machinery, cellular and mouse studies have established a critical role for TULP3 in ciliary trafficking of integral membrane proteins.[14–16] In addition, nuclear roles for TULP3 have been suggested.[17]

Here, we detected 15 individuals from eight unrelated families with bi-allelic variants in *TULP3*. Postnatal disease onset is variable, ranging from childhood to adulthood. The affected individuals we report here are mostly adults, in the 3rd through 7th decades of life, and present with progressive degenerative liver fibrosis with variable fibrocystic kidney disease and hypertrophic cardiomyopathy. Using an adult zebrafish model and cells derived from affected individuals, we propose a model of multisystem fibrosis originating from disrupted ciliary composition and DNA damage.

Material and methods

Full details of all methods can be found in the supplemental information.

Ethics statement

Human blood samples for DNA extraction were obtained with written informed consent. This study was approved by the Northeast - Newcastle & North Tyneside 1 Research Ethics Committee (18/NE/350), and the Genomics England 100,000 Genomes Project was approved by the Health Research Authority Research Ethics Committee East of England – Cambridge South (REC Ref 14/EE/1112). For the affected individuals recruited through the Johns Hopkins Baylor-Hopkins Center for Mendelian Genomics project (family 7), this study was approved by the Johns Hopkins and Baylor College of Medicine institutional review boards. All experiments involving zebrafish were approved by the ethical committee (Regierungspräsidium Freiburg, Baden-Württemberg, Germany).

Study design

All affected individuals and family members involved in this study gave their written informed consent for genetic testing. Full details of study cohorts are provided in the supplemental information. All other human blood samples, human urine-derived renal epithelial cells (URECs), and fibroblast cells were obtained with written informed consent.

Massively parallel sequencing

In this study, we utilized different approaches based on next-generation sequencing (NGS) technologies and comprehensive bioinformatic analyses described in detail elsewhere.[18,19]

Isolation of cells derived from affected individuals

URECs were isolated from urine collected from affected individual II.1 from family 2 and healthy age- and sex-matched controls. Primary fibroblasts were isolated from skin biopsies of affected individual II.2 from family 3 and affected individual II.4 from family 6 and age- and sex-matched controls. Primary cells were maintained as previously described.[19–21]

Zebrafish maintenance and strains

AB/TL wild-type and *Tg(wt1b:EGFP)*[22] zebrafish (*Danio rerio*) strains were raised under standard conditions at 28°C and staged as previously described.[23]

Generation of CRISPR-Cas9-induced *tulp3* mutant zebrafish

We used the CHOPCHOP online tool to design efficient guide RNAs (gRNAs) targeting genomic *tulp3* in zebrafish. We injected an exon 5 targeting *tulp3*-gRNA mRNA and *cas9* mRNA to generate a *tulp3* mutant allele. Sanger sequencing confirmed a 5 bp deletion in exon 5 of *tulp3* leading to a frameshift and subsequently to a premature stop codon. Homozygous *tulp3* mutant zebrafish (*tulp3 m/m*) were generated by crossing heterozygous *tulp3* (*tulp3 m/+*) zebrafish. Wild-type zebrafish (*tulp3 +/+*) from this cross were raised as clutchmate controls. Maternal-zygotic *tulp3* mutants (MZ*tulp3*) were obtained from incrosses of homozygous zebrafish.

Immunofluorescence

For cilia imaging, we seeded URECs and fibroblasts derived from affected individuals on coverslips, grew them to 90% confluence, and serum starved them to induce ciliation. Cells were then fixed and incubated in target primary and secondary antibodies solutions.

Mass spectroscopy

To determine potential interaction partners of TULP3, we used tandem affinity purification combined with mass spectrometric

protein identification. Interaction networks were generated by analysis of a list of identified interaction partners with the STRING Protein-Protein Interaction Networks Functional Enrichment Analysis online tool.[24]

Co-immunoprecipitation of TULP3 and SIRT1

We co-transfected human TULP3 and SIRT1 in FLAG- and V5-tagged pcDNA6 vectors, respectively, into HEK293T cells by using calcium phosphate transfection. We then purified cell lysates by using agarose beads conjugated to FLAG- or V5-tag and performed immunoblot analyses by using antibodies against the reciprocal tag.

DNA damage response assay

We assessed DNA damage response (DDR) in affected individual's URECs (family 2 [II.1]) and fibroblasts (family 3 [II.2]) by using immunofluorescence imaging with the DDR marker γH2AX (Cell Signaling and Abcam). Nuclear staining intensity and punctae positive for γH2AX were then quantified and compared in age-, sex-, and passage-matched-control- and affected-individual-derived cells.

RNA sequencing analysis

RNA sequencing was performed by FASTERIS SA, NGS services. With the QIAGEN RNeasy extraction kit, total RNA was isolated from affected individual (family 3 [II.2]) and age- and sex-matched control fibroblasts following 72 h incubation in DMEM (0.1% FBS) with three biological repeats for each condition.

Statistical analysis

All data represent results from one of at least three independent experiments, which showed consistent results. Data were analyzed by Student's t test (two-sided, unpaired) and error bars represent the standard error of the mean (SEM) unless otherwise stated.

Results

Identification of bi-allelic *TULP3* variants in individuals with progressive liver, kidney, and heart disease

We used whole-exome sequencing (WES) or targeted exome sequencing (TES) including >600 genes with a known or hypothesized association to cystic kidney disease and other ciliopathies and kidney disorders (see supplemental methods)[19] to detect pathogenic variants in a cohort of individuals affected with fibrocystic liver/kidney disease or other ciliopathy-associated diseases. We first investigated a German family from our fibrocystic liver/kidney disease cohort with two affected brothers aged 65 and 68 years, both presenting with slowly progressing liver disease, initiating at around 20 years of age with elevated liver enzymes together with clinical features of portal hypertension, fibrocystic kidney disease leading to end-stage kidney disease (at 55 and 51 years, respectively), and hypertrophic non-obstructive cardiomyopathy (Figures 1A and 1B, Figures 2A and 2B, and Table 1). Using TES including copy number variant (CNV) analysis, we identified bi-allelic genetic variants in *TULP3* (GenBank: NM_003324.5) in both individuals (family 1: c.(41+1_42−1)_(696+1_697−1)del/c.612T>G [p.Cys204Trp]) (Figures S1 and S2). No further variants of relevance were detected. Notably, *Tulp3* knockout (KO) in mice leads to embryonic lethality and typical signs of aberrant ciliary signaling.[13,25,26] Interestingly, increased circulating bilirubin levels have been reported in *Tulp3* heterozygous null mice[27] and recent *Tulp3* hypomorphic mutant or nephron-specific KO mice displayed cystic kidney disease.[14,16] To find additional cases, we then (1) applied WES or TES in 5,124 genetically unsolved individuals from the same cohort of individuals with fibrocystic liver/kidney disease or other ciliopathy associated diseases,[19] (2) screened whole-genomic sequencing (WGS) data from the Genomics England 100,000 Genomes Project[28] (~35,000 probands with rare diseases, including ~1,500 probands with cystic kidney disease or unexplained kidney failure, ~800 with hypertrophic cardiomyopathy, and ~250 probands recruited under ductal plate malformation and/or cirrhosis), and (3) utilized GeneMatcher.[29] Altogether, we identified thirteen additional affected individuals from seven unrelated families (three from the fibrocystic liver/kidney disease cohort, two from Genomics England, and two through GeneMatcher) carrying bi-allelic predicted deleterious variants in *TULP3* that segregated with the disease phenotype and without detecting other variants of interest (family 2, c.1223G>A [p.Arg408His]; family 3, c.1023+1G>A; family 4, c.544delC [p.Leu182TrpfsTer4]; family 5, c.492+1G>A; family 6, c.1023+1G>A/c.70C>T [p.Arg24Ter]; family 7, c.925−1G>A; family 8, c.544delC [p.Leu182TrpfsTer4]) (Figure 1A, Figures S1 and S2, Table 1, and Table S1). Overall, we identified eight different genetic variants, among them six predicted high impact (multiexon deletion, nonsense, frameshifting, and canonical splice-affecting) and two missense (Table S1). The missense variants affect highly conserved residues within the functionally important tubby domain of TULP3 (Figures S1 and S3). Two splicing variants (c.925−1G>A and c.1023+1G>A) are predicted to lead to in-frame exon 9 skipping (99 bp) and removal of the 8th beta sheet of TULP3; this prediction was verified for family 3 (c.1023+1G>A) (Figures S3E–S3H).

Clinical and histopathological features of affected individuals with *TULP3* variants

In all affected individuals, the disease initially manifested with complications of liver disease (Figure 1B). Abnormal liver enzyme tests were the earliest sign of disease; in particular, biochemical markers of cholestasis were increased (Table 1). Younger affected individuals presented with cholestatic jaundice or abdominal pain, and one affected individual presented with gastro-intestinal bleeding secondary to portal hypertension. Liver disease progressed during childhood, and the earliest instances of liver transplantation were in the 3rd decade (families 6 and 7). The affected individuals in family 1 are the oldest surviving individuals without liver transplantation (65 and 68 years); one of the brothers is currently on the liver transplantation waiting list (Table 1). Computed

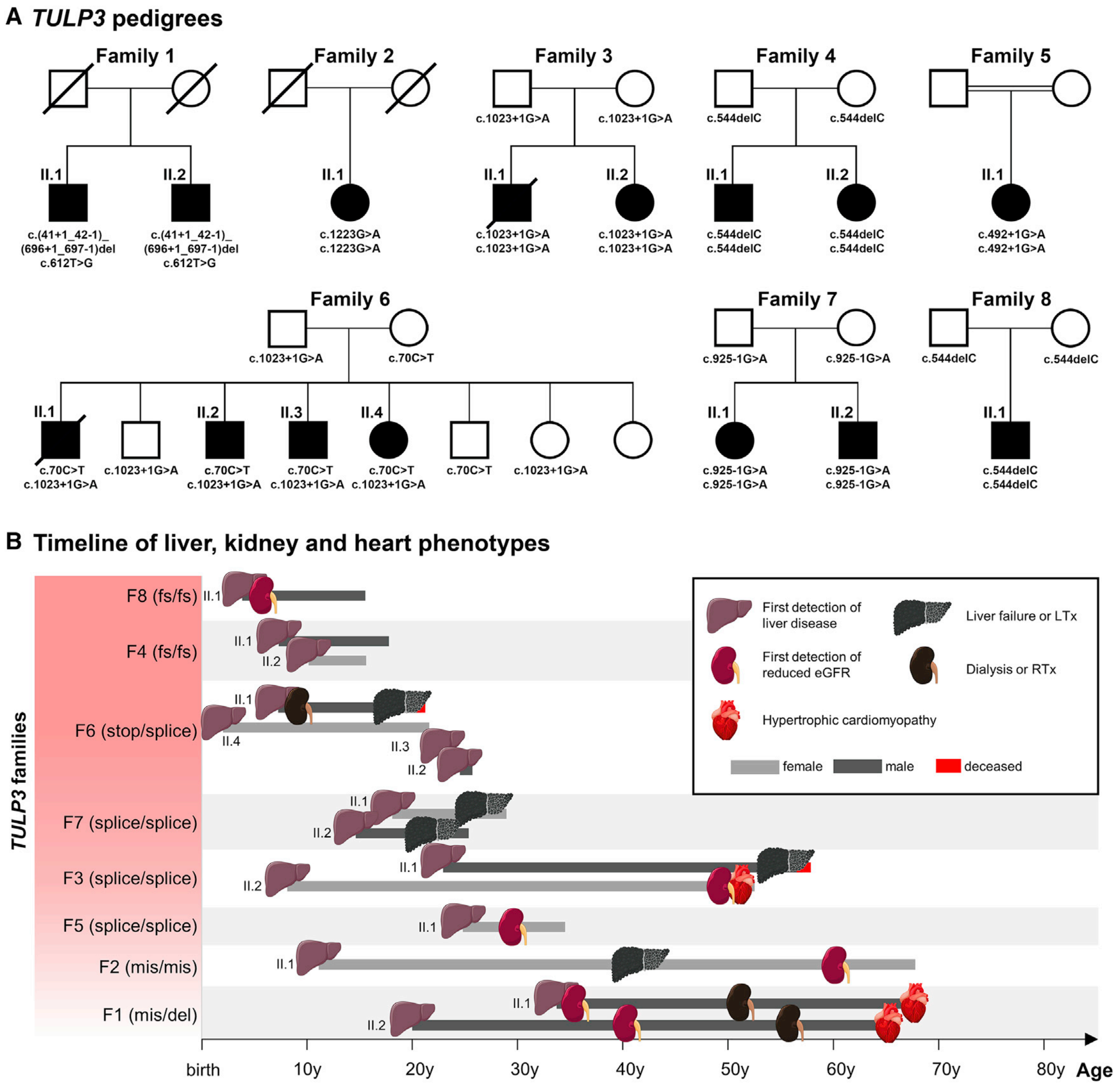

Figure 1. Identification of variants in *TULP3* as a cause of progressive organ fibrosis in 15 affected individuals from eight unrelated families

(A) Pedigrees for each of the eight reported families. Different massively parallel sequencing (MPS) approaches and GeneMatcher were used for identification of *TULP3* variants. Affected individuals (black symbols) presented with progressive fibrotic liver disease and variable kidney and heart disease (full details in Table 1). *TULP3* genetic changes are shown below symbols of individuals. Notably, the clinical features segregate with bi-allelic mutations in *TULP3* (homozygous or compound heterozygous), implicating variants in *TULP3* in autosomal recessive progressive fibrotic disease.

(B) Graphical timeline showing the age of identification of liver, kidney, and heart phenotypes for all affected individuals (full details in Table 1). All affected individuals presented with complications of liver disease, and initial disease manifestations ranged from 2 to 33 years of age. Signs of chronic kidney disease were predominantly observed starting at the 2nd decade. End-stage kidney disease was observed in three affected individuals at the age of 7, 51, and 55 years (family 6 [II.1] and family 1 [II.1 and II.2], respectively). Three affected individuals were affected by hypertrophic non-obstructive cardiomyopathy (HNCM) in their 6th and 7th decades of life (family 3 [II.2] and family 1 [II.1 and II.2], respectively). eGFR, estimated glomerular filtration rate; LTx, liver transplantation; RTx, renal transplantation; y, years.

tomography imaging demonstrates liver enlargement with inhomogeneous parenchyma and secondary signs of portal hypertension in affected individuals, but no liver cysts were seen, distinguishing this disease from other cystic kidney and liver disorders (Figure 2A).

Histopathological stains on liver biopsy or liver explants from nine different individuals from five unrelated families were reviewed (Figure 2B and Table S2). Histological evaluation of liver biopsy samples showed a paucicellular portal fibrosis, which was bridging with or without architectural distortion or established cirrhosis. Most cases revealed only a minimal portal inflammatory infiltrate and a moderate unspecific ductular reaction. The explant liver in family 2 (II.1) demonstrated biliary type fibrosis without evidence of an interrupted circular arrangement of ducts, which would be characteristic for ductal plate malformation in the setting of congenital hepatic fibrosis. The explant liver from family 6 (II.1) showed a non-specific

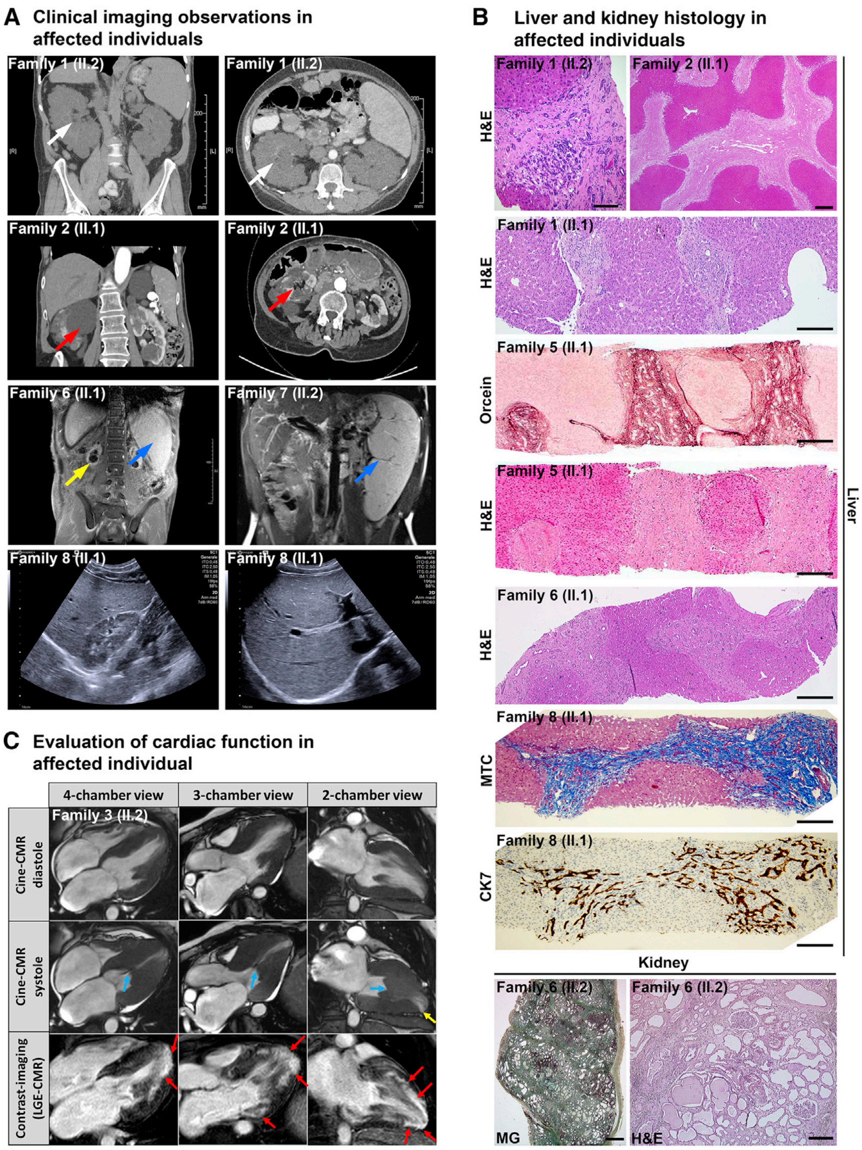

Figure 2. Clinical imaging and histopathological evaluation of affected individuals

(A) Imaging data from affected individuals. A computed tomography (CT) of an individual from family 1 (II.2) showing small renal cysts (white arrow). CT image of family 2 (II.1) showing large renal cysts (red arrow); note: transplant liver. Magnetic resonance imaging (MRI) scan from an individual from family 6 (II.1) shows splenomegaly (blue arrow) and end-stage atrophic fibrocystic kidneys (yellow arrow). MRI of affected individuals from family 7 (II.2) showing splenomegaly (blue arrow). Abdominal ultrasound pictures from family 8 (II.1) show renal microcysts with increased cortical echogenicity and hepatomegaly with increased tissue echogenicity.

(B) Histopathology phenotypes observed in selected affected individuals. Family 1 (II.2), histological analyses of liver (H&E staining) showing hepatic fibrosis-dependent expansion of portal spaces and only little inflammatory infiltrate (scale bar, 100 μm). Family 2 (II.1) and family 1 (II.1), histological analyses of liver (H&E staining) compatible with hepatic cirrhosis (2 [II.1]: scale bar, 500 μm; 1 [II.1]: scale bar, 200 μm). Family 5 (II.1), orcein and H&E stains highlighting broad fibrous septa crossing the biopsy (scale bar, 200 μm). Family 6 (II.1), fibrosis pattern compatible with either biliary type cirrhosis or congenital hepatic fibrosis-like pattern but without ductal plate malformation, with only minimal portal inflammatory infiltrate and a moderate unspecific ductular reaction (scale bar, 200 μm). Family 8 (II.1), architectural disturbance of the hepatic parenchyma due to portal bridging fibrosis as assessed by Masson‘s trichome staining (MTC). CK7 (cytokeratin 7) staining shows proliferation of dysmorphic bile ductules (scale bar, 200 μm). Family 6 (II.2), kidney sections: MG (May-Gruenwald) staining (scale bar, 1,000 μm), and H&E staining (scale bar, 100 μm) showing renal fibrosis and kidney cysts.

(C) Cardiovascular magnetic resonance (CMR) images of the affected individuals from family 3 (II.2) that were acquired at the age of 53 years. The upper panel shows cine-CMR images obtained in diastole. Middle panel illustrates corresponding systolic cine-images. CMR reveals a severe, concentric, septally pronounced pattern of LV hypertrophy with additional intraventricular obstruction due to a kissing-wall phenomenon (blue arrows) and subsequent apical wall thinning with regional akinesia (yellow arrow). Corresponding late-gadolinium-enhancement (LGE) images are illustrated in the lower panels. Hyperintense areas are indicative of myocardial fibrosis (red arrows). A progressive and extensive non-ischemic pattern of LGE was depicted not only at the right ventricular (RV) insertion points (basal anteroseptal and inferoseptal LV wall) but also in the mid- to apical LV free wall and whole LV apex. This extensive and peculiar pattern of myocardial fibrosis cannot be explained by classical hypertrophic cardiomyopathy (HCM) but in contrast indicates a systemic disease with cardiac involvement.

cirrhotic pattern. Liver cysts or von-Meyenburg complexes were not detected in the samples available for histological evaluation. In conclusion, none of the samples from affected individuals showed the typical histological pattern of congenital hepatic fibrosis that has been described in association with ARPKD[30] (Table S2).

Kidney involvement was detected in all but one family (family 4, which has the youngest affected individuals of our cohort at 16 and 18 years). Kidney disease was heterogeneous and chronic kidney disease was usually detected later in life (3rd–6th decade) (Figure 1B and Table 1). The most common ultrasonographic presentation was hyperechogenic kidneys with reduced corticomedullary differentiation or multiple kidney cysts (Figure 2A). A kidney biopsy (family 6 [II.2]) showed widespread interstitial fibrosis with tubular dilatations (Figure 2B and Table 1).

Three adult individuals from two unrelated families presented with morphological signs of hypertrophic nonobstructive cardiomyopathy (HNCM) detected in their 6th or 7th decade of life. A cardiac biopsy of affected

Table 1. Clinical observations in affected individuals with bi-allelic *TULP3* variants

Family	Genomic variation (GRCh38)	*TULP3* nucleotide/ amino acid change (GenBank: NM_003324.5)	Affected individual ID, origin, sex, age	Liver phenotype	Kidney phenotype	Cardiac phenotype	Malignancy	Other clinical features
1	allele 1 12: (3000155_3018694)_ (3040407_3042583)	c.(41+1_42−1)_ (696+1_697−1)del	II.1, German, M, 68 years	HSM, inhomogeneous liver parenchyma, portal HTN, elevated liver enzymes (22 years), cirrhosis	cystic kidneys (detected 33 years), HD (51 years), RTx (52 years)	cardiac MRI: hypertrophic non-obstructive cardiomyopathy (68 years)	basal cell carcinoma forehead, squamous cell carcinoma right eyebrow (59 years)	–
	allele 2 12:2931156: T:G rs547315819	c.612T>G (p.Cys204Trp)	II.2, German, M, 65 years	elevated liver enzymes (20 years), HSM, inhomogeneous liver parenchyma, portal HTN, variceal banding, hepatic encephalopathy, cirrhosis, awaiting LTx (since age 57 years)	cystic kidneys (detected at 43 years), HD (55 years), on waiting list for RTx (since age 57 years)	cardiac MRI: hypertrophic non-obstructive cardiomyopathy (65 years)	NSCLC adenocarcinoma (62 years)	arterial HTN (54 years), chronic pancreatitis, multiple pancreatic cysts
2	allele 1/2 12:2939338:G:A rs761172007	c.1223G>A (p.Arg408His)	II.1, British, F, 68 years	cholestasis/jaundice, portal HTN, GI bleeding (11 years), portovenous shunt (12 years), biliary cirrhosis, LTx (41 years)	multiple cortical and small medullary renal cysts, enlarged kidneys, eGFR: 40	TTE normal (41 years)	no	primary infertility, arterial HTN splenic artery aneurysm
3	allele 1/2 12:2937730: G:A rs202037575	c.1023+1G>A	II.1, German, M, 57 years (deceased)	elevated liver enzymes (25 years), portal HTN (31 years), esophageal bleeding (33 years), portacaval shunt, hepatic encephalopathy, and death due to liver failure (57 years)	not known	not examined	no	–
			II.2, German, F, 53 years	elevated liver enzymes in childhood, HSM, thrombocytopenia, bridging fibrosis (38 years)	normal-sized kidneys with hyperechogenic parenchyma and reduced CMD (51 years), eGFR: 52 (53 years)	cardiac MRI: hypertrophic non-obstructive cardiomyopathy (53 years); biopsy—moderate chronic myocardial damage, diffuse interstitial fibrosis of myocardium, and degeneration of myocardial cells	no	–

(*Continued on next page*)

Table 1. *Continued*

Family	Genomic variation (GRCh38)	*TULP3* nucleotide/ amino acid change (GenBank: NM_003324.5)	Affected individual ID, origin, sex, age	Liver phenotype	Kidney phenotype	Cardiac phenotype	Malignancy	Other clinical features
4	allele 1/2 12:2931087:C: rs924744512	c.544delC (p.Leu182TrpfsTer4)	II.1, Macedonian, M, 18 years	elevated liver enzymes in childhood, cholestatic hepatopathy, HSM, portal HTN with hypersplenism, variceal banding, increased elastography values (14 years)	no	TTE and ECG normal (14 years)	no	–
			II.2, Macedonian, F, 16 years	abdominal pain in childhood, HSM, portal HTN with hypersplenism (+pancytopenia), variceal banding (12 years)	no	TTE and ECG normal (13 years)	no	–
5	allele 1/2 12:2930346:G:A rs145289428	c.492+1G>A	II.1, Pakistani, F, 34 years	cholestasis, gestational pruritus (26 years), portal HTN, bridging fibrosis	eGFR: 50, normal kidney USS	TTE normal (34 years)	no	arterial HTN, obesity, Bell's palsy, labyrinthitis, chronic tonsillitis
6	allele 1 12:2937730:G:A rs202037575	c.1023+1G>A	II.1, German, M, 21 years (deceased)	elevated liver enzymes in childhood. HSM with inhomogeneous liver parenchyma, ascites, cirrhosis, LTx (21 years), deceased age 21 years (post Tx complications)	normal-sized kidneys with hyperechogenic parenchyma and reduced CMD, HD (7 years), 1st RTx (8 years), 2nd RTx (15 years)	TTE normal (21 years)	no	–
	allele 2 12:2909557:C:T rs201665307	c.70C>T (p.Arg24Ter)	II.2, German, M, 26 years	elevated liver enzymes (19 years), HSM, cirrhosis	enlarged hyperechogenic kidneys, eGFR > 90; biopsy—diffuse interstitial fibrosis, corticomedullary scarring, tubular dilatations	TTE normal (26 years)	no	–
			II.3, German, M, 24 years	elevated liver enzymes (20 years), HSM, bridging fibrosis	enlarged hyperechogenic kidneys, eGFR > 90	TTE normal (24 years)	no	–
			II.4, German, F, 22 years	elevated liver enzymes in infancy, HSM, esophageal variceal bleeding, TIPS (20 years), bridging fibrosis with architectural distortion, awaiting LTx	renal parenchymal hyperechogenicity, reduced CMD, right kidney small isolated 2 mm cyst (21years)	TTE normal (20 years)	no	–

(*Continued on next page*)

Table 1. *Continued*

Family	Genomic variation (GRCh38)	*TULP3* nucleotide/amino acid change (GenBank: NM_003324.5)	Affected individual ID, origin, sex, age	Liver phenotype	Kidney phenotype	Cardiac phenotype	Malignancy	Other clinical features
7	allele 1/2 12:2937630:G:A rs761012512	c.925−1G>A	II.1, Northern European, F, 29 years	elevated liver enzymes in early adulthood, portal HTN with esophageal varices (25 years), cirrhosis with mild lobular and portal inflammation, LTx (27 years)	non-enlarged cystic kidneys, eGFR > 90	TTE normal (27 years)	no	acute pancreatitis (19 years)
			II.2, Northern European, M, 26 years	elevated liver enzymes in childhood, decompensated portal HTN (18 years), TIPS (20 years), cirrhosis, LTx (21 years)	enlarged kidneys, right kidney 2 cysts (15 mm), eGFR > 60	TTE normal (20 years)	hepatocellular carcinoma (20 years)	–
8	allele 1/2 12:2931087:C: rs924744512	c.544delC (p.Leu182TrpfsTer4)	II.1, Italian, M, 16 years	cholestasis and jaundice (onset 4 years), portal HTN, bridging fibrosis with architectural distortion	non-enlarged kidneys with cortical and medullary microcysts with increased cortical echogenicity, eGFR: 86	TTE normal (16 years)	no	–

Affected individuals present with fibrotic liver features (bridging fibrosis, cirrhosis), variable fibrocystic kidney disease, and hypertrophic non-obstructive cardiomyopathy in older affected individuals (6th to 7th decade). Abbreviations: CHF, congenital hepatic fibrosis; CMD, corticomedullary differentiation; ECG, electrocardiogram; eGFR, estimated GFR (CKD-EPI formula) mL/min/1.73 m^2; F, female; GI, gastrointestinal; HSM, hepatosplenomegaly; HTN, hypertension; LTx, liver transplant; M, male; NSCLC, non-small cell lung cancer; RTx, renal transplant; TIPS, transjugular intrahepatic portosystemic shunt; TTE, transthoracic echocardiogram; USS, ultrasound scan.

individual II.2 from family 3 showed evidence of diffuse interstitial fibrosis of the myocardium and degeneration of myocardial cells (Table 1). In the same individual, MRI of the heart revealed left ventricular hypertrophy and systemic myocardial fibrosis (Figure 2C). We screened the other, mostly younger affected individuals with transthoracic echocardiogram, none of whom showed features of HNCM (Table 1).

Inactivation of zebrafish *tulp3* causes adult liver and kidney disease

We investigated the functions of TULP3 by using the zebrafish as a vertebrate model organism. Zebrafish Tulp3 is closely related to its human counterpart (Figure 3A). Semiquantitative RT-PCR analysis performed on zebrafish embryos indicated *tulp3* expression throughout embryogenesis with peak levels during the first 24 h post fertilization (hpf) and at 5 days post fertilization (dpf). These data are consistent with publicly available zebrafish developmental RNA sequencing data (e.g., EBI Expression Atlas[31]) showing *tulp3* expression at all studied time points (zygote to 5 dpf) with highest relative levels at late gastrulation/beginning somitogenesis (6–10 hpf) and later stages (4–5 dpf), suggesting continued expression of *tulp3* after embryonic development. Analysis on a series of adult zebrafish tissues indicated highest expression (relative to housekeeping gene expression) in the gonads, the brain, as well as the kidney, liver, and heart (Figure 3B).

We generated a KO model of zebrafish *tulp3* through a CRISPR-Cas9-mediated 5 bp deletion that induces a stop codon in exon 5 of *tulp3* (Figure 3A). Semiquantitative RT-PCR and qPCR on cDNA from maternal-zygotic (MZ) *tulp3* mutants and control clutchmates confirmed the 5 bp deletion and premature stop codon and demonstrated significantly reduced *tulp3* expression that most likely results from nonsense-mediated decay (Figure 3A). Given the late onset of clinical features in reported affected individuals, we evaluated the effect of Tulp3 loss of function in adult zebrafish, which survive to adulthood, in contrast to comparable murine models of TULP3 loss of function.[26]

Due to the prominent liver, kidney, and heart phenotypes observed in affected individuals harboring deleterious *TULP3* variants, we analyzed tissue sections from adult (18 months old) homozygous *tulp3* zebrafish mutant (*tulp3 m/m*) liver, kidney, and heart. We then compared these to control zebrafish (*tulp3* +/+) derived from the same incross (clutchmates). In the livers of adult *tulp3 m/m,* we observed significant cytoplasmic clearing of the hepatocytes indicating steatosis (Figures 3C and 3F). Adult *tulp3 m/m* also develop a mild cystic kidney disease with cysts in both proximal and distal tubules (Figure 3D). Cystic index scoring reveals a mild but consistent cystic kidney phenotype in these animals compared to *tulp3* +/+ animals (Figure 3F). Evaluation of heart tissue from adult *tulp3 m/m* zebrafish mutants found no aberrant morphological features, and histological examination

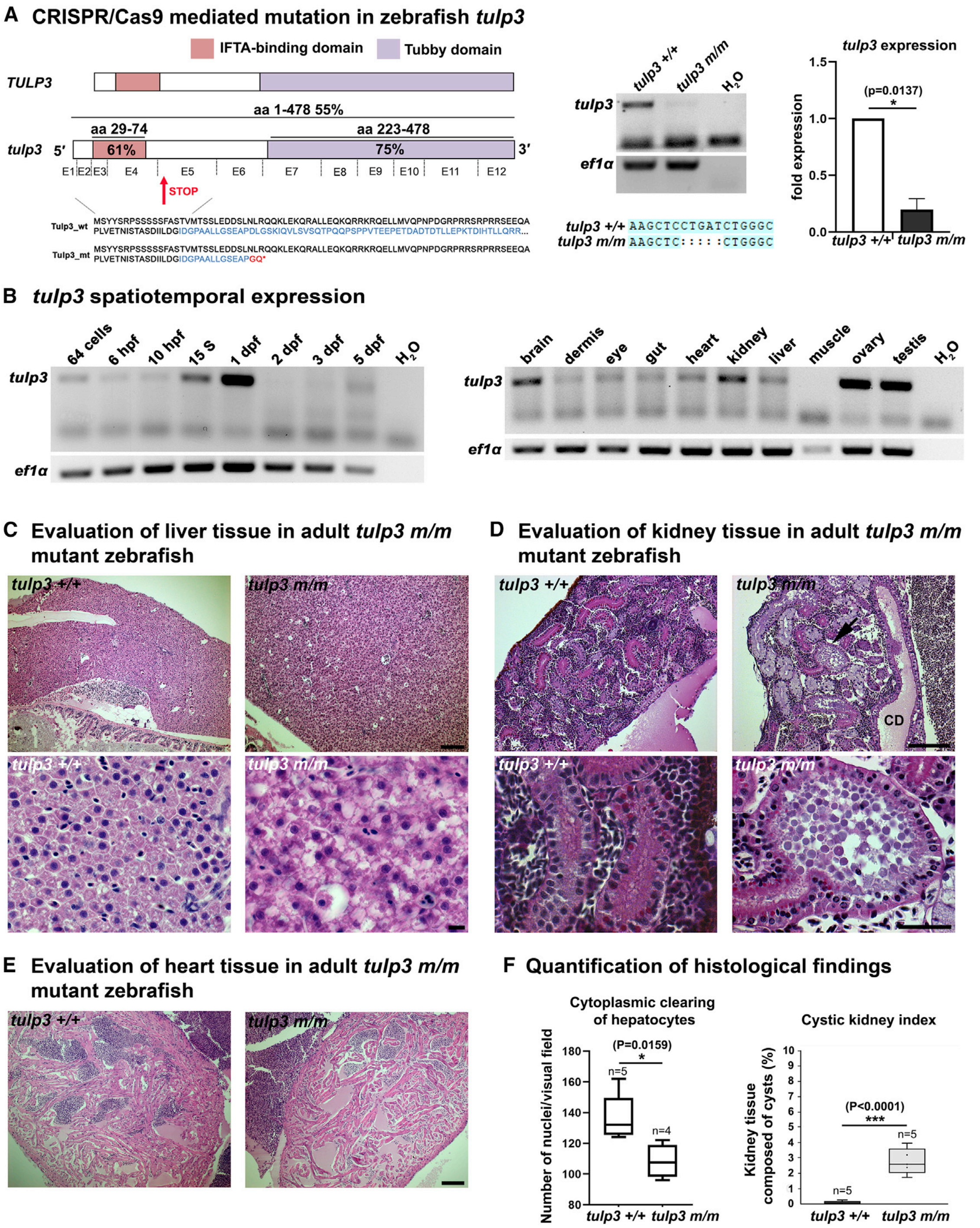

Figure 3. Inactivation of zebrafish *tulp3* causes adult liver and kidney disease

(A) Left: schematic showing conservation between human TULP3 and zebrafish Tulp3. Amino acid sequences aligned with the Clustal Omega MView tool. Tulp3 shows 54.5% overall homology with TULP3 particularly within the IFT-A-binding (red) and Tubby (purple) domains (61% and 75%, respectively). The lower part of the figure shows mapping of the exons onto Tulp3 protein structure and shows the position of CRISPR-Cas9-mediated deletion in exon 5 leading to an early stop codon, p.Asp106Glyfs2Ter. Right: Semiquantitative RT-PCR and qPCR revealed a strong reduction in *tulp3* mRNA expression in MZ*tulp3* mutant embryos compared to the respective control indicating a functional *tulp3* null mutation. Sanger sequencing confirmed the 5 bp deletion in exon 5. Error bar represents SEM; *$p < 0.05$ (one-sample t test).

(B) Semiquantitative RT-PCR analysis of *tulp3* expression during development (left) and in isolated adult zebrafish tissues (right). *tulp3* is expressed in various adult tissues, including liver, kidney, and heart; *ef1α* was used as housekeeping gene. Hpf, h post fertilization; dpf, days post fertilization; 15 S, 15-somites stage.

(C) Histological analyses of liver samples (H&E stain) isolated from adult (18 months) *tulp3* +/+ wild-type and *tulp3 m/m* mutant zebrafish clutchmates. Liver sections of *tulp3 m/m* zebrafish show cytoplasmic clearing of hepatocytes indicating steatosis (scale bar upper panel, 100 μm; scale bar lower panel, 10 μm).

(legend continued on next page)

found no indication of fibrosis or underlying cellular disruptions (Figure 3E).

Disrupted ciliary cargo composition in cells derived from affected individuals

To characterize the molecular and cellular consequences of *TULP3* mutations in affected individuals, we obtained primary, non-transformed human URECs (family 2 [II.1]) as well as skin fibroblasts (family 3 [II.2] and family 6 [II.4]) and age- and sex-matched controls from non-affected individuals.

Most of the detected genetic variants were predicted to have a high (disruptive) impact on protein function (Table S1). Furthermore, immunofluorescence analysis revealed near-complete loss of ciliary TULP3 localization in URECs from the family 2 affected individual with a homozygous missense variant (Figure S4A). We investigated the consequences of *TULP3* mutations on ciliary composition by assessing the levels of TULP3 cargo proteins GPR161, ARL13B, and INPP5E in URECs and fibroblast cells derived from affected individuals. In primary cells from family 2 (II.1) and family 6 (II.4), we observed a strong reduction in ciliary GPR161, a negative regulator of sonic hedgehog (SHH) signaling.[32] ARL13B intensities were reduced in these affected individuals' cells and INPP5E was nearly undetectable, an observation possibly secondary to reduced ARL13B levels (Figures 4A and 4B). Notably, localization assays in fibroblasts from family 3 (II.2) (c.1023+1G>A) showed less severe localization defects; only GPR161 displayed defective trafficking, consistent with the in-frame splicing defect caused by this mutation (Figures S3E–S3H and Figures S4B–S4E). These results are in line with previous experiments mostly obtained in *Tulp3*-deficient models[14–16] and suggest TULP3 loss of function as a shared disease mechanism in our affected individuals. In conclusion, using URECs and fibroblast cells derived from affected individuals, we demonstrate a functional impact of the identified genetic variants in *TULP3* resulting in disruption of ciliary composition, including proteins previously associated with human ciliopathies (MIM: 213300, 612291).[33]

TULP3 interacts with DNA damage repair protein and key fibrosis modulator SIRT1

To identify potential TULP3 interaction partners, we performed mass spectroscopy on tandem affinity purified HEK293T whole-cell lysate. The TULP3 interaction network suggests an association with several core DDR elements, including DDB1 and TP53 (Figure S5). Among the identified protein interactions was the recently reported TULP3 interaction partner SIRT1,[34] a class III histone deacetylase that has broad reaching roles as a regulator of transcription and DDR by mediating deacetylation of TP53 and several histones.[35–37] We confirmed the interaction between TULP3 and SIRT1 through co-immunoprecipitation in HEK293T cells (Figure 4C). TULP3 has been shown to locate both to the plasma membrane/primary cilium as well as to the nucleus,[13,17] where SIRT1 is predominantly expressed.[37] Given the link between TP53 and DDR, we next assessed levels of γH2AX, a marker of DNA damage, in URECs (family 2 [II.1]) and fibroblasts (family 3 [II.2]) derived from affected individuals and found significant increase in γH2AX nuclear staining intensity and punctae, suggesting increased DNA damage in these cells (Figure 4D and Figure S4F). Notably, SIRT1 has also been identified as a modulator of organ fibrosis most likely linked to its role as modulator of TGF-β signaling.[38,39]

Profibrotic WNT-signaling-, TGF-β-signaling-, and cardiomyopathy-related gene expression is significantly increased in *TULP3* cells derived from affected individuals

To gain further insight into the pathophysiology underlying *TULP3*-related disease phenotypes, we performed RNA sequencing in fibroblast cells from one of our affected individuals harboring the homozygous canonical *TULP3* splicing variant c.1023+1G>A (family 3 [II.2]). In accordance with the clinical phenotype, gene clusters for hypertrophic cardiomyopathy and WNT and TGF-β signaling were significantly dysregulated (Figure 5 and Figure S6A). Upregulation of TGF-β effectors *SMAD3* (MIM: 603109) and direct targets of canonical TGF-β signaling *SERPIN1* (MIM: 173360), as well as downregulation of the TGF-β pathway inhibitor *SMAD7* (MIM: 602932), suggest activation of the TGF-β pathway[40] (Figure S6A). Similarly, upregulation of *LEF1* (MIM: 152245) and *TCF7* (MIM: 189908), principle WNT pathway effectors, indicates activation of the WNT signaling pathway[41] (Figure 5C). Notably, the WNT-signaling-associated genes include members of both the canonical β-catenin as well as the non-canonical planar cell polarity (PCP) pathways (Figure 5C). Upregulation of TGF-β signaling is noteworthy and could be secondary to disrupted interaction between

(D) Histological analyses of kidney samples (H&E stain) isolated from adult (18 months) *tulp3 +/+* wild-type and *tulp3 m/m* mutant zebrafish clutchmates. Kidney sections of *tulp3 m/m* zebrafish show mild cysts (black arrow), observed in both proximal and distal kidney tubules (scale bar upper panel, 50 μm; scale bar lower panel, 10 μm). CD: collecting duct.
(E) Histological analyses of heart samples (H&E stain) isolated from adult (18 months) *tulp3 +/+* wild-type and *tulp3 m/m* mutant zebrafish clutchmates (scale bar, 100 μm). Microtome sections of the adult zebrafish ventricle. We did not note any fibrotic events or cellular changes in the hearts of these animals at 18 months.
(F) Boxplots for liver and kidney phenotypes observed in *tulp3 m/m* adult zebrafish. For indirect quantification of cytoplasmic clearing, the nuclei of hepatocytes in visual fields of *tulp3 m/m* compared to *tulp3 +/+* clutchmates were analyzed, showing a significant reduction in nuclei in *tulp3 m/m* zebrafish. *$p = 0.0159$ (two-tailed, unpaired Student's t test). An increased cystic index score was observed in *tulp3 m/m* zebrafish kidney compared to *tulp3 +/+* clutchmates ($n = 5$). ***$p < 0.001$ (two-tailed, unpaired Student's t test).

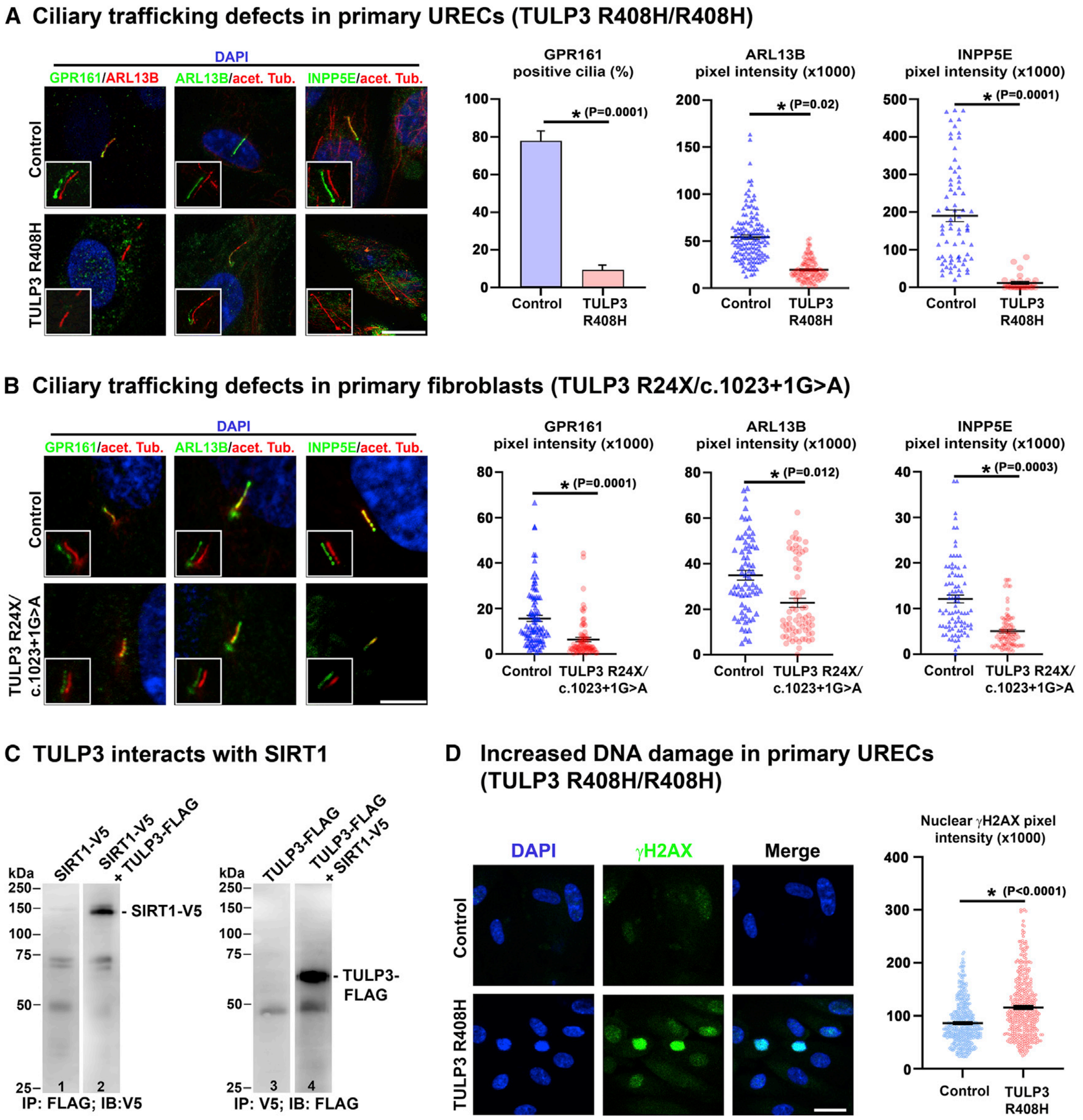

Figure 4. ***TULP3*-affected individuals' cells show defects in ciliary composition and increased DNA damage**
(A) Representative confocal micrographs assessing the effect of *TULP3* mutations on ciliary composition in urine-derived renal epithelial cells (URECs) (family 2 [II.1], c.1223G>A [p.Arg408His]) and compared to age- and sex-matched control cells. Serum-starved cells were stained with antibodies against acetylated tubulin, GPR161, ARL13B, and INPP5E. Cell nuclei were counterstained with DAPI. Affected-individual-derived URECs showed significantly reduced ciliary localization of GPR161, ARL13B, and INPP5E. Left panel: staining of control and affected individual (family 2 [II.1)]) URECs (scale bar, 5 μm). Right panel: corresponding quantification of GPR161-positive cilia and ciliary signal intensities of ARL13B and INPP5E.
(B) Representative confocal micrographs assessing the effect of *TULP3* mutations on ciliary composition in fibroblasts derived from an affected individual (family 6 [II.4], c.1023+1G>A/c.70C>T [p.Arg24Ter]). Compared to age- and sex-matched control cells affected-individual-derived fibroblasts showed significantly reduced ciliary localization of GPR161, ARL13B, and INPP5E (scale bar, 5 μm). Right panel: corresponding quantification of ciliary signal intensity levels. For (A) and (B): *$p < 0.05$; error bars show SEM (two-tailed, unpaired Student's t test performed on the means of three independent experiments).
(C) Interaction of human TULP3 with SIRT1. FLAG-tagged full-length human TULP3 was co-transfected with V5-tagged full-length SIRT1 in HEK293T cells. SIRT1 was detected in TULP3 precipitates (FLAG-M2 beads for immunoprecipitation [IP], anti-V5 for immunoblotting [IB]), and correspondingly TULP3 was detected in SIRT1 precipitates (V5 beads for IP, anti-FLAG for IB); kDa, kilodalton.
(D) Increased DNA damage response (DDR) in affected-individual-derived URECs (family 2 [II.1]). γH2AX was used as an immunocytochemical marker of DDR and intensity of nuclear signal was compared in the affected individual and sex- and age-matched control URECs at the same passage number (scale bar, 20 μm). A significant increase in γH2AX signal was detected in affected-individual-derived URECs compared to control cells. *$p < 0.05$; error bars represent SEM (two-tailed, unpaired Student's t test on three independent experiments).

TULP3 and SIRT1 in affected-individuals-derived cells (Figure S6A). The SHH pathway is notably absent from our transcriptional analysis. Targeted evaluation of SHH pathway components in family 3 (II.2) fibroblasts by qPCR confirmed no deregulation of key SHH pathway components in this affected individual's cells

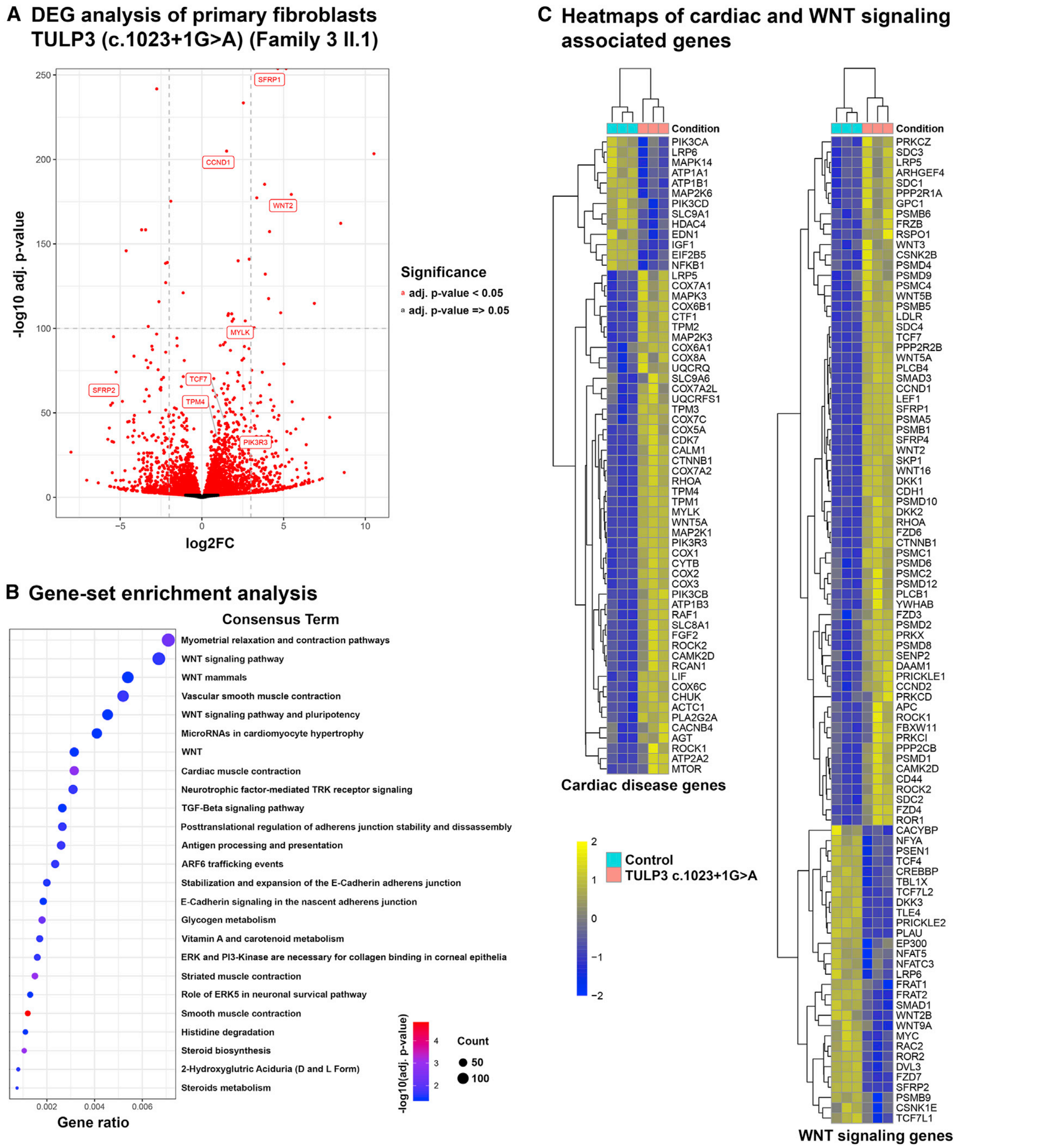

Figure 5. Increased levels of WNT-signaling-, TGF-β-signaling-, and cardiomyopathy-associated genes in *TULP3*-affected individual's cells

(A) RNA sequencing results performed in healthy control and fibroblasts derived from affected individual (family 3 [II.2]). Differentially regulated genes (DEGs) were identified by the gene set analysis method GAGE (generally applicable gene-set enrichment). The most dysregulated genes associated with WNT signaling or cardiac disease are labeled.

(B) Enrichment analysis of signaling pathways. Pathways were considered significant with adjusted p values (Benjamini-Hochberg) $p < 0.05$. Among these pathways, significantly dysregulated genes associated with WNT signaling, TGF-β signaling, and cardiac muscle contraction/microRNAs in cardiomyocyte hypertrophy were identified.

(C) Gene expression heatmaps for differentially regulated genes from the indicated GSEA terms "cardiac muscle contraction/microRNAs in cardiomyocyte hypertrophy" (left) and WNT signaling (right). Each column represents an individual sample from control or affected-individual-derived cells.

(Figure S6B). We observed only few downregulated signaling processes; significant gene reduction was mainly associated with functions in cell cycle, ribosome, and circadian rhythm (Figure S6C).

Discussion

We describe bi-allelic variants in *TULP3* in 15 individuals from eight unrelated families and establish by further

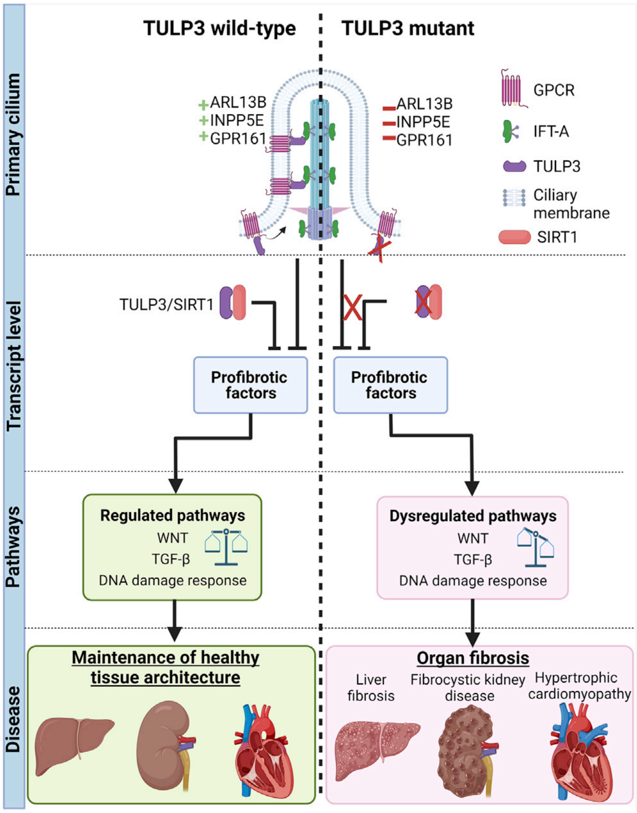

Figure 6. Converging pathomechanisms of fibrosis in *TULP3*-affected individuals

A schematic of the hypothesized disease mechanism of fibrosis in *TULP3*-affected individuals. Shown are cartoons of the primary cilium with axoneme, intraflagellar transport machinery and membrane bound receptors and ciliary proteins, transcriptional changes, affected pathways, and clinical outcomes. On the left is the *TULP3* wild-type healthy state versus *TULP3* mutant disease state (right), separated by the vertical dotted line. At the cilium, *TULP3* mutations results in defective trafficking of ARL13B, INPP5E, and GPR161 (and likely other GPCRs). Disrupted ciliary composition leads to a loss of regulatory signals from the cilium, causing dysregulated profibrotic WNT and TGF-β signaling pathways, either directly or indirectly. Green crosses represent correctly localized receptors and proteins. Red crosses represent loss of receptors or proteins from the cilium. We demonstrate that TULP3 interacts directly with SIRT1, a key regulator of fibrosis. We hypothesize that disruptive mutations in TULP3 lead to reduced SIRT1 modulation of profibrotic signaling pathways through a yet unknown mechanism. We propose that TULP3 is a key regulator of fibrosis that functions at multiple levels. Disruption of these regulatory mechanisms converge and results in chronic activation of profibrotic signaling cascades leading to progressive fibrosis in *TULP3*-affected individuals. Black arrows represent activation, bar headed lines represent inhibition, and red Xs represent loss of elements from the network. Created with https://biorender.com/.

in vitro and *in vivo* experiments variants in *TULP3* as a monogenic cause for a clinically distinct disorder of progressive degenerative liver fibrosis with variable fibrocystic kidney disease and hypertrophic cardiomyopathy. Postnatal disease onset was variable, ranging from childhood to adulthood. Therefore, our study highlights the increasing importance of broad genetic sequencing for the discovery of autosomal recessively inherited disorders, even among adults affected with a primarily fibrotic disease affecting the liver, kidney, and heart. We hypothesize that early detection of individuals at high risk for progression to end-stage liver and kidney disease could lead to improved clinical management, focusing on preservation of renal function and screening for complications of cirrhosis, such as esophageal varices and hepatocellular carcinoma, which occurred in several young adults in our study. The progressing course of disease caused by *TULP3* variants demonstrates the importance of periodic liver, kidney, and heart screening tests to delay organ dysfunction through early therapeutic actions.

Organ fibrosis is clinically and genetically heterogeneous and not well understood. So far, only a few genes representing monogenic causes for liver fibrosis are known.[42] Defects in genes resulting in alterations of the primary cilium are linked to hepatorenal fibrocystic disease with ductal plate malformation and periportal fibrosis.[30] Notably, liver biopsies obtained from several affected individuals in this study are different from typical ductal plate malformation, suggesting a distinct clinical and histopathological phenotype.

We observe considerable variability in disease onset and expression in the affected individuals reported here. We did not identify additional, predicted functional variants in genes potentially interacting with *TULP3* or the identified signaling pathways. Notably, we do observe an apparent association between the age of disease onset and the predicted effect of the *TULP3* variants, i.e., the single individual carrying two missense variants had a milder disease course compared to individuals with bi-allelic frameshifting variants (Figure 1B). Similarly, family 3 that is bi-allelic for the likely hypomorph frame-preserving splice variant c.1023+1G>A seemed to present a milder clinical course than family 6 where this hypomorph variant was associated with an early nonsense variant. Given the small numbers, further studies are required to better delineate a possible genotype-phenotype correlation.

Among the six families with homozygous variants in *TULP3*, only one was knowingly consanguineous. The identified *TULP3* variants are all rare, and none of them are detected at the homozygote state in gnomAD. Although none of the remaining five families presented obvious intrafamilial relationships, we noticed the recurrence of two variants: c.544delC in two Mediterranean families (Italian and Macedonian) and c.1023+1G>A in two German pedigrees. This observation may suggest a shared ancestor in these families. In line with a potential common distant parental ancestor, we also detected a stretch of homozygosity around *TULP3* in the proband from family 2, albeit shorter compared with the proband from the consanguineous family 5 (~0.3 Mb versus 6 Mb, data not shown).

We modeled TULP3 loss of function in *tulp3* KO zebrafish. In adult Tulp3-deficient zebrafish, we observed fibrocystic disease including liver fibrosis and cystic kidney

disease, which mirrors the clinical presentation of affected individuals with deleterious *TULP3* mutations. *TULP3*-affected individuals develop hypertrophic cardiomyopathy that appears to be age related. Evaluation of heart tissue from adult *tulp3 m/m* zebrafish mutants did not reveal any underlying pathology. Given the 3–5 year lifespan of zebrafish,[43] at 18 months of age it is likely that these animals may not have been old enough to develop the respective age-related cardiac phenotypes.

RNA sequencing demonstrates significant dysregulation of profibrotic pathways in line with the clinical course of our affected individuals with *TULP3* variants. In contrast to the described roles of TULP3 as a negative regulator of SHH,[32] transcription-level expression data from cells derived from affected individuals indicate significant upregulation of key effectors of both WNT and canonical TGF-β signaling pathways, suggesting that both WNT and canonical TGF-β signaling are activated in these affected individuals. Notably, the primary cilium appears to be a signaling hub for canonical TGF-β.[44] Furthermore, murine models of cardiac fibrosis require primary cilia to propagate TGF-β-mediated fibrosis.[45]

SHH, WNT, and TGF-β signaling are strongly interrelated mediators of fibrosis with well-established functions at the primary cilium. The SHH pathway has been shown to direct differentiation of myofibroblasts through interaction with both TGF-β and WNT signaling pathways.[46,47] TULP3 functions as an adaptor for a subset of membrane-bound proteins destined for the cilium and therefore is in a prime position to provide fine control of integrated signaling pathways. Notably, while TULP3 is a well-established regulator of the SHH pathway, we do not observe any deregulation of the SHH pathway in our transcriptional RNA-sequencing analysis or through qPCR analysis of SHH components. The absence of SHH signaling is in line with the clinical presentation where typical manifestations such as laterality defects or polydactyly were not observed.

TULP3 also displays nuclear localization and translocates from the cilium to the nucleus upon GPCR activation.[17] While the functions of TULP3 at the nucleus are unknown, we identified potential interaction with DDR pathway proteins and observe increased DDR in our cells derived from affected individuals. Notably, aberrant DDR has been linked to progressive fibrosis in affected individuals with hypomorphic *CEP164* (MIM: 614848) mutations.[10] Additionally, we confirm a direct interaction between TULP3 and SIRT1, a well-established regulator of TGF-β-mediated organ fibrosis.[38,39] The role that TULP3 plays in either of these pathways is incompletely elucidated and will require further investigation (Figure 6).

Altogether, we describe compelling clinical and experimental data to substantiate variants in *TULP3* as a monogenic cause of progressive degenerative liver fibrosis with variable fibrocystic kidney disease and hypertrophic cardiomyopathy. This disease is a pathophysiologically distinct entity that shares commonalities with other ciliopathies. Expanding our understanding of fibrogenesis and the contribution of ciliopathy-associated genes to diverse phenotypes may open new therapeutic approaches for the treatment of progressive organ fibrosis.

Data and code availability

Primary data from the 100,000 Genomes Project, which are held in a secure research environment, are available to registered users. See https://www.genomicsengland.co.uk/research/academic for further information.

Supplemental information

Supplemental information can be found online at https://doi.org/10.1016/j.ajhg.2022.03.015.

Acknowledgments

E.G.O. is supported by the Swiss National Science Foundation (P2ZHP3_195181 and P500PB_206851) and Kidney Research UK (Paed_RP_001_20180925). E.D., A.F., N.B., G.R., and T.E. are employees of the Medizinische Genetik Mainz. S.J.R. is funded by a Versus Arthritis career development fellowship (22615). J.A.S. is funded by Kidney Research UK, the Northern Counties Kidney Research Fund, and the Medical Research Coucil (CiC award). C.B. receives support from the Deutsche Forschungsgemeinschaft (DFG, German Research Foundation) (BE 3910/8-1, BE 3910/9-1) and Collaborative Research Center SFB 1453 (project ID: 431984000) and the Federal Ministry of Education and Research (BMBF, 01GM1903I and 01GM1903G). C.B. was also supported by the Collaborative Research Center SFB 1140 (project ID: 246781735). M.B. is supported by the DFG (SFB1453 [project S1], SFB1479 [project ID: 441891347-S1], SFB1160 [Project Z02], and TRR167 [Project Z01]). M.B. and P.M. are supported by the German Federal Ministry of Education and Research by MIRACUM within the Medical Informatics Funding Scheme (FKZ 01ZZ1801B). This research was made possible through access to the data and findings generated by the 100,000 Genomes Project. The 100,000 Genomes Project is managed by Genomics England Limited (a wholly owned company of the Department of Health and Social Care). The 100,000 Genomes Project is funded by the National Institute for Health Research and NHS England. The Wellcome Trust, Cancer Research UK, and the Medical Research Council have also funded research infrastructure. The 100,000 Genomes Project uses data provided by participants and their families and collected by the National Health Service as part of their care and support. A full list of members of the Genomics England Research Consortium and their affiliations are available in the supplemental information.

Declaration of interests

E.D., A.F., N.B., G.R., and T.E. are employees of Medizinische Genetik Mainz. C.B. is an employee and managing director of Medizinische Genetik Mainz and Limbach Genetics GmbH. All other authors declare no competing interests.

Received: December 23, 2021
Accepted: March 22, 2022
Published: April 8, 2022

Web resources

CHOPCHOP target sites for CRISPR-Cas9 mutagenesis, https://chopchop.cbu.uib.no/

ClinVar, https://www.ncbi.nlm.nih.gov/clinvar

Clustal omega, https://www.ebi.ac.uk/Tools/msa/clustalo/

EBI-Expression Atlas, https://www.ebi.ac.uk/gxa/experiments/E-ERAD-475

Ensembl, https://www.ensembl.org/index.html

Ensembl VEP, https://www.ensembl.org/info/docs/tools/vep/index.html

Genomics England 100,000 Genomes Project, https://www.genomicsengland.co.uk/

GnomAD v2.1.1, https://gnomad.broadinstitute.org/

HGMD, http://www.hgmd.cf.ac.uk/ac/index.php

ImageJ, https://imagej.nih.gov/ij/

OMIM, https://omim.org/

ProteinPaint, https://pecan.stjude.cloud/proteinpaint

STRING, https://string-db.org/

The International Mouse Phenotyping Consortium webpage, https://www.mousephenotype.org/

Varsome, https://varsome.com/

References

1. Anthony, P. (1990). Robbins' Pathologic Basis of Disease. J. Clin. Pathol. *43*, 176.
2. Ahmad, A., and Ahmad, R. (2012). Understanding the mechanism of hepatic fibrosis and potential therapeutic approaches. Saudi J. Gastroenterol. *18*, 155–167.
3. Rockey, D.C., Bell, P.D., and Hill, J.A. (2015). Fibrosis–a common pathway to organ injury and failure. N. Engl. J. Med. *372*, 1138–1149.
4. Garcia, C.K. (2018). Insights from human genetic studies of lung and organ fibrosis. J. Clin. Invest. *128*, 36–44.
5. Wynn, T.A. (2004). Fibrotic disease and the T(H)1/T(H)2 paradigm. Nat. Rev. Immunol. *4*, 583–594.
6. Zhou, W., Otto, E.A., Cluckey, A., Airik, R., Hurd, T.W., Chaki, M., Diaz, K., Lach, F.P., Bennett, G.R., Gee, H.Y., et al. (2012). FAN1 mutations cause karyomegalic interstitial nephritis, linking chronic kidney failure to defective DNA damage repair. Nat. Genet. *44*, 910–915.
7. Weidemann, F., Breunig, F., Beer, M., Sandstede, J., Störk, S., Voelker, W., Ertl, G., Knoll, A., Wanner, C., and Strotmann, J.M. (2005). The variation of morphological and functional cardiac manifestation in Fabry disease: potential implications for the time course of the disease. Eur. Heart J. *26*, 1221–1227.
8. Devuyst, O., Olinger, E., Weber, S., Eckardt, K.-U., Kmoch, S., Rampoldi, L., and Bleyer, A.J. (2019). Autosomal dominant tubulointerstitial kidney disease. Nat. Rev. Dis. Primers *5*, 60.
9. Hildebrandt, F., Benzing, T., and Katsanis, N. (2011). Ciliopathies. N. Engl. J. Med. *364*, 1533–1543.
10. Chaki, M., Airik, R., Ghosh, A.K., Giles, R.H., Chen, R., Slaats, G.G., Wang, H., Hurd, T.W., Zhou, W., Cluckey, A., et al. (2012). Exome capture reveals ZNF423 and CEP164 mutations, linking renal ciliopathies to DNA damage response signaling. Cell *150*, 533–548.
11. Dowdle, W.E., Robinson, J.F., Kneist, A., Sirerol-Piquer, M.S., Frints, S.G.M., Corbit, K.C., Zaghloul, N.A., van Lijnschoten, G., Mulders, L., Verver, D.E., et al. (2011). Disruption of a ciliary B9 protein complex causes Meckel syndrome. Am. J. Hum. Genet. *89*, 94–110.
12. Burgmaier, K., Brinker, L., Erger, F., Beck, B.B., Benz, M.R., Bergmann, C., Boyer, O., Collard, L., Dafinger, C., Fila, M., et al. (2021). Refining genotype–phenotype correlations in 304 patients with autosomal recessive polycystic kidney disease and PKHD1 gene variants. Kidney Int. *100*, 650–659.
13. Mukhopadhyay, S., Wen, X., Chih, B., Nelson, C.D., Lane, W.S., Scales, S.J., and Jackson, P.K. (2010). TULP3 bridges the IFT-A complex and membrane phosphoinositides to promote trafficking of G protein-coupled receptors into primary cilia. Genes Dev. *24*, 2180–2193.
14. Hwang, S.-H., Somatilaka, B.N., Badgandi, H., Palicharla, V.R., Walker, R., Shelton, J.M., Qian, F., and Mukhopadhyay, S. (2019). Tulp3 Regulates Renal Cystogenesis by Trafficking of Cystoproteins to Cilia. Curr. Biol. *29*, 790–802.e5.
15. Han, S., Miyoshi, K., Shikada, S., Amano, G., Wang, Y., Yoshimura, T., and Katayama, T. (2019). TULP3 is required for localization of membrane-associated proteins ARL13B and INPP5E to primary cilia. Biochem. Biophys. Res. Commun. *509*, 227–234.
16. Legué, E., and Liem, K.F., Jr. (2019). Tulp3 Is a Ciliary Trafficking Gene that Regulates Polycystic Kidney Disease. Curr. Biol. *29*, 803–812.e5.
17. Santagata, S., Boggon, T.J., Baird, C.L., Gomez, C.A., Zhao, J., Shan, W.S., Myszka, D.G., and Shapiro, L. (2001). G-protein signaling through tubby proteins. Science *292*, 2041–2050.
18. Schrezenmeier, E., Kremerskothen, E., Halleck, F., Staeck, O., Liefeldt, L., Choi, M., Schüler, M., Weber, U., Bachmann, N., Grohmann, M., et al. (2021). The underestimated burden of monogenic kidney disease in adults waitlisted for kidney transplantation. Genet. Med. *23*, 1219–1224.
19. Lu, H., Galeano, M.C.R., Ott, E., Kaeslin, G., Kausalya, P.J., Kramer, C., Ortiz-Brüchle, N., Hilger, N., Metzis, V., Hiersche, M., et al. (2017). Mutations in DZIP1L, which encodes a ciliary-transition-zone protein, cause autosomal recessive polycystic kidney disease. Nat. Genet. *49*, 1025–1034.
20. Srivastava, S., Ramsbottom, S.A., Molinari, E., Alkanderi, S., Filby, A., White, K., Henry, C., Saunier, S., Miles, C.G., and Sayer, J.A. (2017). A human patient-derived cellular model of Joubert syndrome reveals ciliary defects which can be rescued with targeted therapies. Hum. Mol. Genet. *26*, 4657–4667.
21. Ajzenberg, H., Slaats, G.G., Stokman, M.F., Arts, H.H., Logister, I., Kroes, H.Y., Renkema, K.Y., van Haelst, M.M., Terhal, P.A., van Rooij, I.A., et al. (2015). Non-invasive sources of cells with primary cilia from pediatric and adult patients. Cilia *4*, 8.
22. Perner, B., Englert, C., and Bollig, F. (2007). The Wilms tumor genes wt1a and wt1b control different steps during formation of the zebrafish pronephros. Dev. Biol. *309*, 87–96.
23. Kimmel, C.B., Ballard, W.W., Kimmel, S.R., Ullmann, B., and Schilling, T.F. (1995). Stages of embryonic development of the zebrafish. Dev. Dyn. *203*, 253–310.
24. Szklarczyk, D., Gable, A.L., Nastou, K.C., Lyon, D., Kirsch, R., Pyysalo, S., Doncheva, N.T., Legeay, M., Fang, T., Bork, P., et al. (2021). The STRING database in 2021: customizable protein-protein networks, and functional characterization of user-uploaded gene/measurement sets. Nucleic Acids Res. *49* (D1), D605–D612.
25. Badgandi, H.B., Hwang, S.-H., Shimada, I.S., Loriot, E., and Mukhopadhyay, S. (2017). Tubby family proteins are adapters for ciliary trafficking of integral membrane proteins. J. Cell Biol. *216*, 743–760.

26. Ikeda, A., Ikeda, S., Gridley, T., Nishina, P.M., and Naggert, J.K. (2001). Neural tube defects and neuroepithelial cell death in Tulp3 knockout mice. Hum. Mol. Genet. *10*, 1325–1334.
27. Dickinson, M.E., Flenniken, A.M., Ji, X., Teboul, L., Wong, M.D., White, J.K., Meehan, T.F., Weninger, W.J., Westerberg, H., Adissu, H., et al. (2016). High-throughput discovery of novel developmental phenotypes. Nature *537*, 508–514.
28. Turro, E., Astle, W.J., Megy, K., Gräf, S., Greene, D., Shamardina, O., Allen, H.L., Sanchis-Juan, A., Frontini, M., Thys, C., et al. (2020). Whole-genome sequencing of patients with rare diseases in a national health system. Nature *583*, 96–102.
29. Sobreira, N., Schiettecatte, F., Valle, D., and Hamosh, A. (2015). GeneMatcher: a matching tool for connecting investigators with an interest in the same gene. Hum. Mutat. *36*, 928–930.
30. Bergmann, C., Senderek, J., Küpper, F., Schneider, F., Dornia, C., Windelen, E., Eggermann, T., Rudnik-Schöneborn, S., Kirfel, J., Furu, L., et al. (2004). PKHD1 mutations in autosomal recessive polycystic kidney disease (ARPKD). Hum. Mutat. *23*, 453–463.
31. White, R.J., Collins, J.E., Sealy, I.M., Wali, N., Dooley, C.M., Digby, Z., Stemple, D.L., Murphy, D.N., Billis, K., Hourlier, T., et al. (2017). A high-resolution mRNA expression time course of embryonic development in zebrafish. eLife *6*, e30860.
32. Mukhopadhyay, S., Wen, X., Ratti, N., Loktev, A., Rangell, L., Scales, S.J., and Jackson, P.K. (2013). The ciliary G-protein-coupled receptor Gpr161 negatively regulates the Sonic hedgehog pathway via cAMP signaling. Cell *152*, 210–223.
33. Bielas, S.L., Silhavy, J.L., Brancati, F., Kisseleva, M.V., Al-Gazali, L., Sztriha, L., Bayoumi, R.A., Zaki, M.S., Abdel-Aleem, A., Rosti, R.O., et al. (2009). Mutations in INPP5E, encoding inositol polyphosphate-5-phosphatase E, link phosphatidyl inositol signaling to the ciliopathies. Nat. Genet. *41*, 1032–1036.
34. Kerek, E.M., Yoon, K.H., Luo, S.Y., Chen, J., Valencia, R., Julien, O., Waskiewicz, A.J., and Hubbard, B.P. (2021). A conserved acetylation switch enables pharmacological control of tubby-like protein stability. J. Biol. Chem. *296*, 100073.
35. Yuan, Z., Zhang, X., Sengupta, N., Lane, W.S., and Seto, E. (2007). SIRT1 regulates the function of the Nijmegen breakage syndrome protein. Mol. Cell *27*, 149–162.
36. Dell'Omo, G., Crescenti, D., Vantaggiato, C., Parravicini, C., Borroni, A.P., Rizzi, N., Garofalo, M., Pinto, A., Recordati, C., Scanziani, E., et al. (2019). Inhibition of SIRT1 deacetylase and p53 activation uncouples the anti-inflammatory and chemopreventive actions of NSAIDs. Br. J. Cancer *120*, 537–546.
37. Langley, E., Pearson, M., Faretta, M., Bauer, U.-M., Frye, R.A., Minucci, S., Pelicci, P.G., and Kouzarides, T. (2002). Human SIR2 deacetylates p53 and antagonizes PML/p53-induced cellular senescence. EMBO J. *21*, 2383–2396.
38. Huang, X.-Z., Wen, D., Zhang, M., Xie, Q., Ma, L., Guan, Y., Ren, Y., Chen, J., and Hao, C.-M. (2014). Sirt1 activation ameliorates renal fibrosis by inhibiting the TGF-β/Smad3 pathway. J. Cell. Biochem. *115*, 996–1005.
39. Liu, Z.-H., Zhang, Y., Wang, X., Fan, X.-F., Zhang, Y., Li, X., Gong, Y.S., and Han, L.-P. (2019). SIRT1 activation attenuates cardiac fibrosis by endothelial-to-mesenchymal transition. Biomed. Pharmacother. *118*, 109227.
40. Samarakoon, R., Higgins, C.E., Higgins, S.P., and Higgins, P.J. (2009). TGF-beta1-Induced Expression of the Poor Prognosis SERPINE1/PAI-1 Gene Requires EGFR Signaling: A New Target for Anti-EGFR Therapy. J. Oncol. *2009*, 342391.
41. Eastman, Q., and Grosschedl, R. (1999). Regulation of LEF-1/TCF transcription factors by Wnt and other signals. Curr. Opin. Cell Biol. *11*, 233–240.
42. Park, E., Lee, J.M., Ahn, Y.H., Kang, H.G., Ha, I.I., Lee, J.H., Park, Y.S., Kim, N.K.D., Park, W.-Y., and Cheong, H.I. (2016). Hepatorenal fibrocystic diseases in children. Pediatr. Nephrol. *31*, 113–119.
43. Gerhard, G.S., Kauffman, E.J., Wang, X., Stewart, R., Moore, J.L., Kasales, C.J., Demidenko, E., and Cheng, K.C. (2002). Life spans and senescent phenotypes in two strains of Zebrafish (Danio rerio). Exp. Gerontol. *37*, 1055–1068.
44. Clement, C.A., Ajbro, K.D., Koefoed, K., Vestergaard, M.L., Veland, I.R., Henriques de Jesus, M.P.R., Pedersen, L.B., Benmerah, A., Andersen, C.Y., Larsen, L.A., and Christensen, S.T. (2013). TGF-β signaling is associated with endocytosis at the pocket region of the primary cilium. Cell Rep. *3*, 1806–1814.
45. Villalobos, E., Criollo, A., Schiattarella, G.G., Altamirano, F., French, K.M., May, H.I., Jiang, N., Nguyen, N.U.N., Romero, D., Roa, J.C., et al. (2019). Fibroblast Primary Cilia Are Required for Cardiac Fibrosis. Circulation *139*, 2342–2357.
46. Cigna, N., Farrokhi Moshai, E., Brayer, S., Marchal-Somme, J., Wémeau-Stervinou, L., Fabre, A., Mal, H., Lesèche, G., Dehoux, M., Soler, P., et al. (2012). The hedgehog system machinery controls transforming growth factor-β-dependent myofibroblastic differentiation in humans: involvement in idiopathic pulmonary fibrosis. Am. J. Pathol. *181*, 2126–2137.
47. Cao, H., Chen, X., Hou, J., Wang, C., Xiang, Z., Shen, Y., and Han, X. (2020). The Shh/Gli signaling cascade regulates myofibroblastic activation of lung-resident mesenchymal stem cells via the modulation of Wnt10a expression during pulmonary fibrogenesis. Lab. Invest. *100*, 363–377.

The American Journal of Human Genetics, Volume *109*

Supplemental information

Progressive liver, kidney, and heart degeneration in children and adults affected by *TULP3* mutations

John Devane, Elisabeth Ott, Eric G. Olinger, Daniel Epting, Eva Decker, Anja Friedrich, Nadine Bachmann, Gina Renschler, Tobias Eisenberger, Andrea Briem-Richter, Enke Freya Grabhorn, Laura Powell, Ian J. Wilson, Sarah J. Rice, Colin G. Miles, Katrina Wood, Genomics England Research Consortium, Palak Trivedi, Gideon Hirschfield, Andrea Pietrobattista, Elizabeth Wohler, Anya Mezina, Nara Sobreira, Emanuele Agolini, Giuseppe Maggiore, Mareike Dahmer-Heath, Ali Yilmaz, Melanie Boerries, Patrick Metzger, Christoph Schell, Inga Grünewald, Martin Konrad, Jens König, Bernhard Schlevogt, John A. Sayer, and Carsten Bergmann

Supplemental Figures

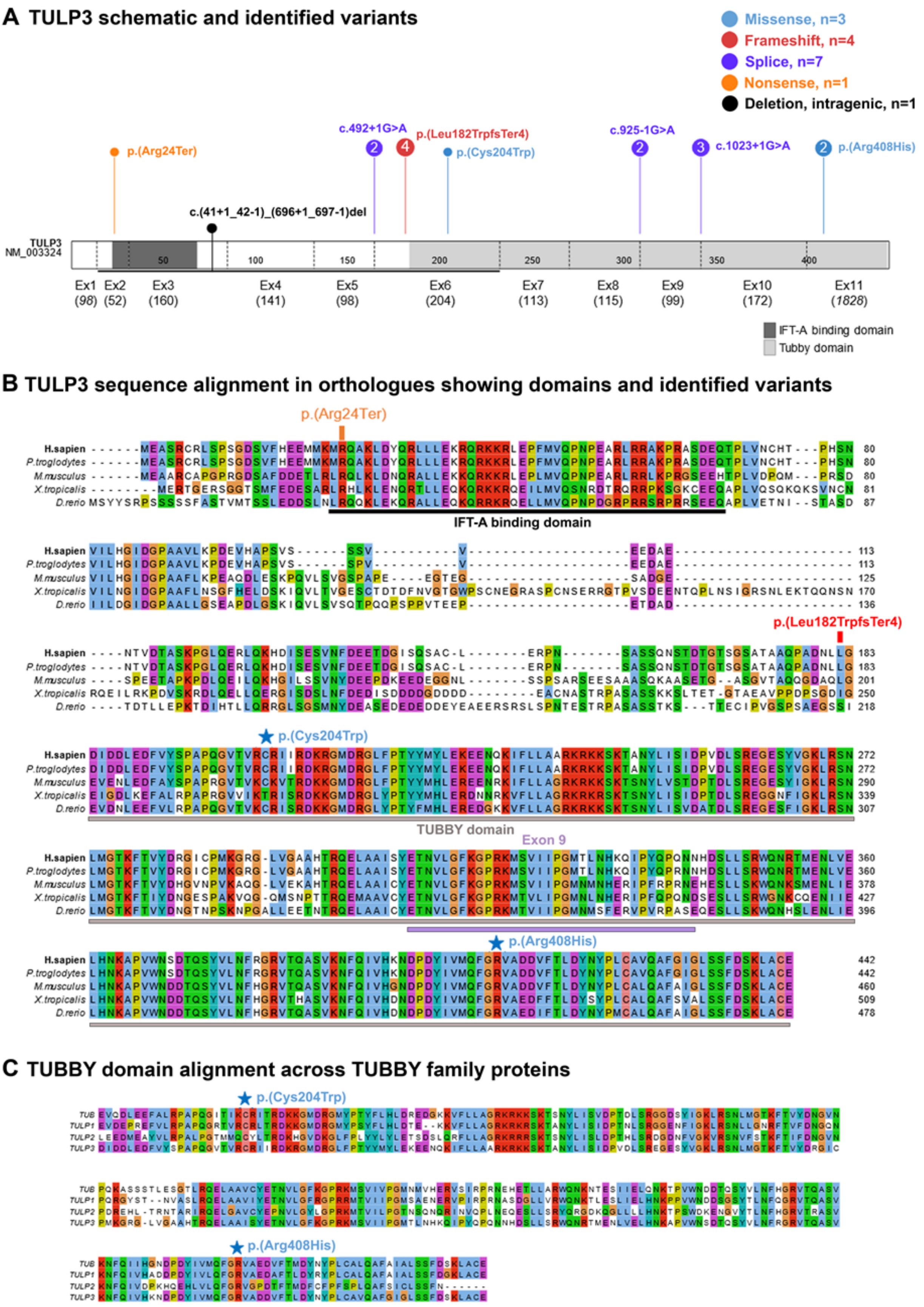

Figure S1. Overview of identified *TULP3* variants, conservation across species and functional domains.

Panel A is a schematic of TULP3, exons are shown bounded by dotted lines and nucleotide length (including untranslated regions) is indicated. The IFT-A binding domain and the tubby domain are shown in black or grey shades, as indicated. Each of the eight variants we identified is mapped into its respective amino acid position or exon boundary for intronic variants. The variant type is annotated, and color coded. Disc number reflects the mutant allele count in family probands. **Panel B** represents amino acid sequence alignments for canonical *TULP3* transcripts for the indicated species. Functional domains as well as exon 9 (targeted by 2 distinct predicted splice-affecting variants) are depicted with colored lines. The nonsense, missense and frameshifting variants are indicated above the alignments. **Panel C** shows tubby domain amino acid alignments for different members of the Tubby protein family and highlights the identified missense variants. Sequences correspond to human canonical transcripts. Sequences were retrieved via Ensembl and alignments performed with Clustal omega.

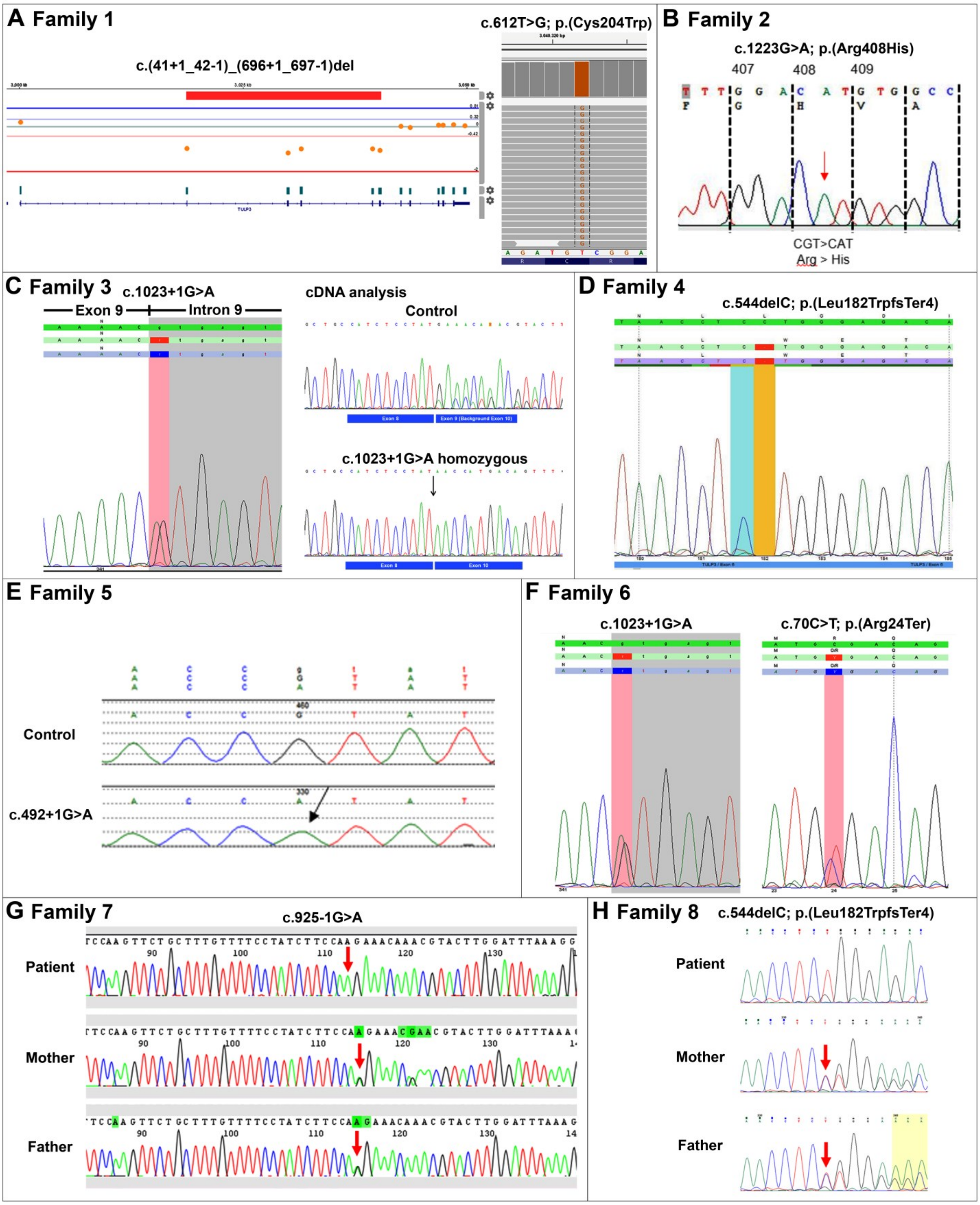

Figure S2. NGS analysis and/or chromatograms for the probands of each family.

Panel A shows the read count obtained from NGS sequencing showing heterozygous c.(41+1_42-1)_(696+1_697-1) deletion in *TULP3* in which exons 2-6 are deleted, and an Integrative Genomics View (IGV) of BAM file read alignments for mutation c.612T>G;

p.(Cys204Trp). **Panel B** shows a Sanger chromatogram of the homozygous c.1223G>A; p.(Arg408His) change observed in Family 2. **Panel C** shows electropherograms of healthy parental and affected proband samples of Family 3. Affected individuals harbor a homozygous c.1023+1G>A change that results in an in-frame skipping of exon 9 and parental samples are heterozygous for this allele. **Panel D** shows an electropherogram of the homozygous mutation c.544delC*;* p.(Leu182TrpfsTer4) detected in Family 4. **Panel E** shows a Sanger chromatogram of the homozygous mutation c.492+1G>A observed in family 5. The black arrow denotes the position of the change. **Panel F** shows two electropherograms from an affected individual from Family 6 displaying the compound heterozygous mutations c.1023+1G>A and c.70C>T; p.(Arg24Ter). **Panel G** shows a Sanger chromatogram of the homozygous mutation c.925-1G>A observed in Family 7. **Panel H** shows a Sanger chromatogram from an affected individual from Family 8 with the homozygous mutation c.544delC; p.(Leu182TrpfsTer4) and his heterozygous parents.

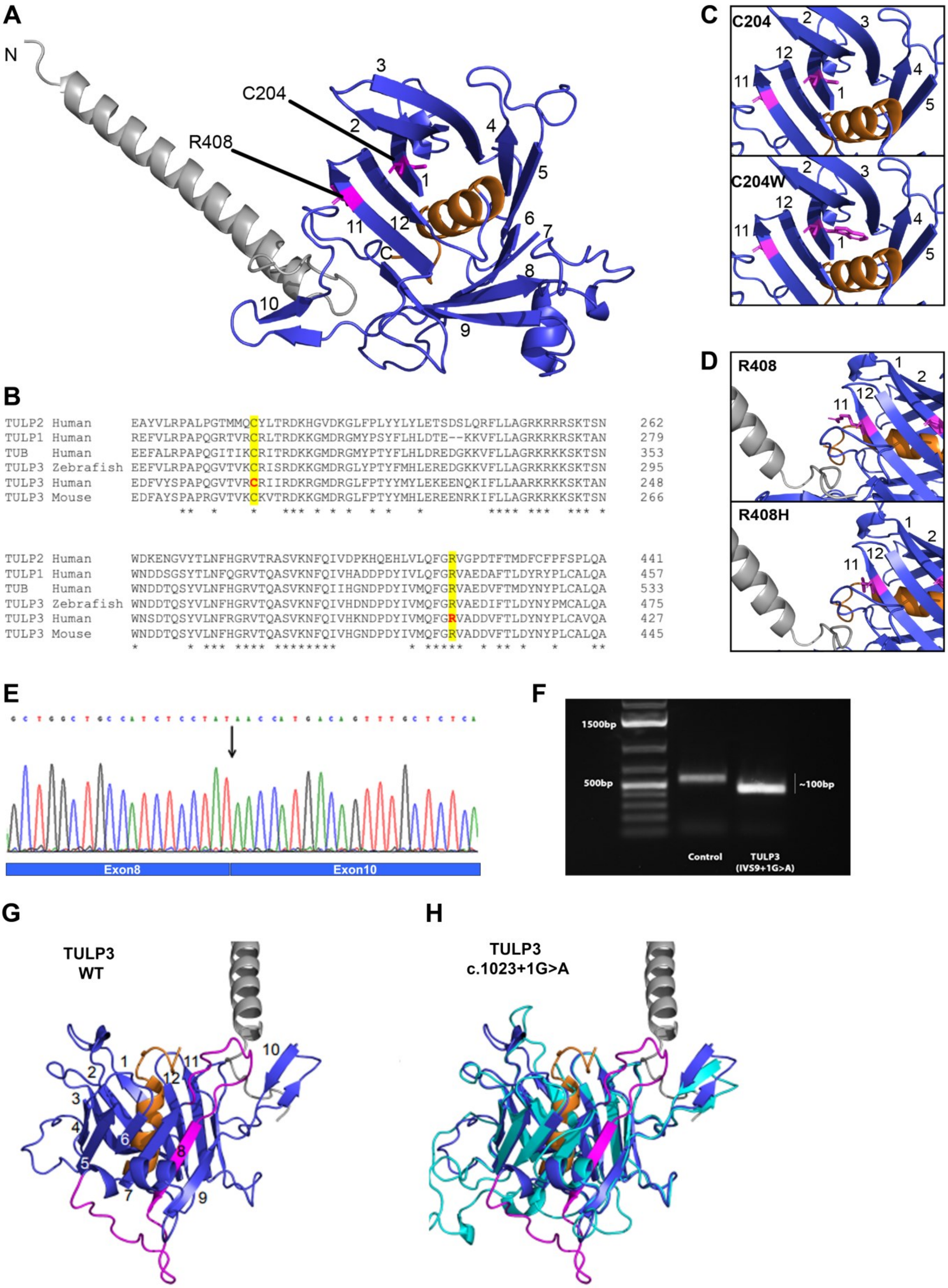
A
N
C204
R408
1
2
3
4
5
6
7
8
9
10
11
12
C
B
TULP2 Human EAYVLRPALPGTMMQCYLTRDKHGVDKGLFPLYYLYLETSDSLQRFLLAGRKRRRSKTSN 262
TULP1 Human REFVLRPAPQGRTVRCRLTRDKKGMDRGMYPSYFLHLDTE--KKVFLLAGRKRKRSKTAN 279
TUB Human EEFALRPAPQGITIKCRITRDKKGMDRGMYPTYFLHLDREDGKKVFLLAGRKRKKSKTSN 353
TULP3 Zebrafish EEFVLRPAPQGVTVKCRISRDKKGMDRGLYPTYFMHLEREDGKKVFLLAGRKRKKSKTSN 295
TULP3 Human EDFVYSPAPQGVTVRCRIIRDKRGMDRGLFPTYYMYLEKEENQKIFLLAARKRKKSKTAN 248
TULP3 Mouse EDFAYSPAPRGVTVKCKVTRDKKGMDRGLFPTYYMHLEREENRKIFLLAGRKRKKSKTSN 266
TULP2 Human WDKENGVYTLNFHGRVTRASVKNFQIVDPKHQEHLVLQFGRVGPDTFTMDFCFPFSPLQA 441
TULP1 Human WNDDSGSYTLNFQGRVTQASVKNFQIVHADDPDYIVLQFGRVAEDAFTLDYRYPLCALQA 457
TUB Human WNDDTQSYVLNFHGRVTQASVKNFQIIHGNDPDYIVMQFGRVAEDVFTMDYNYPLCALQA 533
TULP3 Zebrafish WNDDTQSYVLNFHGRVTQASVKNFQIVHDNDPDYIVMQFGRVAEDIFTLDYNYPMCALQA 475
TULP3 Human WNSDTQSYVLNFRGRVTQASVKNFQIVHKNDPDYIVMQFGRVADDVFTLDYNYPLCAVQA 427
TULP3 Mouse WNDDTQSYVLNFHGRVTQASVKNFQIVHGNDPDYIVMQFGRVADDVFTLDYNYPLCALQA 445
C
C204
C204W
D
R408
R408H
E
Exon8
Exon10
F
1500bp
500bp
~100bp
Control
TULP3
(IVS9+1G>A)
G
TULP3
WT
H
TULP3
c.1023+1G>A

Figure S3. ***In silico*** **modeling of** ***TULP3*** **variants.**

Panel A Predicted model of human TULP3 displaying the regions modelled by AlphaFold with very high confidence (per-residue confidence score (pLDDT) > 90). The N-terminal alpha helix is coloured in grey. The twelve β strands of the tubby domain β barrel are numbered and coloured in blue. The α helix, central to the hydrophobic core of the tubby domain is coloured in orange. **Panel B** Multiple sequence alignment of human TULP proteins (TUB and TULP1-3) and mouse and zebrafish TULP3. The positions of missense mutations C204W and R408H are highlighted in yellow. *, completely conserved residue in all aligned sequences. **Panel C** Upper panel: A rotated and zoomed in view of the C204 residue (magenta) within β sheet 1 of the tubby domain. Lower panel, *in silico* mutagenesis of TULP3 C204 to W204, resulting in the presence of a large hydrophobic tryptophan residue. **Panel D** Upper panel: A rotated and zoomed in view of the R408 residue (magenta) within β sheet 11 of the tubby domain. Lower panel, *in silico* mutagenesis of TULP3 R408 to H408. **Panel E** Results of RT-PCR Sanger sequencing of *TULP3* c.1023+1G>A fibroblasts derived from affected individual aligned against the exons of *TULP3*, showing in frame skipping of *TULP3* exon 9. **Panel F** shows gel electrophoresis comparing the size of cDNA fragments of *TULP3* generated from RNA isolated from control fibroblasts and *TULP3* c.1023+1G>A fibroblasts (RT-PCR). Fragments generated using primers targeted against the 3' half of *TULP3* (including exon 9) resulting in an expected fragment size of 600 bp. We observe about 100 bp reduction in the size of fragments generated from *TULP3* c.1023+1G>A samples. This is consistent with the predicted in-frame skipping of exon 9 in these affected individuals (99 bp). Left is a DNA ladder with 500 bp and 1500 bp annotated (GeneRuler 1kb plus). **Panel G** Predicted model of human TULP3 displaying the regions modelled by AlphaFold with very high confidence (per-residue confidence score (pLDDT) >90). The N-terminal alpha helix is coloured in grey. The twelve β strands of the tubby domain β barrel are numbered and coloured in dark blue. The α helix, central to the hydrophobic core of the tubby domain is coloured in orange. **Panel H** Predicted model of human TULP3 c.1023+1G>A tubby domain barrel created using HHPred based on structural homology to human TUB1 (PDB, 3C5N), coloured in cyan and superposed on the

wild-type structure as shown in panel G (in blue, orange and pink). Strand 8 of the tubby domain β barrel (coloured in pink on the wild-type structure) is missing from the mutant structure.

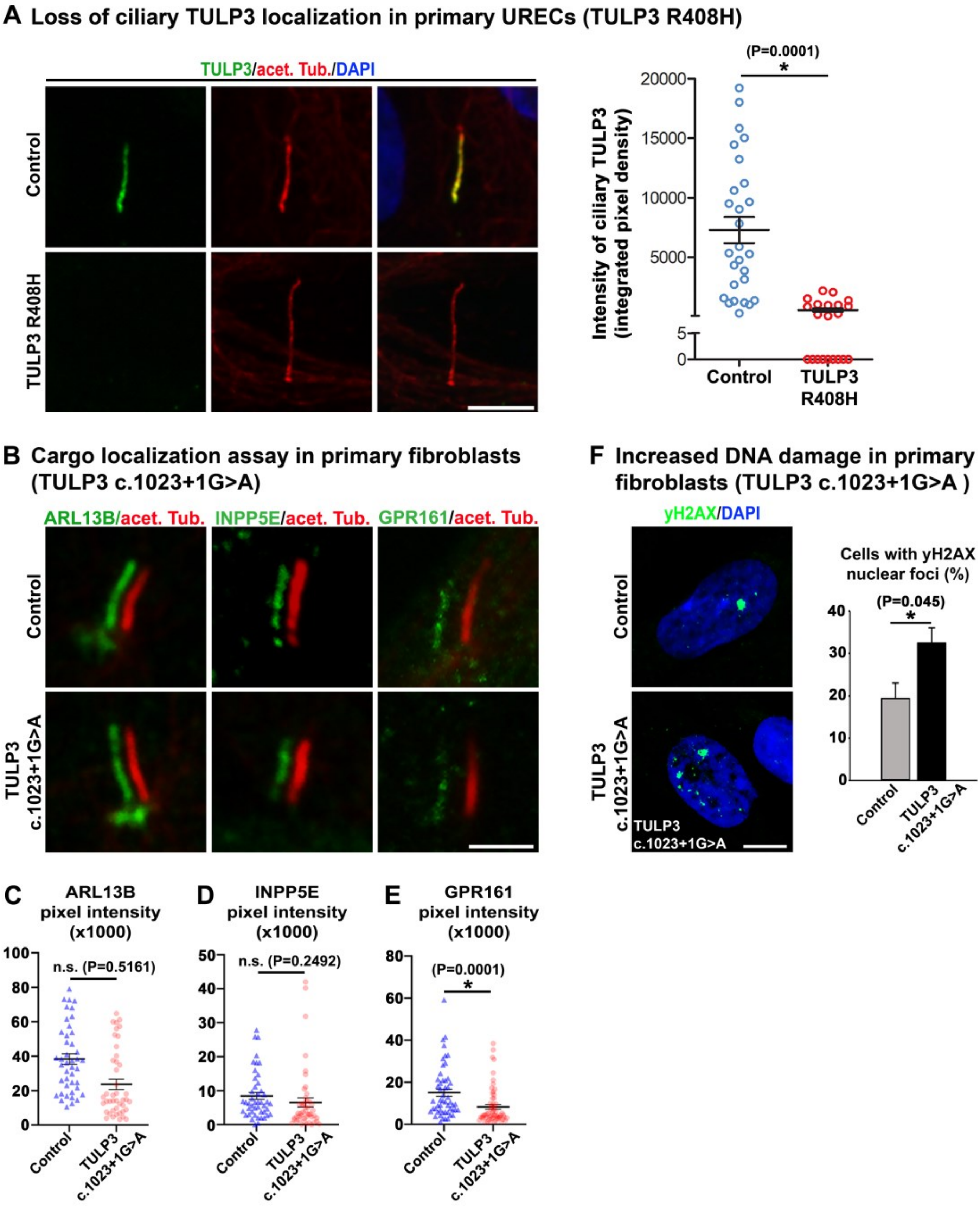
A Loss of ciliary TULP3 localization in primary URECs (TULP3 R408H)
TULP3/acet. Tub./DAPI
Control
TULP3 R408H
(P=0.0001)
*
Intensity of ciliary TULP3 (integrated pixel density)
20000
15000
10000
5000
5
0
Control
TULP3 R408H
B Cargo localization assay in primary fibroblasts (TULP3 c.1023+1G>A)
ARL13B/acet. Tub.
INPP5E/acet. Tub.
GPR161/acet. Tub.
Control
TULP3 c.1023+1G>A
F Increased DNA damage in primary fibroblasts (TULP3 c.1023+1G>A)
γH2AX/DAPI
Control
TULP3 c.1023+1G>A
TULP3 c.1023+1G>A
Cells with γH2AX nuclear foci (%)
(P=0.045)
*
40
30
20
10
0
Control
TULP3 c.1023+1G>A
C ARL13B pixel intensity (x1000)
n.s. (P=0.5161)
100
80
60
40
20
0
Control
TULP3 c.1023+1G>A
D INPP5E pixel intensity (x1000)
n.s. (P=0.2492)
50
40
30
20
10
0
Control
TULP3 c.1023+1G>A
E GPR161 pixel intensity (x1000)
(P=0.0001)
*
80
60
40
20
0
Control
TULP3 c.1023+1G>A

Figure S4. Ciliary trafficking in additional cells derived from affected individuals.

Panel A Left: Confocal microscope images showing ciliary localisation of TULP3 in control URECs. In contrast, ciliary expression of TULP3 is lost in URECs of affected individual Family 2 (II.1) (p.(Arg408His)). Right: Relative intensity of ciliary TULP3 signal in control URECs and URECs derived from the affected individual represented as integrated pixel intensity. **Panel B** shows localisation of TULP3 cargos in cilia from control fibroblasts and fibroblasts from Family 3 proband (II.2) (c.1023+1G>A). Color channels are offset to better visualize the signals. **Panel C-E** shows quantification of TULP3 cargo (ARL13B, INPP5E and GPR161) intensity in control fibroblasts and fibroblasts from the affected individual represented as pixel intenisities. **Panels F** shows increased γH2AX nuclear foci in fibroblasts from Family 3 proband (II.2) compared to age-matched controls. For panels A-F: * $P<0.05$ (two-tailed, unpaired student's t-test); error bars represent SEM; scale bars, 5 μm.

A TULP3 physical interaction network (TAP)

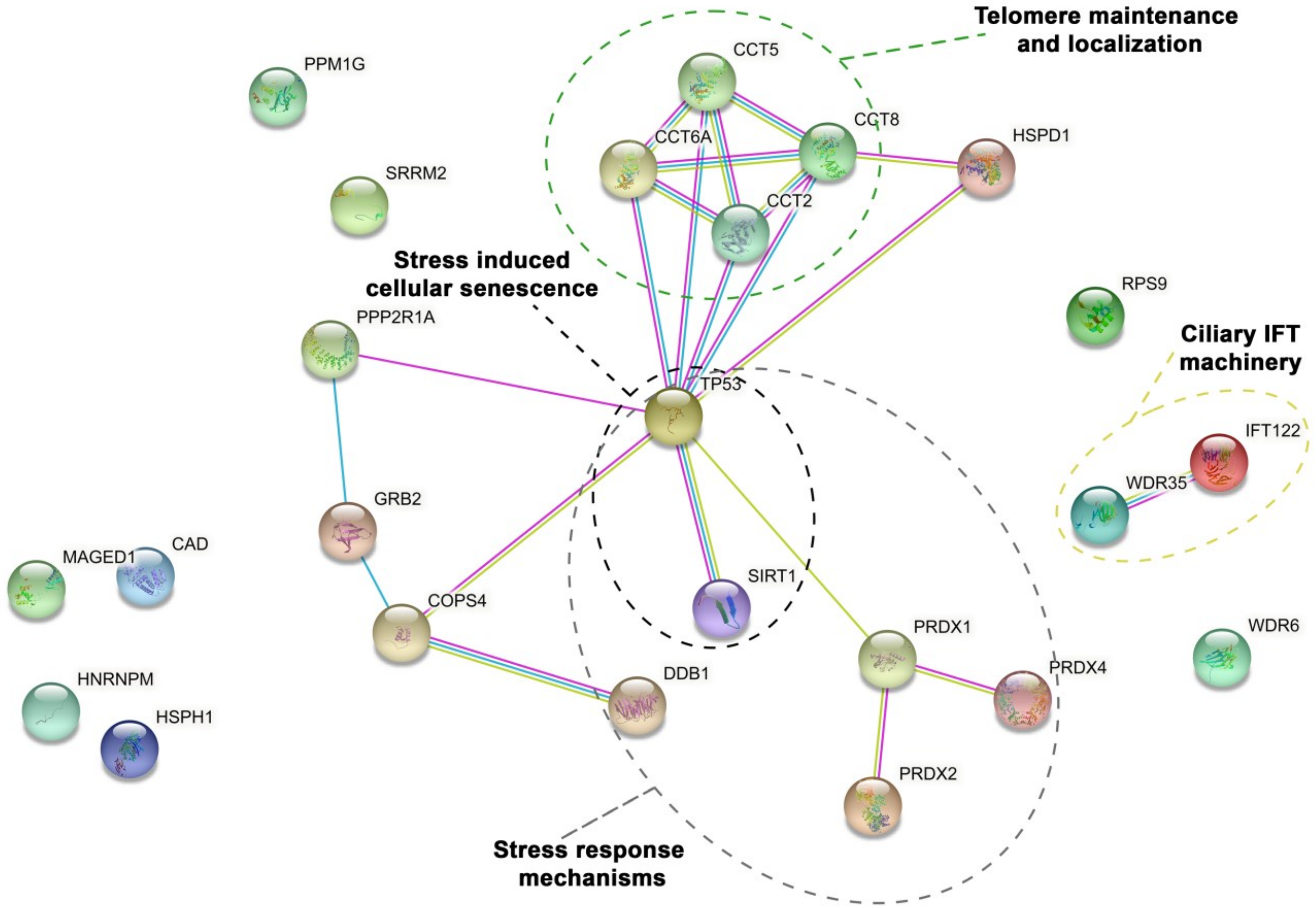

B Functional enrichment (GO: Biological processes)

#term ID	Term description	Observed gene count	Background gene count	Strength	False discovery rate
GO:0051097	negative regulation of helicase activity	2	5	2.51	0.0135
GO:1904851	positive regulation of establishment of protein localization to telomere	4	10	2.51	3.01E-05
GO:1904871	positive regulation of protein localization to Cajal body	4	11	2.47	3.01E-05
GO:1904874	positive regulation of telomerase RNA localization to Cajal body	4	15	2.34	3.01E-05
GO:0090400	stress-induced premature senescence	2	8	2.31	0.023
GO:0035721	intraciliary retrograde transport	2	10	2.21	0.0305
GO:0019430	removal of superoxide radicals	2	13	2.1	0.0385
GO:0045722	positive regulation of gluconeogenesis	2	14	2.07	0.0404
GO:0070914	UV-damage excision repair	2	14	2.07	0.0404
GO:0090343	positive regulation of cell aging	2	15	2.04	0.0424

Figure S5. TULP3 physical interaction network: DNA damage response and ciliary transport.

Panel A shows a STRING database interaction network generated with the TULP3 interaction partners identified through affinity purification mass spectroscopy using N-terminally FLAG-tagged TULP3 as bait in HEK293T cells. Physical interaction network visualised using the

STRING online tool with default settings (V11.5). Medium confidence (0.400). Dotted circles denote proteins with shared functions (summarized from functional enrichment in Panel B). **Panel B** shows functional enrichment analysis of the identified TULP3 interaction partners. The table shows GO term - biological processes that are overrepresented in the list of TULP3 interaction partners. Strength refers to background gene count/observed gene count. Shown are the top 10 hits ordered by strength with corrected P-value <0.05. False discovery rate (FDR) determined using Benjamini-Hochberg procedure.

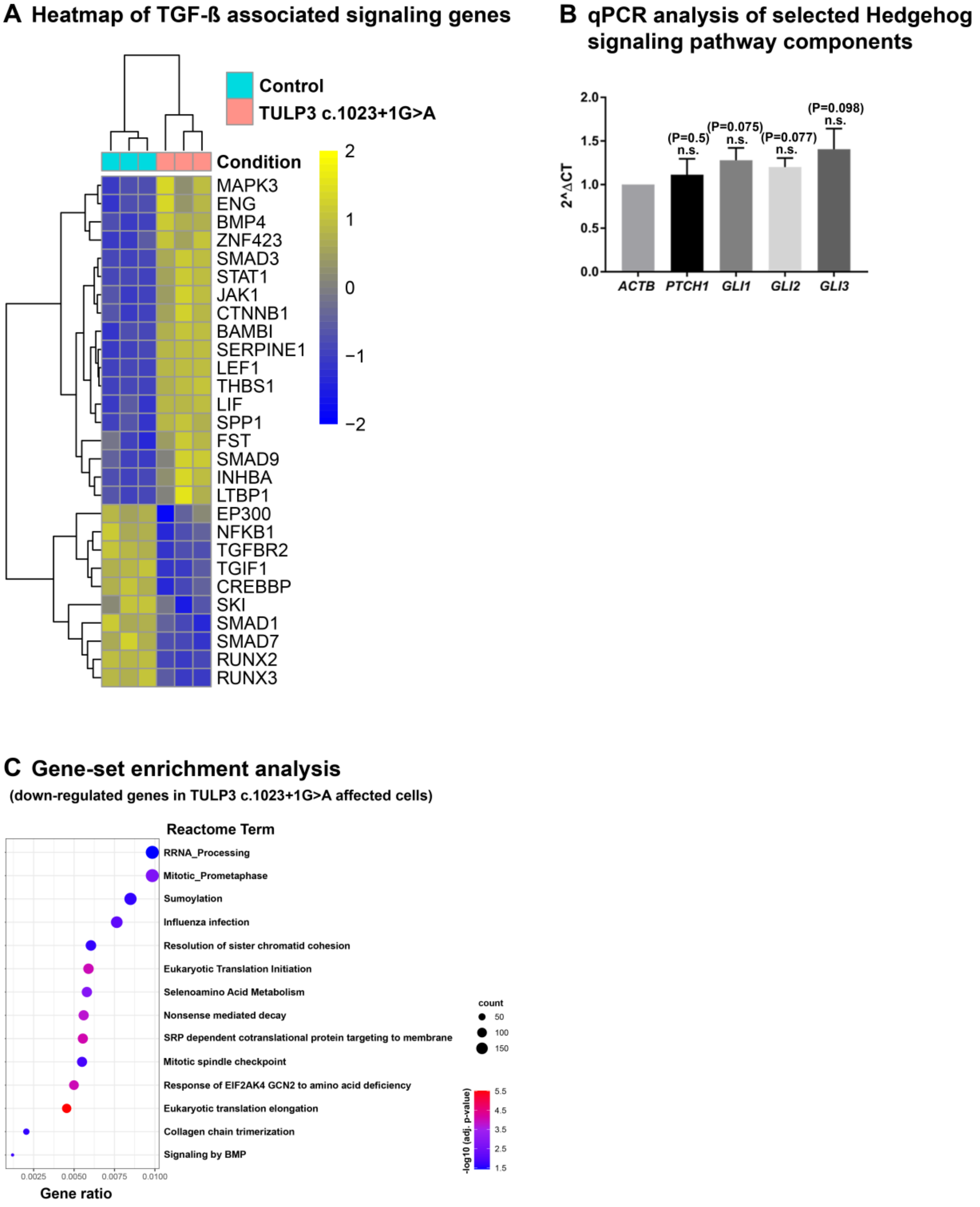

Figure S6. TGF-β pathway associated genes are significantly up-regulated in cells derived from *TULP3* proband.

Panel A shows a gene expression heatmap for differentially regulated genes from the GSEA term “TGF-β signaling pathway”. Dysregulated genes identified through RNA sequencing of fibroblast cells derived from Family 3 proband (II.2) are shown. Each column represents an individual sample from unaffected control or affected proband-derived cells. Yellow to blue

color-codes indicating up- or down-regulation are presented on the right; differentially expressed genes with P-values $P<0.05$ are shown. **Panel B** shows qPCR analysis of selected SHH pathway components in fibroblasts derived from Family 3 proband (II.2). *ACTB* used as housekeeping gene. n.s.= $P>0.05$; error bars represent SEM (two-tailed, unpaired student's t-test). **Panel C** shows reactome terms of significantly down-regulated genes in fibroblast cells derived from Family 3 proband (II.2). Differentially regulated genes were identified by the Gene set analysis method. Pathways were considered significant with adjusted p-values (Benjamini-Hochberg) $P<0.05$. Significantly down-regulated genes associated predominantly with cell cycle, ribosome and circadian rhythm related signaling processes.

Supplemental Methods

Massively parallel sequencing

We utilized a customized sequence capture library (e.g. by Twist Bioscience©) with curated target regions - currently comprising more than 600 genes described and associated with kidney disease or allied disorders - as well as corresponding flanking intronic sequences according to the manufacturer´s recommendations for probands of Families 1, 3, 4 and 6. The panel design is constantly updated by surveillance of current literature as well as enriched by targets in non-coding regions for described variants listed in well-accepted databases like HGMD or ClinVar. Moreover, the design is optimized in low-performance regions as well as in critical regions like in *PKD1* as described [1,2]. DNA samples were pooled and sequenced in a multiplexing procedure. DNAs were enriched using a sequence capture approach and sequenced using Illumina sequencing-by-synthesis technology with an average coverage of more than 300X for a targeted panel setup. Raw data were processed according to bioinformatics best practice procedures. Mapping and coverage statistics were generated from the mapping output files using standard bioinformatics tools (e.g. Picard). High and reproducible coverage achieved by our sequencing approach enabled copy number variation (CNV) analysis. Performance of the wet-lab and bioinformatic processes are validated and controlled according to national and international guidelines [3,4] reaching high sensitivity for single nucleotide variants (SNVs), indels and CNVs using well-established reference samples as well as a large cohort of positive controls, especially for CNVs. For interpretation of identified variants, we have developed own published bioinformatic algorithms using a stepwise filtering process conducted by an experienced team of scientists and supported by various bioinformatics decision tools. Sequence variants of interest were verified by Sanger sequencing if next generation sequencing (NGS) results failed internal validation guidelines. If other family members were available, segregation of sequence variants with the disease was further assessed.

Families 2 and 5 were identified by whole genome sequencing (WGS) performed by Genomics England (GE). The 100,000 Genomes Project provides a rich source of WGS data on individuals with rare disease phenotypes. As of September 2019, WGS was performed in 35,042 probands affected by rare diseases, including 3,934 probands with various renal and urinary tract disorders. WGS was performed by GE via the 100,000 Genomes Project using the Illumina TruSeq DNA PCR-Free sample preparation kit (Illumina, Inc.) and an Illumina HiSeq 2500 sequencer, generating a mean depth of 45x (range from 34x to 72x) and greater than 15x for at least 95% of the reference human genome. WGS reads were aligned to the Genome Reference Consortium human genome build 37 (GRCh37/hg19) using Isaac Genome Alignment Software (version 01.14; Illumina, Inc.). Sequence data was analysed using bcftools scripts designed to search vcf.gz files.

GeneMatcher was used to identify additional cases in Families 7 and 8. GeneMatcher is a database developed as part of the MatchMaker Exchange and has been shown to facilitate rare disease gene discovery [5]. Genomic analysis for Family 7 was performed at Johns Hopkins as part of the Baylor-Hopkins Center for Mendelian Genomics project. For these affected individuals, libraries from genomic DNA were constructed using the Agilent SureSelect HumanAllExonV5Clinical_S06588914 kit to capture the total ~52 Mb CCDS exonic regions as well as flanking intronic regions. Whole exome sequencing (WES) was performed using the Illumina HiSeq 2500 sequencer. WES reads were aligned to the 1000 Genomes phase 2 (GRCh37) human genome reference build with the Burrows-Wheeler Alignment tool version 0.7.8 [6] and Genome Analysis Toolkit (GATK) version v3.1-1-g07a4bf8 or v3.3-0-g37228af [7]. Variants were subsequently filtered using the Variant Quality Score Recalibration method [8,9]. Annotation of SNVs was performed using the MQRankSum, HaplotypeScore, QD, FS, MQ, ReadPosRankSum adaptive error model.

Imaging data and histology of liver, kidney and heart tissues of affected individuals

Tissue samples were taken for histopathological diagnosis and processed in the institutes of pathology according to the standard protocols for routine diagnostics.

Immunofluorescence analysis of cells derived from affected individuals

For cilia imaging, human urine-derived renal epithelial cells (URECs) were seeded on coverslips and grown to 90% confluence then starved for 48 h (in FBS-free media). Cells were fixed in ice-cold methanol for 10 min. After 30 min saturation with 5% BSA in PBS, cells were incubated for 1 h at room temperature (RT) with primary antibodies. Fibroblast cells were seeded on ibidi slides, grown to 90% confluence and starved for 72 h (in media with 0.1% FBS). These cells were fixed in 4% PFA for 5 min. After 1 h saturation with 5% BSA in PBS, fibroblasts were incubated over night at 4°C with primary antibodies. The following primary antibodies were used: mouse anti-acetylated α-Tubulin, rabbit anti-ARL13B, mouse anti-ARL13B, rabbit anti-INPP5E, rabbit anti-GPR161, rabbit anti-TULP3, rabbit anti-γH2AX (listed below) followed by washing steps in PBS. Cells were incubated at RT for 1 h with the following secondary antibodies: donkey anti-rabbit Alexa Fluor 488; donkey anti-mouse Alexa Fluor 594. Nuclei were counterstained with 4′,6-diamidino-2-phenylindole (DAPI) using NucBlueTM (Thermo Fischer Scientific, R37606). Images and z-stacks of URECs were captured using a Nikon (A1) confocal inverted microscope. Images and z-stacks of fibroblasts were captured using a Zeiss LSM 880 Observer confocal inverted microscope. Identical laser intensity as well as camera settings were used for control and cells derived from affected individuals (unless specified otherwise). Following capture, images were analysed using FIJI (ImageJ) software. Fluorescence intensity of individual cilia or nuclei were measured on a sum of slices projection of a z-stack. A region of interest (ROI) was constructed around the cilia (identified with ARL13B staining or acetylated α-Tubulin staining) or the nucleus (identified with DAPI) to measure the integrated density. To correct for local background intensity, the ROI was duplicated and dragged to a nearby region and background integrated density was measured and subtracted. To detect the fraction of GPR161-positive cilia, ROI around cilia were constructed using ARL13B staining. ROI were then transposed on the GPR161 z-stack channel that was visually screened for GPR161 signal in control cells and cells derived from affected individuals under identical laser intensity and camera settings.

Target protein	Use	Dilution	Company	Comment	Species
acetylated α-Tubulin	IF	1:2000, 1:10000	Sigma, T6793		Mouse
ARL13B	IF	1:300	Proteintech,17711-1-AP		Rabbit
ARL13B	IF	1:300	Proteintech, 66739-1-IG		Mouse
INPP5E	IF	1:100-1:200	Proteintech,17797-1-AP		Rabbit
TULP3	IF	1:300-1:500	Abcam, ab155317	detects aa129-403	Rabbit
GPR161	IF	1:100	obtained from S. Mukhopadhyay		Rabbit
γH2AX	IF	1:100	Cell Signaling, mAb, #9718		Rabbit
Alexa Fluor 488	IF	1:300	Invitrogen, A21206		Rabbit
Alexa Fluor 488	IF	1:1000	Invitrogen, A11034		Rabbit
Alexa Fluor 594	IF	1:300	Invitrogen, A21203		Mouse
CY-3	IF	1:1000	Jackson Laboratory, 715-165-150		Mouse

Reverse-transcription polymerase chain reaction (RT-PCR) analysis for zebrafish

Semi-quantitative RT-PCR was performed to determine expression of zebrafish *tulp3* during embryonic development and in adult organs. Total RNA from entire zebrafish embryos or adult zebrafish organs was extracted with the RNeasy Kit (Qiagen), followed by complementary DNA (cDNA) synthesis with the ProtoScript First Strand cDNA Synthesis Kit (Promega). Analysis of zebrafish *ef1α* was used as a loading control. The following primers were used for temporal and spatial PCR analysis: tulp3 (forward: 5'-AGAACCTCATCGAGCTGCAT-3'; reverse: 5'-ATGTGTGTGTGTCTCAGGGT-3'), ef1α (forward: 5'-ATCTACAAATGCGGTGGAAT-3'; reverse: 5'-ATACCAGCCTCAAACTCACC-3'). The following primers were used to determine *tulp3* expression in MZ*tulp3* and respective control: tulp3 (forward: 5'-TCTGCTGGAGCAGAAGCAG-3'; reverse: 5'-GTGGATTTAGTGCTGGATGCAGAC-3').

Generation of CRISPR/Cas9-induced *tulp3* mutant zebrafish

As described previously [9,10], double-stranded tulp3 gRNAs (*tulp3*_gRNA-f 5`-TAGGGGGTTCAGAAGCTCCTGATC-3`, tulp3_gRNA-r 5`-

AAACGATCAGGAGCTTCTGAACCC-3`) were cloned into BsmBI-linearized pT7-gRNA vector (Addgene). BamHI-linearized pT7-*tulp3*-gRNA was used to transcribe *tulp3-gRNA* mRNA using the MEGAshortscript T7 kit (Thermo Fisher Scientific). XbaI-linearized pT3TS-nCas9n vector (Addgene) was used to transcribe *Cas9* mRNA using the T3 mMessage mMachine™ kit (Thermo Fisher Scientific). 1 nL containing a mixture of 50 pg *tulp3-gRNA* mRNA and 300 pg *Cas9* mRNA was injected into *Tg(wt1b:GFP)* zebrafish embryos at the 1-cell stage. For validation of gRNA efficiency genomic DNA was amplified using the following primers: tulp3e5-f 5`-TTACTGCTGACTGGCTGCAT-3` and tulp3e5-r 5`-CTGCCAGACAGACCTGAGAA-3` followed by analysis of Sanger-sequenced products. Analysis of genomic DNA of adult zebrafish identified potential founders which were outcrossed several times to *Tg(wt1b:EGFP)* zebrafish.

Microtome sectioning and histological analysis of adult zebrafish tissue

Adult zebrafish were sacrificed by immersion in tricaine (MS-222) containing Danieau's, fixed in 4% PFA for seven days, changing the fixative solution at least once. Specimens were stored in 70% EtOH until use. Whole organs were removed by dissection under a dissection microscope. Prior to embedding, samples were dehydrated in graded EtOH baths (25%, 50%, 75%, 100%), followed by graded clearance in Histoclear (Thermo Fisher Scientific) (25%, 50%, 75%, 100%). Samples were then incubated at 60°C and gradually transferred from Histoclear into paraffin wax (25%, 50%, 75%, 100%). Samples were then embedded in paraffin blocks using HistoCore Arcadia embedding module (Leica) and sectioned using a Leica RM2255 rotary microtome at 3 or 5 µm thickness and mounted on frosted microscope slides. For tissue sections of zebrafish liver, kidney and heart, H&E and PAS stains were performed under standardized conditions at the Institute of Surgical Pathology, University Medical Center Freiburg.

Cystic kidney index

Adult zebrafish kidneys were isolated, sectioned and stained with H&E as above, in the coronal plane. Light-Field microscope images of the sections were then analysed to determine

total area of kidney tissue and total luminal area. Cystic index score was obtained by calculating the amount of luminal area as a percentage of the total area of the kidney. Area measurements were performed blinded using ImageJ.

Mass spectroscopy

SILAC labelled human embryonic kidney 293T (HEK293T) cells were transiently transfected with pcDNA6-FLAG-TULP3 or pcDNA6-FLAG-GFP and lysed in IP buffer supplemented with PMSF and Na_3VO_4 (Sigma). Cell lysates were incubated with anti-FLAG M2 agarose beads for 2 h at 4°C. Beads were washed in IP buffer and incubated with Laemmli buffer for 5 min at 95°C. Samples were then treated with iodoacetamide (IAA) and excess IAA was quenched by addition of DTT. Mass spectroscopy was performed and analysed at the core facility proteomics at the Center of Biological Systems Analysis in Freiburg. Identified proteins were subjected to the STRING (https://string-db.org/ V11.5) protein association network database and a physical interaction network was generated using default settings [11].

Co-immunoprecipitation and western blotting

Calcium phosphate transfection was used to transiently express 2.5 µg of pcDNA6-FLAG-TULP3 and 2.5 µg of pcDNA6-SIRT1-V5 plasmid DNA in HEK293T cells. Cells were washed with ice-cold PBS and lysed in lysis buffer (1% Triton X-100; 20 mM Tris, pH 7.5; 50 mM NaF; 15 mM $Na_4P_2O_7$; 0.1 mM EDTA; supplemented with Na_3VO_4 and protease inhibitor mix (Roche)). Lysates were incubated with anti-FLAG M2 agarose beads or anti-V5 agarose beads for 2 h at 4°C. Western blots of lysates and immunoprecipitations were blocked in 5% BSA, incubated with anti-FLAG (Sigma) and anti-V5 (Sigma) and respective HRP conjugated antibodies. For protein detection the WesternBright ECL HRP substrate (Advansta) was used and immunoblot signals were quantified using Gel-Pro Analyzer 6.0, INTAS.

RNA sequencing analysis

RNA was isolated from affected proband-derived (Family 3 (II.2)) and control fibroblasts following 72 h incubation in DMEM (0.1% FBS) with three biological repeats for each condition.

Generation of the template library using the Illumina TruSeq stranded protocol, sequencing reactions on a HiSeq system (Illumina), initial bioinformatics processing and quality control were carried out by FASTERIS SA NGS services, Switzerland. Relative expression of genes was determined by comparing fragments per kilobase of transcript per million mapped reads (FPKM) between affected proband-derived (Family 3 (II.2)) and control samples. The raw RNA sequencing files were pre-processed with trimmomatic to ensure sufficient read quality by removing adapters and reads in low-quality segment regions with a base quality below 20. Subsequently, the reads were 2-pass aligned using the STAR aligner [14] and the GRCh37 reference genome from Ensembl. Alignment was followed by normalization and differential expression analysis with the R/Bioconductor [12,13] package DESeq2 [15]. Genes were considered significant with an adjusted P-value<0.05 (FDR corrected, according to Benjamini-Hochberg).

Gene set enrichment analysis

Enrichment analysis of signaling pathways was performed as implemented in the signaling pathways from Consensus Path DB [16]. Pathways were considered significant with an adjusted P-value<0.05 (Benjamini-Hochberg).

Quantitative PCR analysis of fibroblast cells and zebrafish embryos

For analysis of HH signaling in fibroblasts of affected individual II.2 of Family 3, total RNA from age and sex-matched healthy control and affected fibroblast cells was isolated with the RNeasy Kit (Qiagen), followed by cDNA synthesis with the Superscript II First strand cDNA Synthesis Kit (Thermo Scientific). The following primers were used for quantitative PCR (qPCR) analysis: ACTB (forward: 5'-CCAACCGCGAGAAGATGA-3'; reverse: 5'-CCAGAGGCGTACAGGGATAG-3'), GLI1 (forward: 5'-GGGATGATCCCACATCCTCAGTC -3'; reverse: 5'-CTGGAGCAGCCCCCCCAG-3'), GLI2 (forward: 5'-TGGCCGCCTCAGATGACAGATGTTG-3'; reverse: 5'-CGTTAGCGGAATGTCAGCCGTGAAG-3'), GLI3 (forward: 5'-GGCCATCCACATGGAATATC-3'; reverse: 5'-TGAAGAGCTACGGGAAT-3'), PTCH1

(forward: 5'-TGGAAGAAAACAAACAGCTTCC-3'; reverse: 5'-TCCCAGTCACTGTCAAATGC-3'). For analysis of *tulp3* expression in zebrafish, total RNA was isolated from 30 MZ*tulp3* mutant zebrafish embryos or respective control embryos (*tulp3* +/+) at 1 day post fertilization (dpf) using the RNeasy Kit, followed by cDNA synthesis with the Reliance Select cDNA Synthesis Kit (Biorad). The following primers were used for qPCR analysis: tulp3 (forward: 5'-AGAACCTCATCGAGCTGCAT-3'; reverse: 5'-AGAGTGAAGATGTCCTCCGC-3'), ef1α (forward: 5'-TGCCAACTTCAACGCTCAGGT-3'; reverse: 5'-TCAGCAAACTTGCAGGCGATG-3'). The DyNAmo ColorFlash SYBR Green Kit (Thermo Fisher Scientific) was used, and qPCR was performed on a Light Cycler 480 (Roche) at the Lighthouse Core Facility at the University Clinics in Freiburg.

Acknowledgements

We thank the families for participating in this study. We are grateful to Prof. Djengel and Veronica Dumit for discussion and performance of the mass spectroscopy analysis at the Proteomics Core Facility in Freiburg. We thank Alena Sammarco at the Institute of Pathology, University Clinics Freiburg, for substantial help with histological sections and stainings of adult zebrafish tissues. We thank Dr. Elisa Molinari for critical help with patient-derived urinary cell studies. We are grateful to Saikat Mukhopadhyay (UT Southwestern Medical Center) for anti-GPR161 antibodies.

Genomics England Research Consortium (27th May 2021)

John C. Ambrose[1]; Prabhu Arumugam[1]; Roel Bevers[1]; Marta Bleda[1] ; Freya Boardman-Pretty[1,2]; Christopher R. Boustred[1]; Helen Brittain[1]; Mark J. Caulfield[1,2]; Georgia C. Chan[1]; Greg Elgar[1,2]; Tom Fowler[1]; Adam Giess[1]; Angela Hamblin[1]; Shirley Henderson[1,2]; Tim J. P. Hubbard[1]; Rob Jackson[1]; Louise J. Jones[1,2]; Dalia Kasperaviciute[1,2]; Melis Kayikci[1]; Athanasios Kousathanas[1]; Lea Lahnstein[1]; Sarah E. A. Leigh[1]; Ivonne U. S. Leong[1]; Javier F. Lopez[1]; Fiona Maleady-Crowe[1]; Meriel McEntagart[1]; Federico Minneci[1]; Loukas Moutsianas[1,2]; Michael Mueller[1,2]; Nirupa Murugaesu[1]; Anna C. Need[1,2]; Peter O'Donovan[1]; Chris A. Odhams[1]; Christine Patch[1,2]; Mariana Buongermino Pereira[1]; Daniel Perez-Gil[1]; John Pullinger[1]; Tahrima Rahim[1]; Augusto Rendon[1]; Tim Rogers[1]; Kevin Savage[1]; Kushmita Sawant[1]; Richard H. Scott[1]; Afshan Siddiq[1]; Alexander Sieghart[1]; Samuel C. Smith[1]; Alona Sosinsky[1,2]; Alexander Stuckey[1]; Mélanie Tanguy[1]; Ana Lisa Taylor Tavares[1]; Ellen R. A. Thomas[1,2]; Simon R. Thompson[1]; Arianna Tucci[1,2]; Matthew J. Welland1; Eleanor Williams[1]; Katarzyna Witkowska[1,2]; Suzanne M. Wood[1,2].

[1] Genomics England, London, UK.

[2] William Harvey Research Institute, Queen Mary University of London, London, EC1M 6BQ, UK.

Supplementary Web Resources

ConsensusPathDB interaction database: http://consensuspathdb.org/

Clustal omega: https://www.ebi.ac.uk/Tools/msa/clustalo/

STRING: https://string-db.org/

Supplementary References

1. Lu, H., Galeano, M.C.R., Ott, E., Kaeslin, G., Kausalya, P.J., Kramer, C., Ortiz-Brüchle, N., Hilger, N., Metzis, V., Hiersche, M., et al. (2017). Mutations in DZIP1L, which encodes a ciliary-transition-zone protein, cause autosomal recessive polycystic kidney disease. Nat. Genet. *49*, 1025–1034.

2. Schrezenmeier, E., Kremerskothen, E., Halleck, F., Staeck, O., Liefeldt, L., Choi, M., Schüler, M., Weber, U., Bachmann, N., Grohmann, M., et al. (2021). The underestimated burden of monogenic kidney disease in adults waitlisted for kidney transplantation. Genet. Med. Off. J. Am. Coll. Med. Genet. *23*, 1219–1224.

3. Rehm, H.L., Bale, S.J., Bayrak-Toydemir, P., Berg, J.S., Brown, K.K., Deignan, J.L., Friez, M.J., Funke, B.H., Hegde, M.R., Lyon, E., et al. (2013). ACMG clinical laboratory standards for next-generation sequencing. Genet. Med. *15*, 733–747.

4. Matthijs, G., Souche, E., Alders, M., Corveleyn, A., Eck, S., Feenstra, I., Race, V., Sistermans, E., Sturm, M., Weiss, M., et al. (2016). Guidelines for diagnostic next-generation sequencing. Eur. J. Hum. Genet. EJHG *24*, 2–5.

5. Sobreira, N., Schiettecatte, F., Valle, D., and Hamosh, A. (2015). GeneMatcher: a matching tool for connecting investigators with an interest in the same gene. Hum. Mutat. *36*, 928–930.

6. Li, H. (2013). Aligning sequence reads, clone sequences and assembly contigs with BWA-MEM. ArXiv Genomics.

7. McKenna, A., Hanna, M., Banks, E., Sivachenko, A., Cibulskis, K., Kernytsky, A., Garimella, K., Altshuler, D., Gabriel, S., Daly, M., et al. (2010). The Genome Analysis Toolkit: a MapReduce framework for analyzing next-generation DNA sequencing data. Genome Res. *20*, 1297–1303.

8. DePristo, M.A., Banks, E., Poplin, R., Garimella, K.V., Maguire, J.R., Hartl, C., Philippakis, A.A., del Angel, G., Rivas, M.A., Hanna, M., et al. (2011). A framework for variation discovery and genotyping using next-generation DNA sequencing data. Nat. Genet. *43*, 491–498.

9. Van der Auwera, G.A., Carneiro, M.O., Hartl, C., Poplin, R., Del Angel, G., Levy-Moonshine, A., Jordan, T., Shakir, K., Roazen, D., Thibault, J., et al. (2013). From FastQ data to high confidence variant calls: the Genome Analysis Toolkit best practices pipeline. Curr. Protoc. Bioinforma. *43*, 11.10.1-11.10.33.

10. Epting, D., Senaratne, L.D.S., Ott, E., Holmgren, A., Sumathipala, D., Larsen, S.M., Wallmeier, J., Bracht, D., Frikstad, K.-A.M., Crowley, S., et al. (2020). Loss of CBY1 results in a ciliopathy characterized by features of Joubert syndrome. Hum. Mutat. *41*, 2179–2194.

11. Szklarczyk, D., Gable, A.L., Nastou, K.C., Lyon, D., Kirsch, R., Pyysalo, S., Doncheva, N.T., Legeay, M., Fang, T., Bork, P., et al. (2021). The STRING database in 2021: customizable protein–protein networks, and functional characterization of user-uploaded gene/measurement sets. Nucleic Acids Res. *49*, D605–D612.

12. R Development Core Team (2008). R: A language and environment for statistical computing (Vienna, Austria: R Foundation for Statistical Computing).

13. Gentleman, R.C., Carey, V.J., Bates, D.M., Bolstad, B., Dettling, M., Dudoit, S., Ellis, B., Gautier, L., Ge, Y., Gentry, J., et al. (2004). Bioconductor: open software development for computational biology and bioinformatics. Genome Biol. *5*, R80.

14. Dobin, A., Davis, C.A., Schlesinger, F., Drenkow, J., Zaleski, C., Jha, S., Batut, P., Chaisson, M., and Gingeras, T.R. (2013). STAR: ultrafast universal RNA-seq aligner. Bioinforma. Oxf. Engl. *29*, 15–21.

15. Love, M., Anders, S., and Huber, W. (2014). Differential analysis of count data - the DESeq2 package. p.

16. Kamburov, A., Stelzl, U., Lehrach, H., and Herwig, R. (2013). The ConsensusPathDB interaction database: 2013 update. Nucleic Acids Res. *41*, D793–D800.
